# Trial-of-antibiotics to assist tuberculosis diagnosis in symptomatic adults in Malawi (ACT-TB study): a randomised controlled trial

**DOI:** 10.1016/S2214-109X(23)00052-9

**Published:** 2023-03-14

**Authors:** Titus H Divala, Elizabeth L Corbett, Chikondi Kandulu, Brewster Moyo, Peter MacPherson, Marriott Nliwasa, Neil French, Derek J Sloan, Lingstone Chiume, Masiye John Ndaferankhande, Sanderson Chilanga, Sabina Tazirwa Majiga, Jon Øyvind Odland, Katherine L Fielding

**Affiliations:** a Helse Nord TB Initiative, Kamuzu University of Health Sciences, Blantyre, Malawi; b TB Centre, London School of Hygiene & Tropical Medicine, Bloomsbury, London, UK; c Malawi Liverpool Wellcome Trust Clinical Research Programme, Blantyre, Malawi; d Liverpool School of Tropical Medicine, Liverpool, UK; e Institute of Infection Veterinary and Ecological Science, University of Liverpool, Liverpool, UK; f School of Medicine, University of St Andrews, Fife, Scotland, UK; g Victoria Hospital, NHS Fife, Kirkcaldy, Scotland, UK; h Department of Public Health and Nursing, Norwegian University of Science and Technology, Trondheim, Norway; i School of Health and Wellbeing, University of Glasgow, Glasgow, UK

## Abstract

**Background:**

Clinical practice and diagnostic algorithms often assume that tuberculosis can be ruled out in mycobacteriology-negative individuals whose symptoms improve with a trial-of-antibiotics. We aimed to investigate diagnostic performance, clinical benefit, and antimicrobial resistance using a randomised controlled trial.

**Methods:**

In this three-arm, individually randomised, open-label, controlled trial, we enrolled Malawian adults (aged ≥18 years) attending primary care who reported being unwell for at least 14 days (including cough) with no immediate indication for hospitalisation at Limbe and Ndirande Health Centres in Blantyre. Participants were randomly allocated (1:1:1) to azithromycin (500 mg taken once per day for 3 days), amoxicillin (1 g taken three times per day for 5 days), or standard of care with no immediate antibiotics, stratified by study site. Sputum at enrolment and day 8 was tested for tuberculosis (microscopy, Xpert MTB/RIF, and culture). The primary efficacy outcome was day 8 specificity (percentage with symptom improvement among mycobacteriology-negative participants), and day 29 clinical outcome (death, hospitalisation, or missed tuberculosis diagnosis) among all randomised participants. This study is registered with ClinicalTrials.gov, NCT03545373.

**Findings:**

Between Feb 25, 2019, and March 14, 2020, 5825 adults were screened and 1583 (mean age 36 years; 236 [14·9%] HIV positive) were randomly assigned to standard of care (530 participants), azithromycin (527 participants), or amoxicillin (526 participants) groups. Overall, 6·3% (100 of 1583 participants) had positive baseline sputum mycobacteriology. 310 (79·1%) of 392 patients receiving standard of care reported symptom improvement at day 8, compared with 340 (88·7%) of 383 patients receiving azithromycin (adjusted difference 8·6%, 95% CI 3·9–13·3%; p<0·0004) and 346 (89·4%) of 387 receiving amoxicillin (adjusted difference 8·8%, 4·0–13·6%; p=0·0003). The proportion of participants with day 29 composite clinical outcomes was similar between groups (standard of care 1% [7 of 530 participants], azithromycin 1% [6 of 527 participants], amoxicillin 2% [12 of 526 participants]).

**Interpretation:**

Routine outpatient trial-of-antibiotics during tuberculosis investigations modestly improved diagnostic specificity for mycobacteriologically confirmed tuberculosis but had no appreciable effect on death, hospitalisation, and missed tuberculosis diagnosis. These results confirm the limited benefit of trial-of-antibiotics, presenting an opportunity for discontinuation of trial-of-antibiotics and improved antimicrobial stewardship during tuberculosis screening, without affecting clinical outcomes.

**Funding:**

Northern Norway Regional Health Authority (Helse Nord RHF), Commonwealth Scholarship Commission in the UK, Wellcome Trust, UK Medical Research Council, and the UK Department for International Development.

## Introduction

Tuberculosis caused an estimated 1·6 million deaths in 2021,^1^ and has consistently remained one of the leading causes of death worldwide, with year-on-year increases in mortality rates reported during the COVID-19 pandemic.^2^ Despite advances, with new technologies such as molecular microbiology assays and digital chest radiography with computer-aided diagnosis, there is still no low-cost highly accurate rapid test that provides instrument-free point-of-care diagnosis.^3^, ^4^ Emphasising the importance of diagnostic barriers, over 40% of pulmonary tuberculosis notifications to WHO in 2020 were based on clinical diagnosis without mycobacteriological confirmation.^1^

For decades, clinical tuberculosis diagnosis has used algorithms based on treating symptomatic patients with broadspectrum antibiotics (trial-of-antibiotics) with negligible *Mycobacterium tuberculosis* activity, and considering empirical treatment for tuberculosis if the clinical response is poor.^5^, ^6^, ^7^ Tens of millions of such antibiotic courses are prescribed globally every year, meaning that trial-of-antibiotics is likely to be the most commonly used tuberculosis triage test^8^, ^9^, ^10^ and a potentially important contributor to antimicrobial resistance.^11^, ^12^, ^13^

**Research in context**

**Evidence before this study**

Tuberculosis diagnostic algorithms often recommend prescription of broad-spectrum antibiotics for patients whose initial sputum tests are negative (trial-of-antibiotics), assuming that post-antibiotic symptom improvement rules out tuberculosis. We searched MEDLINE, Embase, and Global Health on Feb 18, 2023, using the combined terms for “tuberculosis”, “antibiotic treatment”, terms for diagnostic accuracy (“sensitivity”, “specificity”, and “predictive value”), and a filter for randomised trials. We searched for randomised trials published from database inception to Feb 18, 2023, with no language restrictions, and found no relevant publications. Our systematic review and meta-analysis that pooled available observational data (only eight studies), concluded that trial-of-antibiotics was yet to be supported by evidence, and the meta-analysis suggested that diagnostic performance was low. Not one of the identified studies systematically assessed the effect on other clinical outcomes and antimicrobial resistance.

**Added value of this study**

To our knowledge, this study is the first randomised trial to investigate the diagnostic, clinical, and the effect on antimicrobial resistance of trial-of-antibiotics to rule out tuberculosis. Compared with standard of care, trial-of-antibiotics with either azithromycin or amoxicillin modestly improved diagnostic specificity for mycobacteriologically confirmed tuberculosis, and provided weak evidence for increased risk of antimicrobial resistance. There was no effect on the composite clinical outcome (coprimary) of missed tuberculosis diagnosis, hospitalisation, or death.

**Implications of all the available evidence**

These results confirm the limited benefit of trial-of-antibiotics in tuberculosis screening algorithms, presenting an opportunity for discontinuation of routine prescription of trial-of-antibiotics and improved antimicrobial stewardship, without affecting clinical outcomes. National tuberculosis and antimicrobial stewardship programmes should restrict prescription of empirical broad-spectrum antibiotics to patients in which strong clinical or microbiological indication exists. New affordable and point-of-care diagnostics for tuberculosis and other respiratory pathogens are urgently needed to address the unmet clinical need.

In addition to uncertain diagnostic performance and concerns about antimicrobial resistance, patient safety and effectiveness in improving clinical outcomes are key underinvestigated considerations when trials-of-antibiotics are used in tuberculosis diagnostic algorithms. The potential for clinical benefit is based on the high risk of bacterial infections in patients undergoing tuberculosis investigations.^14^, ^15^, ^16^ We aimed to conduct a randomised controlled trial (the accuracy and consequences of using trial-of-antibiotics for tuberculosis diagnosis [ACT-TB]) to investigate the effect of a trial-of-antibiotic intervention on diagnostic, clinical, and antimicrobial resistance outcomes in Malawi.^17^

## Methods

### Study design

We conducted this three-arm, individually randomised, open-label, controlled trial among adults who presented with cough for at least 14 days at two primary care centres in Blantyre, Malawi. The study design has been described in detail elsewhere^17^ and the protocol and statistical analysis plan are available in the appendix (pp 5, 84). The study was reviewed and approved by the Kamuzu University of Health Sciences Research and Ethics Committee, London School of Hygiene & Tropical Medicine Research Ethics Committee, Regional Committee for Health and Research Ethics–Norway, and Malawi Pharmacy, Medicines, and Poisons Board (appendix p 106).

### Participants

We introduced the study to adults presenting to either Limbe or Ndirande Health Centres in Blantyre, Malawi, by first inviting all participants with a cough to a brief talk, and then conducted a detailed eligibility screen for those who expressed interest. We included patients who were aged at least 18 years, who had a cough, who reported being unwell for at least 14 days, and who did not have any danger signs (respiratory rate ≥30 breaths per min, temperature ≥39°C, heart rate ≥120 beats per min, confusion or agitation, respiratory distress, and systolic blood pressure of <90 mm Hg). We excluded patients who reported allergies to study medications, who had taken antibiotics other than co-trimoxazole prophylaxis within the previous 14 days, or had taken tuberculosis drugs either for treatment or prevention within the previous 6 months. To participate, eligible patients provided written (or, if not literate, witnessed thumbprint) informed consent.

### Randomisation and masking

We used block-randomisation with variable block sizes, stratified by study site, to allocate participants (1:1:1) to either standard of care (no study antibiotic prescription), azithromycin (azithromycin 500 mg taken once per day for 3 days, from enrolment day, termed day 1), or amoxicillin (amoxicillin 1 g taken three times per day for 5 days, from day 1). An independent statistician prepared a randomisation list using the ralloc command in Stata. Allocations were sealed in sequentially numbered opaque envelopes, opened, and assigned by site staff upon confirming eligibility. The dosage of antibiotic groups and self-administration were explained, and participants took their first dose in the presence of study staff at the clinic with the remainder self-administered at home. The study was not blinded, but mycobacteriology and antimicrobial resistance outcome assessment occurred without reference to group.

### Procedures

The standard of care group of no antibiotics until clinically indicated was based on national and global guidelines.^18^ We chose amoxicillin because it is the standard first-line treatment used for trial-of-antibiotics in Malawi, and in line with the WHO access, watch, reserve (AWaRe) classification.^19^ However, amoxicillin might not show the best performance for trial-of-antibiotics because of increasing resistance, and a narrow coverage for causes of community-acquired pneumonia and atypical organisms. We therefore included azithromycin as a third group to represent the optimal biological specificity of an oral regimen due to more complete coverage of atypical organisms that cause community-acquired pneumonia (eg, *Mycoplasma pneumoniae* and *Chlamydia pneumoniae*), and the low resistance rates in Malawi.

Treatments were commenced following randomisation, and outcomes were ascertained on day 8 and day 29. Clinical and laboratory procedures at days 8 and 29 are described in the published protocol paper.^17^

### Outcomes

We had two coprimary outcomes focused on diagnostic accuracy and clinical effect. The diagnostic effect primary outcome was defined as the proportion of participants without tuberculosis (negative reference standard) correctly identified by the index test at day 8 (appendix p 1). The index test was defined as positive (improvement) or negative (no improvement, or no change or worsened) in response to the question: on day 1, you reported that you were unwell; compared to that day, has your illness worsened, remained the same, or improved? The reference standard was defined as positive if at least one day 1 or day 8 sputum sample was positive on smear microscopy, Xpert MTB/RIF (Cepheid, CA, USA) or culture, and negative if none were positive and at least one test was known to be negative. Participants received their tuberculosis test results after completion of the day 8 visit and, where applicable, tuberculosis treatment followed. The clinical effect coprimary outcome was a composite measure defined as the risk of any of death, hospitalisation, or missed tuberculosis diagnosis by day 29 among all randomised participants. Missed tuberculosis diagnosis was defined as tuberculosis not detected on day 1 or day 8 sputum but documented based on day 29 mycobacteriology or radiological findings consistent with tuberculosis.

Pre-specified secondary outcomes were diagnostic accuracy in participants who could not produce sputum at day 1 and day 8, and antimicrobial resistance. We included the secondary diagnostic accuracy outcome because, in the study setting, as many as 13% of symptomatic adults do not produce sputum.^20^ We defined the antimicrobial resistance secondary outcome as the proportion of all randomised participants whose day 29 nasopharyngeal swabs grew *Streptococcus pneumoniae* resistant to any of the following commonly used antibiotics selected from all three WHO AWaRe classes:^19^ ceftriaxone, amoxicillin, cefoxitin, azithromycin, and erythromycin, as determined using the disk diffusion technique. *S pneumoniae* was chosen as a sentinel respiratory pathogen, firstly because it can acquire resistance efficiently through DNA uptake,^21^, ^22^ and secondly, because the treatment of choice for these *S pneumoniae* are penicillins and macrolides, which are the same drugs used as study interventions. In a post-hoc analysis we considered only incident resistant isolates, excluding participants who had resistant isolates at baseline.

### Statistical analysis

The statistical approach is described in the statistical analysis plan included in the appendix (p 84). For the diagnostic effect primary outcome, we assumed that day 8 symptom improvement in trial-of-antibiotics (azithromycin or amoxicillin) groups would correctly classify 60% of all mycobacteriology-negative participants (ie, 60% specificity).^23^ We established that 388 of 1164 reference standard-negative participants per group would provide 80% power at a two-sided type 1 error of 5%, to detect at least a 10% difference in specificity. To achieve the required 1164 mycobacteriology- negative participants (rounded to 400 per group), we accounted for tuberculosis prevalence (20%), inability to produce sputum (15%), and day 8 loss to follow-up (5%), increasing the target recruitment to 625 per group or 1875 in total. For the clinical effect outcome, we assumed a 4% risk of the composite clinical outcome in the standard of care group, and a loss to follow-up of 10% by day 29. Assuming 625 participants per group and a two-sided type I error of 5%, we would have 80% power to detect a risk ratio of at least 2, comparing either intervention group to standard of care.

All analyses were done using the group to which the participant was randomised. We report measures of effect for comparing azithromycin or amoxicillin groups separately and combined, with the standard of care. We used a generalised linear model with identity-link function to estimate risk differences. Our a priori design did not adjust for multiple comparisons but reported all intervention effects with their 95% CI and p values to facilitate appropriate interpretation.^24^ The analysis was performed using Stata.

In the pre-specified analysis, we calculated test characteristics (sensitivity and area under the receiver operating characteristic curve) of trial-of-antibiotics versus the sputum mycobacteriological reference standard, and their respective 95% CIs. Post-hoc analyses were also conducted for sensitivity including: (1) all mycobacteriology from day 1, day 8, and day 29; and (2) in participants with tuberculosis clinical diagnoses, defined as initiation of tuberculosis treatment in mycobacteriology-negative patients based on routine clinical or radiological diagnosis, for the reference standard.

In our pre-specified subgroup analysis, we examined the diagnostic performance by HIV status. We did not conduct a pre-specified subgroup analysis for the clinical effect primary outcome because of a low number of events. In post-hoc per-protocol analyses for diagnostic and antimicrobial resistance effect, we excluded participants who reported incomplete adherence to treatment (remaining with at least one study tablet by day 8) or taking non-study antibiotics by day 8.

### Role of the funding source

The funders of the study had no role in study design, data collection, data analysis, data interpretation, or writing of the report.

## Results

Between Feb 25, 2019, and March 14, 2020, we screened 5825 adults presenting with cough to Limbe and Ndirande Health Centres, Malawi, of whom 2659 (45·6%)expressed interest after a brief description of the study. 1076 participants were ineligible, with most common reasons being recent antibiotic treatment (503 [46·7%] of 1076), recent or current tuberculosis preventive treatment (198 [18·4%]), and being aged younger than 18 years (95 [8·8%]). 43 (4%) of 1076 adults were eligible but did not give consent. 1583 (27·2%) of 5825 met the eligibility criteria, gave written consent to participate, and were randomly assigned (530 to standard of care, 527 to azithromycin, and 526 to amoxicillin (figure). HIV prevalence was 14·9% (236 of 1583), with 214 (97·7%) of 219 of the previously diagnosed patients already taking antiretroviral therapy (ART) (table 1). The participant recruitment period preceded the earliest known COVID-19 infections in Blantyre, Malawi.^25^

**Figure:**
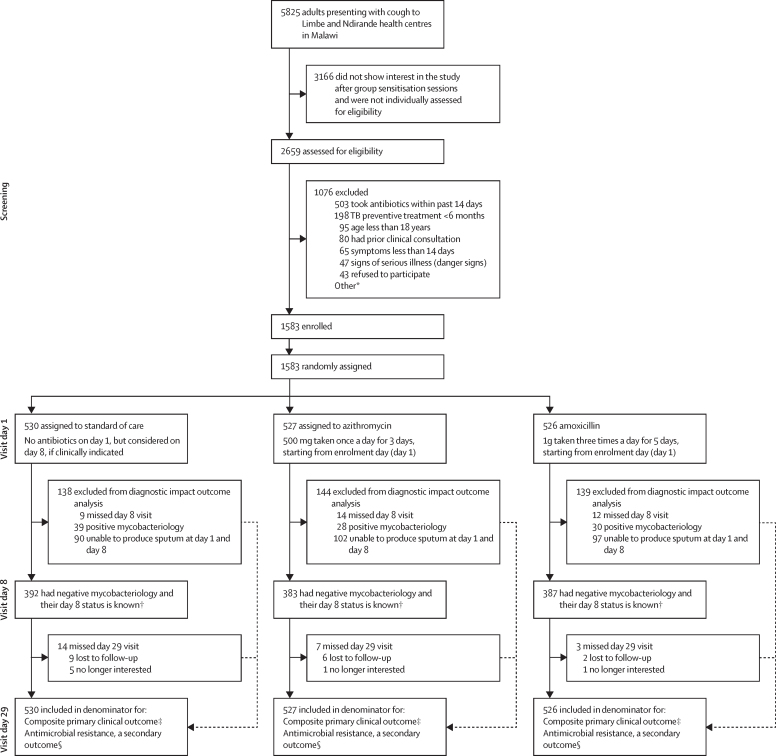
Trial profile *Unable to return for follow-up visits (42 [4%]), lives outside the study catchment areas (25 [2%]), unable to walk independently (13 [1%]), took tuberculosis treatment in the past 6 months (12 [1%]), and reported an allergy to study medication (1 [<1%]). †Denominator for diagnostic accuracy primary outcome. ‡Experiencing either death, hospitalisation, or missed tuberculosis diagnosis by day 29. §Nasopharyngeal swab with *Streptococcus pneumoniae* resistant to commonly used antibiotics.

**Table 1:** Baseline characteristics of the randomised population

		**Standard of care (n=530)**	**Azithromycin (n=527)**	**Amoxicillin (n=526)**
Age, years		36·4 (15·9)	35·6 (13·8)	35·7 (14·8)
Research site				
	Limbe Health Centre	169 (32%)	167 (32%)	168 (32%)
	Ndirande Health Centre	361 (68%)	359 (68%)	357 (68%)
Sex				
	Female	323 (61%)	302 (57%)	319 (61%)
	Male	207 (39%)	224 (43%)	206 (39%)
Presenting symptoms and history				
	Fever	326 (62%)	343 (65%)	322 (61%)
	Night sweats	241 (45%)	246 (47%)	228 (43%)
	Chest pain	387 (73%)	386 (73%)	381 (72%)
	Blood in sputum	34 (6%)	20 (4%)	24 (5%)
	Self-reported weight loss	191 (36%)	183 (35%)	183 (35%)
	Previous tuberculosis	42 (8%)	26 (5%)	32 (6%)
	Months since last tuberculosis treatment	126·1 (42·6–221·2)	94·7 (59·3–129·8)	208·6 (65·5–296·3)
	Pregnant	20 (4%)	13 (2%)	13 (2%)
Baseline assessments and investigations				
	BMI <19 kg/m^2^	52 (10%)	55 (10%)	47 (9%)
	HIV positive*	83 (16%)	73 (14%)	80 (15%)
	On ART†	75 (14%)	67 (13%)	72 (14%)
	Antimicrobial resistance positive swab	45 (8%)	42 (8%)	52 (10%)

Data are n (%), median (IQR), or mean (SD). ART=antiretroviral therapy.

* HIV status unknown in 55 participants (25 in the standard of care group, 14 in the azithromycin group, and 16 in the amoxicillin group), and HIV was newly diagnosed for 17 of 122 participants tested (six of 43 participants in the standard of care group, four of 37 participants in the azithromycin group, and seven of 42 participants in the amoxicillin group).

† 97·7% (214 of 219 participants) of the previously diagnosed HIV-positive participants were already on ART.

Out of the randomised 1583 participants 1171 (74·0%) provided a sputum sample for day 1 Xpert MTB/RIF, 1181 (51·7%) for day 1 smear microscopy and culture, 818 for day 8 smear microscopy and culture, and 65 (4·1%) for unscheduled day 29 smear microscopy and culture based on clinical need (appendix p 2). The prevalence of tuberculosis by day 8 (positive mycobacteriology at day 1, day 8, or both) was 6·3% (100 of 1583). By day 29, an additional five mycobacteriologically confirmed cases and 28 clinically diagnosed cases were identified giving a prevalence of 8·4% (133). We recorded 12 serious adverse events (appendix p 4), which included four deaths and eight hospitalisations. None of the events was related to the study treatment. All events were reported to the data and safety monitoring board.

More participants missed the day 8 visit (35 [3%] of 1583) than day 29 visit (24 [1·5%]; figure). Reasons for missing the day 8 visit were not systematically recorded, but 24 participants missed the day 29 visits due to either loss to follow-up (71% [17 of 24]) or withdrawal of consent (29% [7]).

1162 participants with negative mycobacteriology and known day 8 symptom status contributed to the diagnostic accuracy primary outcome (392 [34%] of 1162 in the standard of care group, 383 [33%] in the azithromycin group, and 387 [33%] in the amoxicillin group). This sample size achieved the required number of tuberculosis-negative participants per group (around 388 participants) without needing the planned full recruitment of 625 per group (or 1875 total) because tuberculosis prevalence had declined from the anticipated 20% to 6%.

310 (79·1%) of 392 patients receiving standard of care reported symptom improvement at day 8, compared with 340 (88·7%) of 383 patients receiving azithromycin (adjusted difference 8·6%, 95% CI 3·9–13·3%; p<0·0004) and 346 (89·4%) of 387 receiving amoxicillin (adjusted difference 8·8%, 4·0–13·6%; p=0·0003; table 2). In the subgroup analysis, diagnostic effect did not vary by HIV status.

**Table 2:** Diagnostic effect of trial-of-antibiotics (primary, secondary, and pre-specified outcomes)

		**Standard of care**	**Azithromycin**	**Amoxicillin**
**Primary outcome**				
Specificity among participants with sputum mycobacteriology test results*		310/392 (79·1%)	340/383 (88·8%)	346/387 (89·4%)
Difference (95% CI); p value		..	8·6% (3·9 to 13·3); p<0·0004	8·8% (4·0 to 13·6); p=0·0003
**Secondary outcome**				
Specificity with participants without sputum classified as tuberculosis negative		398/482 (82·6%)	439/485 (90·5%)	441/484 (91·1%)
Difference (95% CI); p value		..	6·6% (2·7 to 10·4); p=0·0006	6·8% (2·9 to 10·7); p=0·0005
**Pre-planned subgroup analysis**				
HIV status†				
	HIV positive	54/64 (84·4%)	52/54 (96·3%)	59/67 (88·1%)
	Difference (95% CI); p value	..	11·9% (1·7 to 22·1); p=0·022	3·7% (−8·1 to 15·5); p=0·61
	HIV negative	238/307 (77·5%)	278/315 (88·3%)	275/307 (89·6%)
	Difference (95% CI); p value	..	10·7% (4·9 to 16·6); p=0·0012	12·1% (6·2 to 17·8); p=0·00004
	Interaction p value for treatment group and HIV status	..	p=0·85	p=0·21

Data are n/N (%), unless otherwise stated. n=number of participants with outcome for the index test (day 8 self-reported symptom status) defined as positive (improvement) or negative (no improvement, no change, or worsened).

* Mycobacteriology based on day 1 and day 8 smear microscopy, Xpert/MTB/Rif, and tuberculosis culture, in which positive was defined as sputum positive on at least one test, and negative if none of the tests is positive and at least one is known to be negative. All risk differences are adjusted for study site.

† Specificity by HIV status among participants with sputum results.

When participants who could not produce sputum were included in the denominator for the diagnostic effect outcome (diagnostic accuracy secondary outcome), trial-of-antibiotics with either azithromycin (439 [90·5%] of 485) or amoxicillin (441 [91·1%] of 484) still showed improvement in specificity compared with standard of care (398 [82·6%] of 482), although the effect was smaller than in the primary outcome analysis (table 2).

The sensitivity of the three arms against the primary reference mycobacteriology was 25·6% (10 of 39 patients; 95% CI 13·0–42·1) for standard of care, 10·7% (3 of 28 patients; 2·3–28·2) for azithromycin, and 23·3% (7 of 30 patients; 9·9–42·3) for amoxicillin (appendix p 3). In post-hoc analyses, the diagnostic sensitivity remained very low and similar to the standard of care (standard of care 25·0% [95% CI 12·7–41·2%], azithromycin 10·7% [2·3–28·2%], and amoxicillin 22·6% [9·6–41·1%]) when all mycobacteriology from day 1, day 8, and day 29 were included in the reference standard, and did not improve (standard of care 26·1% [95% CI 14·3–41·1%], azithromycin 15·8% [6·0–31·3%], and amoxicillin 23·8% [12·1–39·5%]) after including clinical diagnoses (appendix p 3).

Compared with standard of care (7 [1·3%] of 530), the percentage of participants who experienced the day 29 composite clinical outcome (at least one of death, hospitalisation, or missed tuberculosis diagnosis) did not differ by group (azithromycin –0·2%, 95% CI –1·5 to 1·1; and amoxicillin 1·0%, –0·6 to 2·6; table 3).

**Table 3:** The effect of trial-of-antibiotics on the clinical coprimary outcome and secondary outcome of antimicrobial resistance

	**Standard of care (n=530)**	**Azithromycin (n=527)**	**Amoxicillin (n=526)**
**Primary outcome**			
Composite clinical endpoint of missed tuberculosis diagnosis, hospitalisation, and death by day 29	7/530* (1·3%)	6/527* (1·1%)	12/526* (2·3%)
Risk difference (95% CI); p value	..	−0·2% (−1·5 to 1·1); p=0·79	1·0% (−0·6 to 2·6); p=0·24
**Individual components of the composite clinical endpoint**†			
Missed tuberculosis diagnosis	3/530 (0·6%)	3/527 (0·6%)	7/526 (1·3%)
Hospitalisation	3/530 (0·6%)	3/527 (0·6%)	3/526 (0·6%)
Death	2/530 (0·4%)	0	2/526 (0·4%)
**Secondary outcome**			
Antimicrobial resistance positive at day 29‡	28/530 (5·2%)	41/527 (7·8%)	27/526 (5·1%)
Risk difference (95% CI); p value	..	2·5% (−0·5 to 5·5); p=0·10	−0·2% (−2·9 to 2·5); p=0·90

Data are n/N (%), unless otherwise stated. All risk differences and ratios are adjusted for study site.

* Number randomised.

† It was possible for an individual participant to have more than one of the three components of the composite clinical outcomes.

‡ Number of participants who provided nasopharyngeal swab samples for *Streptococcus pneumoniae* culture.

1529 of 1583 participants (96·6% of the total randomised) provided day 29 nasopharyngeal swab samples, of which 10·9% (167 of 1529) grew *S pneumoniae* (standard of care 55 [10·9%] of 506, azithromycin 57 [11·1%] of 512, amoxicillin 55 [10·8%] of 511). Overall, 57·5% (96 of 167) of the isolated *S pneumoniae* were resistant to at least one of the commonly used antibiotics (ceftriaxone, amoxicillin, cefoxitin, azithromycin, and erythromycin; table 3).

Compared with standard of care, the proportions of participants whose day 29 nasopharyngeal swabs grew *S pneumoniae* resistant to at least one commonly used antibiotic were 2·5 percentage points higher in the azithromycin group (risk difference 2·5%, 95% CI –0·5 to 5·5; p=0·10).

The percentage of day 29 resistant isolates in the amoxicillin group was similar (risk difference –0·2%, 95% CI –2·9 to 2·5; p=0·90) to that of standard of care. In a post-hoc analysis excluding 139 participants who had resistant isolates at baseline, the percentages of antimicrobial resistance positive at day 29 were 21 of 485 in the standard of care group, 36 of 485 in the azithromycin group, and 18 of 474 in the amoxicillin group. Risk differences, adjusted for study site, were 3·1% (95% CI 0·1 to 6·1; p=0·04) for the azithromycin group and –0·6 (95% CI –3·1 to 1·9; p=0·66) for the amoxicillin group versus the standard of care group.

More participants (118 [23·1%] of 511) in the amoxicillin group (dosage was 12 tablets per day over 5 days) remained with at least one unused study medication tablet by day 8, compared with the azithromycin group (11 [2·2%] of 511; dosage was two tablets per day over 3 days; table 4). Participants from all three groups (standard of care 62 [11·7%] of 530 participants, azithromycin 22 [4·2%] of 527 participants, and amoxicillin 16 [3·0%] of 526 participants) received non-study antibiotics before their day 8 visit. Post-hoc per-protocol analyses of the diagnostic and antimicrobial resistance outcomes excluding participants who took non-study antibiotics and those who missed any single study drug tablet produced results similar to the main analyses (table 4).

**Table 4:** Diagnostic and antimicrobial resistance effect among participants who adhered* to study interventions

	**Standard of care (n=530)**	**Azithromycin (n=527)**	**Amoxicillin (n=526)**
**Treatment adherence**			
Missed at least one tablet*†	Not defined	11/511 (2·2%)	118/511 (23·1%)
Took non-study antibiotics between day 1 and day 8	62/530 (11·7%)	22/527 (4%)	16/526 (3%)
Took at least one study or non-study antibiotic course between day 1 and day 29	80/530 (15·1%)	527/527 (100·0%)	526/526 (100·0%)
Took at least two antibiotic courses during study period	9/530 (1·7%)	33/527 (6·3%)	28/526 (5·3%)
Took at least three antibiotic courses during study period	4/530 (0·8%)	6/527 (1·1%)	8/526 (1·5%)
Took at least four antibiotic courses during study period	0	2/527 (<1%)	2/526 (<1%)
Number of occasions a course of antibiotics was taken	93	572	570
Number of occasions a non-study course of antibiotics was taken	93	41	38
**Post-hoc per-protocol analysis (including only participants who adhered to treatment group)**			
Diagnostic specificity at day 8‡	282/336 (83·9%)	322/357 (90·2%)	262/288 (91·0%)
Difference in specificity between groups (95% CI); p value	..	6·8% (2·1 to 11·5); p=0·0050	7·4% (2·6 to 12·3); p=0·0032
Antimicrobial resistance by day 29§	22/449 (4·9%)	36/483 (7·5%)	18/384 (4·7%)
Antimicrobial resistance risk difference (95% CI); p value	..	2·6% (−0·5 to 5·6); p=0·10	−0·2% (−3·1 to 2·7); p=0·88

Data are n/N (%), unless otherwise stated.

* The question was: out of all the study medication tablets we gave you, are there any remaining?

† Denominator is the number of participants who completed the study medication adherence questionnaire in audio computer assisted self-interview at day 8.

‡ Adherence to treatment defined as not missing any single study drug and not taking any non-study antibiotic before or on day 8.

§ Adherence to treatment defined as not missing any single study drug and not taking any non-study antibiotic by day 29.

## Discussion

The main findings of this individually randomised trial to investigate the diagnostic, clinical, and antimicrobial resistance effect of trial-of-antibiotics during tuberculosis investigations were that, compared with standard of care, trial-of-antibiotics with either azithromycin or amoxicillin improved diagnostic specificity for mycobacteriologically confirmed tuberculosis. However, routine prescription of antibiotics did not improve day 29 clinical outcomes (all-cause mortality, hospitalisation, and missed diagnosis of tuberculosis), and might have generated antimicrobial resistance in the azithromycin group.

The improvement in diagnostic specificity shown by trial-of-antibiotics using either azithromycin or amoxicillin confirms the long-established clinical rationale for national and global guidelines for addressing the suboptimal nature of tuberculosis diagnosis. Our randomised trial further adds to the body of evidence by quantifying the magnitude of the diagnostic benefit allowing for a comparison against the WHO criteria (target product profiles) for tuberculosis diagnostic test performance.^26^ The WHO target product profiles use specificity of more than 80% and sensitivity more than 95% as benchmarks for an ideal triage test, and specificity of more than 98% and sensitivity more than 95% for smear microscopy-replacement test.^26^ With respect to specificity (primary outcome), the 88·8% (340 of 383) recorded in the azithromycin group and the 89·4% (346 of 387) in the amoxicillin group meet the target for triage test but fall below that of smear microscopy-replacement tests aimed for health facility use.

In this study, the limited nature of the diagnostic benefit is further underscored by the extremely low sensitivity for both azithromycin (three [10·7%] of 28 participants) and amoxicillin (seven [23·3%] of 30 participants; appendix p 3). We did not include sensitivity as a trial outcome, due to the anticipation of much lower numbers of sputum-confirmed patients than sputum-negative patients but did anticipate sufficient power to provide reasonable precision around the point estimate of sensitivity per group for which at least 30 confirmed patients per group is required. These estimates fall well below target, even when only the upper 95% CIs are considered (28·2% for azithromycin, and 42·3% for amoxicillin) against the minimum acceptable target of 90% for triage test, and more than 80% for smear microscopy-replacement tests.

Apart from diagnostic performance, safety is the other key consideration for national programmes and clinicians before routine prescription of trial-of-antibiotics to outpatients without danger signs can be discontinued. Withholding a course of effective antibiotic treatment could affect patient safety because bacterial causes of illness in patients with respiratory symptoms are common,^14^, ^27^ and are an important cause of hospitalisation and mortality.^14^, ^15^, ^16^ The lack of difference in the risk of death, hospitalisations, and missed tuberculosis diagnosis between participants in the standard of care group and those taking trial-of-antibiotics (azithromycin or amoxicillin groups) is reassuring and strengthens the argument for discontinuation of routine prescription of broad-spectrum antibiotics to outpatients with respiratory symptoms. However, the lack of difference could also be explained by the fact that we registered very low morbidity and mortality, which reduced the study power below anticipated for this outcome.

Our safety data results are consistent with those from a 2017 systematic review update comparing immediate prescription with antibiotic-sparing strategies for outpatients with uncomplicated acute respiratory infections.^28^ That review reported no difference in clinical outcomes between delayed, immediate, and no prescribed antibiotics for patients with cough, but was based on only four studies with limited geographical range.^28^ Our study adds to the available data on people living with HIV, a major factor affecting cause, management, and prognosis of acute respiratory infection.^29^ 14·9% of participants in this trial were HIV positive. However, our results might not be generalisable to settings with high HIV prevalence with lower coverage of diagnosis and ART because very few (6%) were newly diagnosed and 97% of those previously diagnosed were already taking ART. We did not conduct a predefined subgroup analysis of clinical outcomes by HIV status due to the low event rate.

We additionally found results consistent with previous reports of rapid acquisition of resistance following brief exposure to azithromycin,^30^, ^31^ with the risk of resistant nasal pneumococcal isolates being 2·5% (95% CI –0·5 to 5·5) higher for patients randomised to receive azithromycin compared with standard of care. The difference was greater when patients with pre-existing (baseline) antimicrobial resistance were excluded from the analysis (3·1%, 95% CI 0·1 to 6·1). These results add to the already existing strong body of evidence on the association between empirical antibiotic treatment and emergence of antimicrobial resistance.^32^, ^33^, ^34^ We saw no similar suggestion of rapid emergence of resistance in the amoxicillin group, despite higher pre-existing rates of resistance in Blantyre, Malawi, and lower treatment adherence for amoxicillin compared with azithromycin.^35^ Unlike amoxicillin, azithromycin has a long half-life^36^ due to extensive uptake in tissues,^36^, ^37^ which has been postulated to lead to a wide mutant selection window (drug concentration range in which resistant mutants are selectively amplified), potentially allowing greater mutant amplification than is seen with amoxicillin.^30^, ^38^

Antibiotic prescription for patients presenting with respiratory symptoms was common practice at the study sites, with 46·7% (503 of 1076) of the potential participants excluded due to having taken antibiotics within 14 days, and self-reported use of non-study antibiotics by day 29 reported by 15·1% (80 of 530) of standard of care group participants. The most common non-study antibiotic was amoxicillin, consistent with previous reports of wide availability and easy access in Malawi.^39^ Taking non-study antibiotics would tend to drive our antimicrobial resistance day 29 measures of effect towards the null by increasing risk of resistance (in the standard of care group) or by clearing carriage (trial-of-antibiotic groups). However, post-hoc analysis of the antimicrobial resistance outcome excluding participants who reported missing at least one tablet of their study medication, and those who reported taking any non-study antibiotic by day 29, did not have any effect on effect measures.

Our main study limitations include no blinding and consequential room for misclassifying exposure status for participants who could have accessed antibiotics outside the study beyond that reported, and possible underdiagnosis of active tuberculosis status. However, exploratory analysis of different tuberculosis diagnostic criteria and exclusion of patients known to have taken antibiotics outside of the study prescriptions do not support a major impact on our key conclusions. Another potential source of misclassification of exposure status is the self-administration of study drugs, but this reflects clinical practice for oral treatment in ambulatory adults. Provision of daily dosing oral antibiotics under directly observed treatment approach is not routinely practised for outpatients as it might not be practical. We recruited participants from a single city, limiting generalisation to other settings. We did not power the study on sensitivity, limiting our ability to compare differences between groups to specificity. However, the upper 95% CI for our point estimates of sensitivity fell well below the WHO target ranges for both antibiotic groups, suggesting that trial-of-antibiotics is unlikely to meet diagnostic acceptability criteria in this respect.

In conclusion, our results do not support routine prescription of trial-of-antibiotics for the purposes of establishing a diagnosis of tuberculosis in ambulatory adult outpatients. Antibiotic prescriptions can be reduced for adult outpatients with symptoms suggestive of tuberculosis without affecting patient clinical outcomes. Policy and research should urgently establish antibiotic-sparing diagnostic approaches for primary care management of respiratory symptoms.

## Data sharing

The study protocol, standard operating procedures, and the ethics and regulatory information can be accessed from: https://amr.tghn.org/study-profiles/act-tb-malawi. De-identified individual participant data will be made available upon provision of a justified request to the corresponding author.

## Declaration of interests

We declare no competing interests**.**
