# Supplementary material for: Trial-of-antibiotics to assist tuberculosis diagnosis in symptomatic adults in Malawi (ACT-TB study): a randomised controlled trial
Source: Lancet Glob Health. 2023 Mar 14;11(4):e556–65. doi: 10.1016/S2214-109X(23)00052-9 (PMC10030459; doi:10.1016/S2214-109X(23)00052-9)
Supplement: Supplementary appendix [file mmc1.pdf]

THE LANCET
Global Health

Supplementary appendix

This appendix formed part of the original submission and has been peer reviewed. We post it as supplied by the authors.

Supplement to: Divala TH, Corbett EL, Kandulu C, et al. Trial-of-antibiotics to assist tuberculosis diagnosis in symptomatic adults in Malawi (ACT-TB study): a randomised controlled trial. *Lancet Glob Health* 2023; **11:** e556–65.

Appendix

Supplementary figures and tables

Trial of antibiotics to assist tuberculosis diagnosis in symptomatic adults in Malawi: a randomised controlled trial (ACT-TB study)

REFERENCE STANDARD RESULT
Mycobacteriology based on Day 1 and Day 8
smear microscopy, Xpert/MTB/Rif and tuberculosis Culture)

		**POSITIVE** (positive on any of the three tests)	**NEGATIVE** (none of the tests positive, but negative on at least one)	**Could not produce sputum**
**INDEX TEST RESULT** **Self reported Clinical response on audio computer-assisted self-interview conducted on Day 8**	**POSITIVE** (Reporting no improvement or worsening symptoms)	a	b	e
	**NEGATIVE** (Reporting improvement of symptoms)	c	d	f

**Primary outcome:** Proportion of reference standard negative participants correctly identified by the index test (reporting improvement of symptoms) **d/(b+d)**
**Secondary outcome** (diagnostic specificity in broader population**):** participants who could not produce sputum at both time points classified as reference standard negative, before estimating **(d+f)/(b+d+e+f)**

Supplementary figure 1. Definition for primary and secondary outcomes of the diagnostic impact of trial-of-antibiotics versus mycobacteriology.

Supplementary table 1. Distribution of reference standard for primary outcome, by individual diagnostic test (day 1 and day 8)

		standard of care	azithromycin	amoxicillin
**Reference Standard positive**	One culture and any other test	26	19	13
	Any two of Xpert or Smear	0	1	1
	Only one Culture	11	6	13
	Only one Xpert	2	2	2
	Only one Smear	0	0	1
	Total	39	28	30
**Reference Standard Negative**	One culture and any other test	373	368	371
	Only one Xpert	19	15	16
	Total	392	383	387
**Reference Standard undefined**	Could not produce a sputum sample on both day 1 and day 8	90	102	97
	missed outcome assessment visit *	9	14	12
	Total	99	116	109
*Study visit day 8 where audio computer assisted self-interview was conducted to record change in symptoms compared to baseline. Culture = MTB culture. Xpert= Xpert MTB/RIF. Smear= smear microscopy.				

Supplementary table 2. Diagnostic performance of trial of antibiotics versus a reference standard containing all mycobacteriology up to day 29, with and without including clinical tuberculosis

Study arm (number randomised)	standard of care	azithromycin	amoxicillin
**Reference standard: day 1 and day 8 sputum mycobacteriology (pre-specified analysis)**			
Sensitivity % (n/N, [95%CI])	25.6% (10/39 [13.0, 42.1])	10.7% (3/28 [2.3, 28.2])	23.3% (7/30 [9.9, 42.3])
Area under the receiver operating curve (95%CI)	0.52 (0.45, 0.60)	0.50 (0.44, 0.56)	0.56 (0.49, 0.64)
**Reference standard: day 1, day 8, and day 29 sputum mycobacteriology (post post hoc analysis)**			
Sensitivity % (n/N, [95%CI])	25.0% (10/40 [12.7%, 41.2%])	10.7% (3/28 [2.3%, 28.2%])	22.6% (7/31 [9.59%, 41.1%])
Specificity % (n/N, [95%CI])	82.5% (84/481 [78.8%, 85.8%])	90.5% (439/485 [87.6%, 93.0%])	91.1% (440/483 [88.2%, 93.5%])
Area under the receiver operating characteristic curve (95%CI)	0.54 (0.47, 0.61)	0.51 (0.45, 0.57)	0.57 (0.49, 0.64)
**Reference standard: day 1, day 8, and day 29 sputum mycobacteriology plus clinical diagnosis* (post post hoc analysis)**			
Sensitivity % (n/N, [95%CI])	26.1% (12/46 [14.3%, 41.1%])	15.8% (6/38 [6.0%, 31.3%])	23.8% (10/42 [12.1%, 39.5%])
Specificity % (n/N, [95%CI])	82.7% (393/475 [79.0%, 86.0%])	90.9% (432/475 [88.0% 93.4%])	91.5% (432/472 [88.6%, 93.9%])
Area under the receiver operating characteristic curve (95%CI)	0.54 (0.48, 0.61)	0.53 (0.47, 0.59)	0.58 (0.51, 0.64)
***Clinical tuberculosis includes: all mycobacteriology negative participants who were started on tuberculosis treatment based on abnormal chest radiograph, or other clinical criteria. CI = Confidence Interval.**			

Supplementary table 3. Description of the 12 participants who experienced Serious Adverse Events during the ACT-TB Trial

Participant	Treatment arm	Sex	Age	SAE Term	Received TB Treatment	Event Outcome
1	Amoxicillin	Male	24	Tuberculosis IRIS	Yes	Died
2	Amoxicillin	Male	51	Hepatitis B	No	Died
3	Standard of care	Female	28	Severe anaemia in pregnancy	No	Died
4	Standard of care	Female	51	Severe pneumonia	No	Died
5	Azithromycin	Male	30	Pleural effusion	Yes	Recovered
6	Amoxicillin	Male	24	Disseminated Tuberculosis and Empyema	Yes	Recovered
7	Azithromycin	Male	31	Disseminated Tuberculosis	Yes	Recovered
8	Azithromycin	Female	35	Hypertension in pregnancy	No	Recovered
9	Amoxicillin	Female	86	Congestive cardiac failure and hypertension	No	Recovered
10	Amoxicillin	Female	28	Parapneumonic abscess	No	Recovered
11	Standard of care	Male	36	Fibrotic lung disease	No	Recovered
12	Standard of care	Female	53	Diabetes Mellitus	No	Recovered

Randomised controlled clinical trial investigating benefits of using response to broad spectrum antibiotics as an exclusion diagnostic for tuberculosis (TB) in primary care adult patients versus risk of antimicrobial resistance (AMR)

Short title: **A**ccuracy and **C**onsequences of using **T**rial-of-antibiotics for **TB** diagnosis

Acronym: ACT-TB Study

Trial registration:

Protocol version: 4.0, 27 Jan 2020

Chief Investigator: Titus H Divala
London School of Hygiene & Tropical Medicine
Keppel Street, London WC1E 7HT
Tel: +44 740 523 1847
Email: titus.divala@lshtm.ac.uk

Co-Investigators: Katherine L Fielding, Neil French, Derek J Sloan, Elizabeth L Corbett

Collaborators: Marriott Nliwasa, Augustine Choko, Ankur Gupta-Wright, Jennifer Cornic, Jon Øyvind Odland, Chisomo Msefula, Hendramoorthy Maheswaran

Sponsor: London School of Hygiene & Tropical Medicine is the main research sponsor for this study. For further information regarding the sponsorship conditions, please contact the Research Governance and Integrity Office:
London School of Hygiene & Tropical Medicine
Keppel Street, London WC1E 7HT
Tel: +44 207 927 2626, Email: RGIO@lshtm.ac.uk

Funding: Helse Nord RHF
Tromsø, Norway; Tel: +47 97065144
Email: Hanne.Husom.Haukland@helse-nord.no

Study Coordination Centre: University of Malawi College of Medicine
Private Bag 360
Chichiri, Blantyre, Malawi
Tel: +2651871911
Email: rscdirector@medcol.mw

This trial will adhere to the principles outlined in the International Council for Harmonisation Good Clinical Practice (ICH GCP) guidelines, protocol and all applicable local regulations.

Approved by
College of Medicine
13-Feb-2020
(COMREC)
Research and Ethics Committee

Table of contents

1 Abbreviations 6

2 Trial Investigational Team 8

3 Executive Summary 10

3.1 Study type 10

3.2 Background 10

3.3 Problem statement 10

3.4 Objectives 10

3.4.1 Broad objective of the study 10

3.4.2 Specific objectives of the study 10

3.5 Methods 11

3.6 Expected results and dissemination 11

4 Tabular summary and schematic 12

5 Background information and introduction: 15

5.1 Background 15

5.2 Systematic literature review 17

5.3 Planned study 18

6 Rationale for the study 20

6.1.1 Accuracy of trial-of-antibiotics 20

6.1.2 Antimicrobial resistance and trial-of-antibiotics 20

6.1.3 Potential benefits of antibiotics 21

6.1.4 Important subgroups 22

6.2 Choice of study interventions 22

6.3 Nasopharyngeal pneumococcus for AMR 23

7 Objectives and outcomes 25

7.1 Broad objective of the study 25

7.2 Specific objective of the study 25

7.2.1 Primary .... **Error! Bookmark not defined.**

7.2.2 Secondary .... **Error! Bookmark not defined.**

8 Study design, participants, and statistical approach 27

Approved by
College of Medicine
13-Feb-2020
(COMREC)
Research and Ethics Committee

8.1 Study design 27

8.2 Study setting 27

8.3 Standard of care 27

8.4 Eligibility criteria 28

8.4.1 Inclusion Criteria 29

8.4.2 Exclusion Criteria 29

8.5 Interventions 29

8.5.1 Name and description of intervention arms 29

8.5.2 Legal status of drugs used in intervention arms 29

8.5.3 Summary of Product Characteristics 29

8.5.4 Drug Storage and Supply 29

8.5.5 Preparation and labelling of study drugs 30

8.5.6 Known drug reactions (adverse events) 30

8.5.7 Concomitant medication and interaction with other therapies 30

8.5.8 Trial restrictions 30

8.5.9 Assessment of compliance 30

8.5.10 Withdraw of interventions 30

8.6 Statistical approach 30

8.6.1 Primary outcome 31

8.6.2 Secondary outcomes 33

8.6.3 Exploratory outcome 34

8.6.4 Planned subgroup analyses 34

8.7 Sample size and power 34

8.7.1 Primary outcome1: specificity of trial-of-antibiotics versus mycobacteriology 34

8.7.2 Primary outcome 2: Incidence of adverse clinical outcome at Day-29 35

8.7.3 Secondary outcome 36

8.7.4 Exploratory outcomes 36

9 Pilot study 37

9.1 Specific objectives of the pilot study 37

9.2 Population for the pilot study 38

9.3 Pilot study procedures 38

9.4 Data analysis 39

10 Study procedures 41

10.1 Screening 41

10.2 Informed consent 41

Approved by
College of Medicine
13-Feb-2020
(COMREC)
Research and Ethics Committee

10.3 Baseline procedures 41

10.4 Assignment of interventions 42

10.5 Blinding 43

10.6 Participant follow up 44

10.6.1 *Day-8 activities* 44

10.6.2 *Day-29 activities* 45

10.7 Laboratory methods 45

10.7.1 *Tuberculosis mycobacteriology* 45

10.7.2 *Urine antigen testing for lipoarabamannan and other MTB antigens* 46

10.7.3 *Antimicrobial resistance testing* 46

10.8 Loss to follow-up 46

10.9 Trial closure 46

10.10 Summary schedule for study procedures 47

11 Safety reporting 49

11.1 Definitions 49

11.2 DMID grading for AEs 50

11.3 Grading for expected events 50

11.4 Causality 52

11.5 Reporting Procedures 52

11.5.1 *Non-serious Adverse Events (AEs)* 52

11.5.2 *Serious Adverse Events (SAEs)* 53

12 Economic evaluation 54

12.1 Objective 54

12.2 Outcomes 54

12.3 Data collection 54

12.4 Data analysis 55

12.5 Missing data 55

13 Data management 56

13.1 Source Data 56

13.2 Data collection methods 56

13.3 Data management 56

13.4 Quality control and quality assurance 57

Approved by
College of Medicine
13-Feb-2020
(COMREC)
Research and Ethics Committee

13.5 Access to data ... 57

14 Data monitoring and quality assurance ... 58

14.1 Data monitoring ... 58

14.2 Audits and Inspections ... 58

14.3 Data Safety and Monitoring Board (DSMB) ... 58

14.4 Trial Management Group (TMG) ... 58

15 Ethics and dissemination ... 59

15.1 Risk assessment ... 59

15.2 Research ethics approval ... 59

15.3 Indemnity ... 59

15.4 Sponsor ... 59

15.5 Declaration of interests ... 59

15.6 Cost of participation, ancillary and post-trial care ... 60

15.7 Dissemination policy ... 60

16 Study requirements, budget and justification ... 61

16.1 Budget justification ... 61

16.1.1 Study staff ... 61

16.1.2 Materials and consumables ... 62

16.1.3 Miscellaneous costs ... 62

17 References ... 63

18 Appendix 1: Informed consent ... 66

19 Appendix 2: Division of Microbiology and Infectious Diseases (DMID) adult toxicity table ... 67

20 Appendix 3: Package insert for Azithromycin and amoxicillin ... 79

Approved by
College of Medicine
13-Feb-2020
(COMREC)
Research and Ethics Committee

1 Abbreviations

ACASI	Audio Computer Assisted Self-Interview
AE	Adverse Event
AMR	Antimicrobial Resistance
AR	Adverse Reaction
ART	Antiretroviral Therapy
CD4	Cluster of Differentiation 4
CEACs	Cost-Effectiveness Acceptability Curves
COMREC	University of Malawi College of Medicine Research and Ethics Committee
CXR	Chest X-Ray
DMID	Division of Microbiology and Infectious Diseases
DSMB	Data Safety and Monitoring Board
GLM	Generalised Linear Model
HIV	Human Immunodeficiency Virus
HRQoL	Health Quality of Life
LAM	Urine Lipoarabinomannan Assay
LJ	Lowenstein-Jensen
LSHTM	London School of Hygiene & Tropical Medicine
MDA	Mass Drug Administration
MGIT	Mycobacteria Growth Indicator Tube
MTB or M.tb	*Mycobacterium tuberculosis*

Approved by
College of Medicine
13-Feb-2020
(COMREC)
Research and Ethics Committee

NMBs	Net Monetary Benefits
NTM	Non-Tuberculous Mycobacteria
NTP	National Tuberculosis Control Program
NTS	Non-Typhoidal Salmonellae
PCP	Pneumocystis Jiroveci
PLHIV	People Living With HIV
PTB	Pulmonary Tuberculosis
QALY	Quality-Adjusted Life Year
RCT	Randomized Controlled Trial
SAE	Serious Adverse Event
SAR	Serious Adverse Reaction
SOC	Standard of Care
STGG	Skim Milk Tryptone Glucose Glycerol
SUSAR	Suspected Unexpected Serious Adverse Reaction
TB	Tuberculosis
TMG	Trial Management Group
WHO	World Health Organization
WTP	Willingness to Pay

Approved by
College of Medicine
13-Feb-2020
(COMREC)
Research and Ethics Committee

2 Trial Investigational Team

Our investigational team includes expertise in diagnosis and management of TB; clinical evaluation of TB diagnostics; design and conduct of large randomised controlled trials; laboratory TB and AMR diagnostics; data management and analysis.

Chief investigator

Dr Titus H Divala: will be responsible for protocol development, coordination and conduct of the trial, governance, data management, data analysis and results dissemination. Dr Divala is a clinician with a career interest in clinical trials. Apart from medical training, he holds MPH and Masters of Science in Epidemiology and Preventive Medicine. In his career, Dr Divala has managed two large GCP, US-NIH-funded clinical trials, one of which was IND as the local PI supervising over 40 study staff at two sites in different cities; and coordinating protocol work across 4 laboratories in 3 cities. He has worked as a clinician for over 8 years in Malawi, a period when identifying TB cases and putting them on treatment was a daily job. This topic therefore falls in area of great personal interest above and beyond the potential benefit it has towards improving patient care in Malawi and all low and middle-income countries where 95% of the TB burden lies, where this approach is the standard.

This work will contribute towards PhD thesis for Dr Divala whose training is registered in the Infectious Disease Epidemiology Department of the London School of Hygiene & Tropical Medicine (LSHTM). The research is hosted in the Helse Nord Tuberculosis Initiative (HNTI), Department of Microbiology, University of Malawi College of Medicine where he is a Fellow. The research and his training are funded through a training grant from Helse Nord RHF of Norway which is managed by the Research Support Center (RSC).

Co-investigators and members of PhD supervisory team

Prof Katherine L Fielding: a seasoned TB statistician and clinical trialist, and Dr Divala's PhD supervisor for the CI, will be responsible for protocol development, conduct of the study, and data dissemination. Prof Fielding is also the Director of the LSHTM TB Center.

Prof Elizabeth L Corbett: a seasoned TB clinical epidemiologist and and Dr Divala's PhD co-supervisor for the CI, will be responsible for protocol development, conduct of the study, and data dissemination. Prof Corbett is a Wellcome Trust Senior Fellow.

Co-investigators and members of PhD advisory committee

Prof Neil French: a seasoned pneumococcal expert, will be responsible for protocol development, oversee all aspects of AMR work, and data dissemination.

Dr Derek J Sloan: clinician with detailed local clinical and research experience, will be responsible for protocol development, trial implementation and data dissemination.

Collaborators

Approved by
College of Medicine
13-Feb-2020
(COMREC)
Research and Ethics Committee

Dr Marriott Nliwasa: clinician, with experience conducting studies in the study setting. He will be support the conduct of the study, linkage with the national program, and data dissemination.

Mr Augustine Choko: statistician, with expertise and experience in using ACASI. He will support ACASI development, data management and development of analysis plan.

Dr Ankur Gupta-Wright: clinician, will provide clinical input in protocol development and clinical consultation support to research coordinators during study implementation..

Dr Jennifer Cornick: microbiologist, will be support protocol development, and AMR laboratory methods and analysis, and data dissemination.

Prof Jon Øyvind Odland: Epidemiologist and honorary professor at University of Malawi College of Medicine, responsible for seeking ethical approvals from the funder appointed ethics committee.

Approved by
College of Medicine
13-Feb-2020
(COMREC)
Research and Ethics Committee

3 Executive Summary

3.1 Study type

This proposal is for a randomised controlled clinical trial. It is individually randomised, open label and has three arms.

3.2 Background

Antimicrobial resistance (AMR) is a growing public health threat that is in part fuelled by empirical antibiotic usage. Empirical antibiotic use is often motivated by lack of point of care diagnostics a common problem in infectious diseases most of which are life-threatening. Tuberculosis (TB), the leading cause of infectious disease mortality, is one of the life-threatening illnesses without adequate diagnostics. Just over 50% of TB cases reported to WHO annually have confirmed mycobacteriological diagnosis. To complement the diagnostic gap, standard diagnostic algorithms include empirical antibiotic use. The antibiotic course, referred to as "trial-of-antibiotics", given to mycobacteriology-negative but symptomatic adults, is often broad-spectrum aiming to provide treatment for pneumonia. The goal is to treat infectious causes of respiratory symptoms other than TB, effectively performing the role of a "rule-out" diagnostic test for TB.

3.3 Problem statement

Approximately 26.5 million antibiotics courses are prescribed in the course of diagnosis of the 5.3 million smear negative TB registrations per annum. Despite this widespread use, there is no randomised controlled trial (RCT) evidence supporting the diagnostic accuracy of antibiotic trials and their impact on AMR. It is also unknown whether this usage of antibiotics can improve clinical outcomes considering that in settings of high HIV prevalence, bacterial infection associated mortality just before and during TB treatment is high.

3.4 Objectives

3.4.1 Broad objective of the study

To determine the benefits and consequences (antimicrobial resistance) of using trial-of-antibiotics in TB diagnostic algorithms in low and middle income countries.

3.4.2 Specific objectives of the study

3.4.2.1 Primary

- To establish the diagnostic value of trial-of-antibiotics for excluding pulmonary tuberculosis (PTB) in adults with cough (and have a valid sputum test result) at primary care level in Malawi.
- To determine the overall clinical benefit of giving empirical antibiotic treatment in primary care participants with cough.

3.4.2.2 Secondary

Approved by
College of Medicine
13-Feb-2020
(COMREC)
Research and Ethics Committee

- To evaluate using nasopharyngeal Streptococcus pneumonia, the effect of a trial-of-antibiotics on selection for antimicrobial resistance.
- To establish the diagnostic value of trial-of-antibiotics for excluding pulmonary tuberculosis (PTB) in primary care presenting Malawian adults with prolonged cough including those without a successful sputum test (unable to submit sputum and those with invalid sputum results)
- To estimate the incremental cost-effectiveness of trial-of-antibiotics using azithromycin and trial-of-antibiotics using amoxicillin in comparison to standard of care, and to each other.

3.5 Methods

To address the evidence gaps related to a) accuracy, b) antimicrobial resistance, and c) impact on clinical outcomes), we propose to conduct a randomised controlled clinical trial recruiting adult patients presenting to primary care centres in Blantyre, Malawi with history of cough for at least 2 weeks. After excluding those with danger signs we will randomise participants to receiving or not receiving trial-of-antibiotics (azithromycin or amoxicillin) from Day-1 to determine diagnostic accuracy (specificity) against mycobacteriology reference standard (smear microscopy, Xpert/MTB/RIF and culture). Our second primary outcome will be the between-arms difference of incidence of either death or hospitalisation or missed TB diagnosis by Day 29.

For secondary outcomes, we will compare between arms differences in incidence of antimicrobial resistance and cost-effectiveness by Day-29. To our knowledge this will be the first randomised controlled trial to address these questions in over 20 years of systematic use of trial-of-antibiotics without strong evidence base.

To adequately address the primary objective, we will need 625 participants in each of the three arms (azithromycin, amoxicillin and standard of care), a total sample size of 1875 participants.

3.6 Expected results and dissemination

The detailed understanding of the value of a trial-of-antibiotics in the context of diagnosis of TB will have invaluable impact on patient care in Malawi and the rest of the low and middle income world which hosts 95% of the global TB burden. This work will form part of a PhD thesis for Titus Divala, which he will submit to the London School of Hygiene & Tropical Medicine (LSHTM). We will share the results of this work with COMREC, LSHTM REC and Regional Committee for Health and Research Ethics at NTNU, Norway. The Malawi National TB Control Program are already aware of the study through our long standing collaborations. Apart from NTP, we will share our results with Blantyre District Health Office, the wider Ministry of Health, and the University of Malawi College of Medicine via the annual research dissemination conference. We will also prepare manuscripts for peer reviewed publications.

Approved by
College of Medicine
13-Feb-2020
(COMREC)
Research and Ethics Committee

4 Tabular summary and schematic

**Title**	Randomised controlled clinical trial investigating benefits of using response to broad spectrum antibiotics as an exclusion diagnostic for tuberculosis (TB) in primary care adult patients versus risk of antimicrobial resistance (AMR)	
**Design**	Three arm (625 per arm) individually randomised (1:1:1), open-label controlled clinical trial investigating standard care diagnostic approach for tuberculosis. The trial will not use any unlicensed products.	

**Objective**	**Outcomes**
**Primary**	
1. To establish the diagnostic value of trial-of-antibiotics for excluding pulmonary tuberculosis (PTB) in adults with cough (and have a valid sputum test result) at primary care level in Malawi.	Proportion of participants correctly classified as PTB negative based on report of improvement of baseline symptoms on study Day-8 (i.e. after a trial-of-antibiotics if in azithromycin or amoxicillin arms, or without antibiotics if in standard of care arm) against a mycobacteriology reference standard, among participants with a valid result from at least one sputum TB test
2. To determine the overall clinical benefit of giving empirical antibiotic treatment in primary care participants with chronic cough.	Proportion of participants experiencing at least one of the following adverse outcomes by Day 29:<br>1) death<br>2) hospitalisation<br>3) missed TB diagnosis
**Secondary**	
3 To evaluate using nasopharyngeal Streptococcus pneumonia, the effect of a trial-of-antibiotics on selection for antimicrobial resistance.	Proportion of day 29 nasopharyngeal *Streptococcus pneumoniae* isolates resistant to commonly used antimicrobials.
4. To establish the diagnostic value of trial-of-antibiotics for excluding pulmonary tuberculosis (PTB) in primary care presenting Malawian adults with cough including those without a successful sputum test (unable to submit sputum and those with invalid sputum results).	Proportion of participants correctly classified as PTB negative based on report of improvement of baseline symptoms on study Day-8 (i.e. after a trial-of-antibiotics if in azithromycin or amoxicillin arms, or without antibiotics if in standard of care arm) against a mycobacteriology reference standard, among all randomised participants, with those who could not provide sputum or had an invalid sputum result classified as mycobacteriologically negative.

Approved by
College of Medicine
13-Feb-2020
(COMREC)
Research and Ethics Committee

<table>
<tr><td colspan="2">5. To estimate the incremental cost-effectiveness of trial-of-antibiotics using azithromycin and trial-of-antibiotics using amoxicillin in comparison to standard of care, and to each other.</td><td>Incremental cost per quality adjusted life year gained
Total direct medical costs per participant over 56 days
Eq-5D utility score</td></tr>
<tr><td colspan="3">Exploratory</td></tr>
<tr><td colspan="3">Our exploratory analyses will be comparisons between the azithromycin and amoxicillin arms for all our primary and secondary outcomes.</td></tr>
<tr><td>Population</td><td colspan="2">Adults presenting to primary care centres in Malawi reporting cough.</td></tr>
<tr><td></td><td colspan="2">Inclusion criteria:
Ambulatory clinic attendees presenting with cough
Should have been ill for ≥ 14 days
Aged at least 18 years
Reside in Blantyre and willing to return to the same clinic for follow up visits over the entire study period.
Exclusion criteria:
Self-reported allergy to study medications
Acute danger signs defined in national TB treatment guidelines
Tuberculosis treatment or isoniazid preventive therapy in the last 6 months
Treated with antibiotics, other than co-trimoxazole prophylaxis, for the current illness or within the past 14 days</td></tr>
<tr><td>Treatment</td><td colspan="2">Arm 1: Azithromycin 500mg once daily for 3 days commencing on randomization day.
Arm 2: Amoxicillin 1 g 3 times daily for 5 days commencing on randomization day.
Arm 3: Standard of care in current national guidelines for patients presenting with cough and without danger signs (No treatment until re-evaluation with with sputum TB test results)</td></tr>
<tr><td>Duration</td><td colspan="2">We will give treatments on the randomisation day (Day-1) and perform follow up activities on days 8, and 29.</td></tr>
</table>

Approved by
College of Medicine
13-Feb-2020
(COMREC)
Research and Ethics Committee

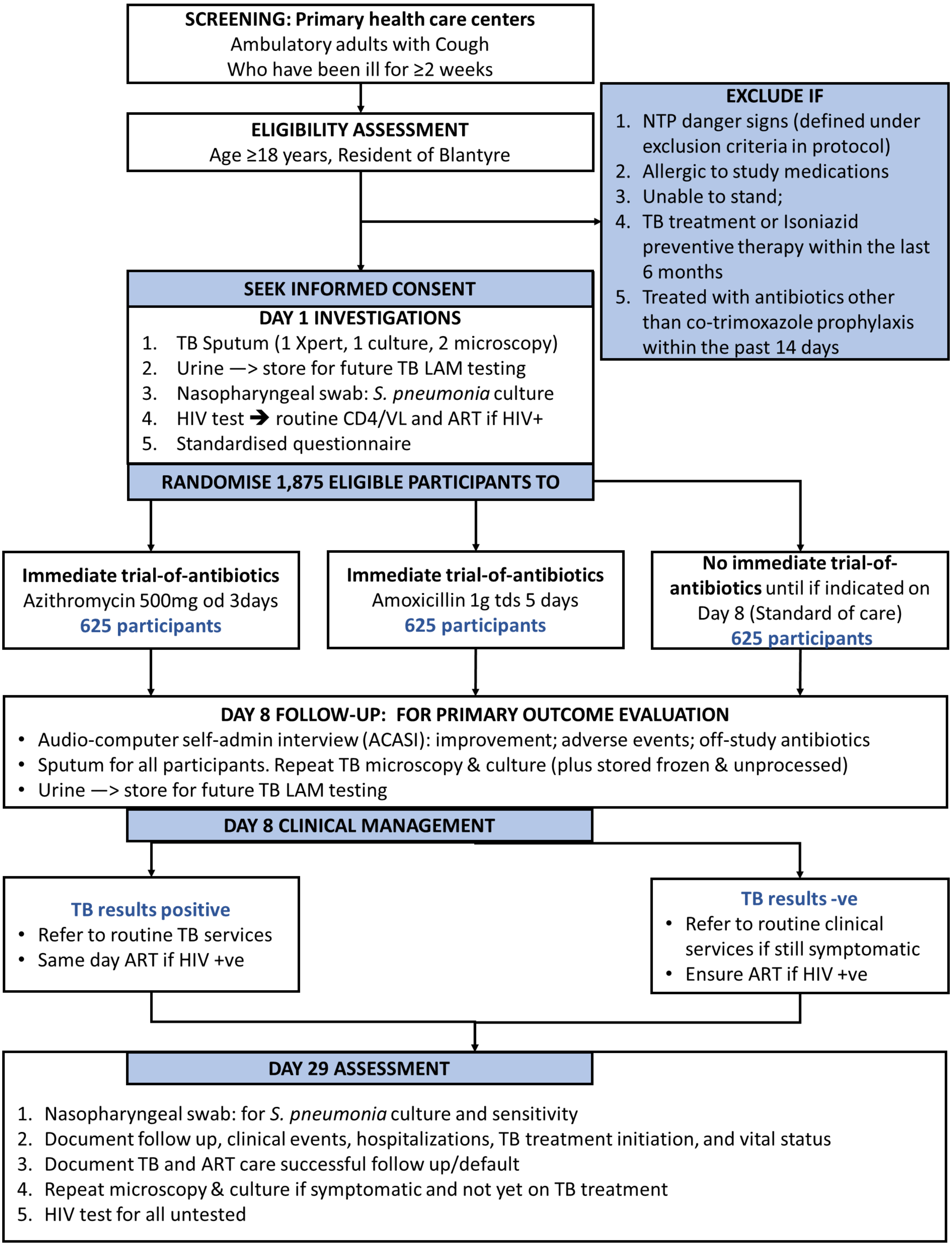

Figure 1: Flow diagram for the planned clinical trial in Blantyre, Malawi

Approved by
College of Medicine
13-Feb-2020
(COMREC)
Research and Ethics Committee

5 Background information and introduction:

5.1 Background

Antimicrobial resistance is a growing crisis, becoming in 2016 one of only four health topics ever to be discussed at the United Nations General Assembly.[1-4] Tuberculosis is the leading global infectious cause of death in adults,[5] with approximately 10.4 million cases and 1.8 million deaths in 2015.[6] The high case-fatality rate in part reflects suboptimal diagnostics(Figure 2).[7-10]

Diagnosis method	Percentage
Mycobacteriology	57%
Clinical	43%

A. WHO annual TB notifications (WHO 2017 TB Report)

B.

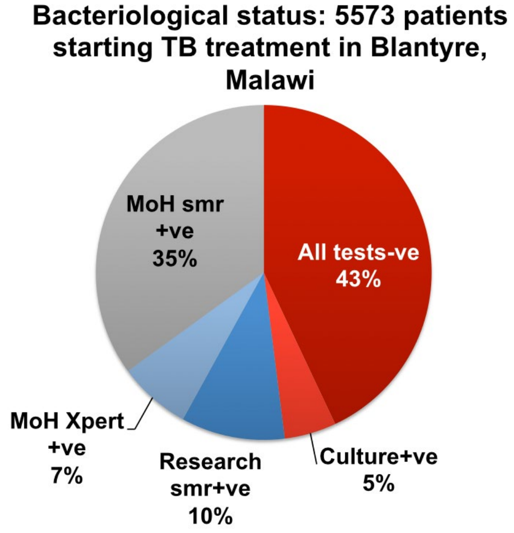

Nliwasa et.al

Figure 2: method of diagnosis for TB notifications globally (A) and in Blantyre, Malawi (B)

To complement the suboptimal diagnostics, standard diagnostic algorithms in resource-limited settings include a "trial-of-antibiotics" (Figure 3). This is a course of broad-spectrum antibiotics, with negligible *Mycobacterium tuberculosis* activity, given to patients with symptoms such as cough in order to "rule-out" or "rule in" tuberculosis.[11-13] Patients with negative sputum mycobacteriology and responded to antibiotic treatment are considered tuberculosis negative while those who remain symptomatic are deemed likely to have tuberculosis and undergo further evaluations leading on to receiving tuberculosis treatment.

Approved by College of Medicine 13-Feb-2020 (COMREC) Research and Ethics Committee

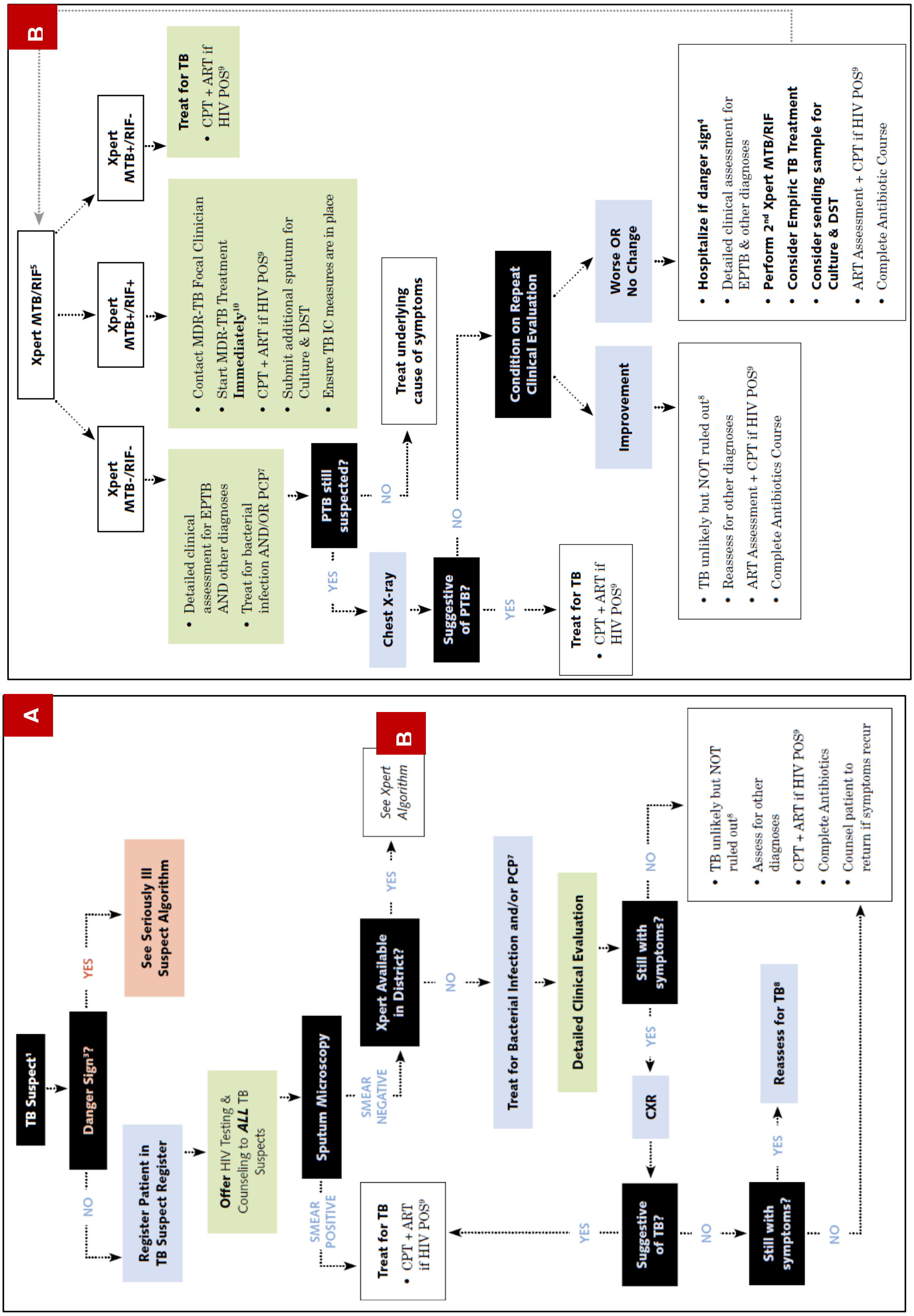

Figure 3: Implementation of trial-of-antibiotics (marked with red boxes) in Malawi TB diagnostic algorithm, National TB control program (NTP)

Approximately 26.5 million course of antibiotics are prescribed in the diagnosis of the 5.3 million smear negative tuberculosis registrations per annum (Figure 4).[6] This estimate is based on an average of 5 antibiotic courses per sputum-negative treatment initiation, with 2 courses given to the patients before

Approved by
College of Medicine
13-Feb-2020
(COMREC)
Research and Ethics Committee

tuberculosis treatment,[8] and the other 3 courses accounting for patients whose symptoms resolved and tuberculosis was ruled out.[14].

Wilkinson et al[14] prescribed 120 + 74 courses of trial-of-antibiotics to diagnose 40 smear-negative TB patients (a typical ratio of ~1:5).[8] If generalizable, then for 5.3 million annual smear-negative TB registrations globally ~5 x 5.3 million trial-of-antibiotics courses (26.5 million) will have been prescribed.

**Enrolled**	**280**	
TB smear microscopy positive		160
Given trial-of-antibiotics (amoxicillin)	**120**	
Improved, declared TB negative		46
Given trial-of-antibiotics (erythromycin)	**74**	
Improved, declared TB negative		34
Treated for smear negative TB	**40**	

Wilkinson et.al Int J Tuberc Lung Dis. 2000

Figure 4: Quantifying number of trial-of-antibiotics courses prescribed per year using data from Wilkinson et.al and WHO TB Report 2016

Despite this widespread use, there is no randomised controlled trial evidence supporting the diagnostic accuracy of trial-of-antibiotics. There is also a dearth of evidence on their impact on antimicrobial resistance or patient clinical outcomes.

5.2 Systematic literature review

We performed a systematic literature review to determine the sensitivity and specificity of using a trial-of-antibiotics compared to sputum mycobacteriology for diagnosis of PTB. We also wanted to describe how trial-of-antibiotics tits into TB diagnostic algorithms: timing of prescription; type, duration, and number of antibiotic prescriptions; and how response to treatment is measured. We searched MEDLINE, Embase, and Global Health using the Ovid platform to identify studies meeting the following criteria:

Population	Adult patients with symptoms suggestive of pulmonary tuberculosis
Intervention	Routinely prescribed broad-spectrum oral antibiotics without MTB activity, and given as part of evaluation of pulmonary TB
Outcome	sensitivity and specificity of the intervention in comparison with any mycobacteriology test
Study design	Any design with prospective component allows evaluation of the outcome of the intervention
Time frame	Studies published after WHO declaration of TB as a 'global emergency' (1993)
Language	English, lack of translation capacity

Approved by
College of Medicine
13-Feb-2020
(COMREC)
Research and Ethics Committee

We identified 7,064 articles from a systematic search on MEDLINE, Embase, and Global Health using the Ovid platform. Of these studies, 12 were eligible for narrative synthesis and seven had suitable data for meta-analysis. None of the studies was an RCT and all the observational studies were small and not primarily designed to address the benefits and consequences of trial-of-antibiotics. Unlike our proposed RCT, most of the published work was from hospital setting or in specialised clinics. Most studies used amoxicillin and some studies prescribed a subsequent course of antimicrobials either before or after assessing for improvement. The definition of improvement from baseline clinical state was largely subjective: it was based on self-report, clinical examination, radiological assessment or a combination.

There is no consensus on the sensitivity and specificity of trial-of-antibiotics across studies with estimates ranging from 43% to 91% for sensitivity and 41% to 82% for specificity (shown below).

Population, Study		Sensitivity, 95% CI			Specificity, 95% CI		
237	Wilkinson et al 1997	0.5	0.37	0.64	0.82	0.76	0.87
120	Wilkinson et al 2000	0.83	0.71	0.91	0.56	0.44	0.67
204	Kudjawu et al 2006	0.91	0.83	0.96	0.65	0.56	0.73
1000	Kamran et al 2006	0.72	0.62	0.8	0.41	0.38	0.44
264	Soto et al 2011	0.43	0.32	0.55	0.68	0.63	0.72
439	Soto et al 2013	0.46	0.34	0.58	0.6	0.53	0.66
440	Padmapriyadarsini et al 2	0.7	0.55	0.8	0.69	0.64	0.73

We could not identify any RCT, the current literature only has small studies, with trial-of-antibiotics not being the primary focus of investigation in most cases. There is limited data for primary care settings as most of the work was in hospital setting. None of the studies addressed AMR. Therefore, despite widespread use, the approach, the value and consequences of having trial-of-antibiotics in TB diagnostic algorithms, remains to be established.

5.3 Planned study

To address the evidence gaps related to a) accuracy, b) antimicrobial resistance, and c) impact on clinical outcomes), we propose to conduct a randomised controlled clinical trial recruiting adult patients presenting to primary care centres in Blantyre, Malawi with history of cough for. After excluding those with danger signs we will randomise participants to receiving or not receiving trial-of-antibiotics (azithromycin or amoxicillin) from Day-1 to determine diagnostic accuracy (specificity) against mycobacteriology reference standard (smear microscopy, Xpert/MTB/RIF and culture).

Approved by
College of Medicine
13-Feb-2020
(COMREC)
Research and Ethics Committee

For secondary outcomes, we will also compare between arms differences in antimicrobial resistance and clinical outcomes (risk of death, hospitalisation, and missed TB diagnosis) at Day-29. To our knowledge this will be the first randomised controlled trial to address these questions in over 20 years of systematic use of trial-of-antibiotics without strong evidence base.

Approved by
College of Medicine
13-Feb-2020
(COMREC)
Research and Ethics Committee

6 Rationale for the study

6.1.1 Accuracy of trial-of-antibiotics

As an approach that is being used on such a large scale, trial-of-antibiotics should ideally have a strong evidence-base (supported by reference mycobacteriology) of how much diagnostic and/or clinical improvement it brings to the TB diagnostic algorithm.[15,16] This will be among the most important considerations when deciding whether it is worth the trade-off with potential for AMR. Such evidence could come from an RCT or a well-designed prospective study.[15-17] However, despite being in use for more than 20 years, we have not identified any such clinical trial, and even the observational evidence is highly limited and of insufficient quality and quantity to definitively address the question.

There is also no guidance on antibiotic choice beyond a recommendation to avoid those with anti-tuberculosis activity (like fluoroquinolones). Another key area that lacks clarity is lack of a clear definition for clinical resolution when determining the outcome of trial-of-antibiotics. Clinical resolution is the basis for decisions that follow (i.e. discontinue follow up or proceed Antimicrobial resistance and trial-of-antibiotics

Antimicrobial resistance can be either intrinsic or acquired. The risk of acquired resistance relating to antibiotic use during evaluation for suspected tuberculosis has not been previously investigated, although previous work has shown that empirical antibiotics can drive rapid emergence of AMR.[18,19] For example, co-trimoxazole prophylaxis for HIV-positive patients, introduced in 2005, was followed by near-universal resistance in bloodstream infections by 2010.[20] Mass drug administration of azithromycin for trachoma control initially reduces nasopharyngeal carriage of *Streptococcus pneumoniae*, but with increased macrolide-resistance 6 months later.[21,22]

In our study, the AMR risks of empirical antibiotic prescriptions (azithromycin and amoxicillin arms of the RCT) are justified because of the widespread use of this approach for amoxicillin, and the low potential clinical impact and short-lived effects of use of azithromycin on AMR, given the limited use of macrolides in Malawi. Mathematical modelling work suggests that macrolide resistance can successfully be eliminated by intra-species competition alone (fitness cost) within 5 years of last use.[23]

6.1.2 Antimicrobial resistance and trial-of-antibiotics

Antimicrobial resistance relating to antibiotic use during evaluation for suspected tuberculosis has not been investigated before. Previous work has shown that empirical antibiotics can drive rapid emergence of antimicrobial resistance.[18,19] Co-trimoxazole prophylaxis for HIV-positive patients, introduced in 2005, was followed by near-universal resistance in bloodstream infections by 2010[20] also shown in Table 1. Mass drug administration of azithromycin for trachoma control initially reduces nasopharyngeal carriage of *Streptococcus pneumoniae*, but with increased macrolide-resistance 6 months later.[21,22]

We will investigate antimicrobial resistance in nasopharyngeal *S. pneumonia* by randomisation arm and cumulative antibiotic exposure to assess the extent to which brief exposure drives antimicrobial resistance during diagnostic work-up for tuberculosis. An ecological niche for many bacterial species,

Approved by
College of Medicine
13-Feb-2020
(COMREC)
Research and Ethics Committee

the upper respiratory tract also presents a convenient window for investigating antimicrobial resistance.[24] *S. pneumonia* is the organism of choice not only for being an important cause of respiratory tract infections but also because it often colonises the upper respiratory tract and has well documented laboratory investigation procedures in place.[25] As exploratory analyses, we will also assess nasopharygeal colonization and antimicrobial resistance in relation to tuberculosis treatment and HIV status.

Table 1 Resistance patterns of common aetiologies of pneumonia to commonly used antimicrobials in Blantyre, Malawi

		Gram positive		Gram negative			
**Organism**		Streptococcus pneumoniae	Staphylococcus aureus	Haemophilus influenzae	Klebsiella pneumoniae	Escherichia coli	Pseudomonas aeruginosa
**Prevalence**		15.6%	6.6%	0.9%	4.4%	0.1%	1.5%
**Resistance percentage**	amoxicillin	*	*	58%	100%	94%	100%
	penicillin	21%	*	*	*	*	*
	co-trimoxazole	98%	40%	100%	92%	94%	75%
	chloramphenicol	21%	2%	92%	48%	61%	100%
	Erythromycin	2%	30%	*	*	*	*
	tetracycline	38%	35%	*	*	*	*
	Ceftriaxone	*	*	0%	90%	30%	100%
	Ciprofloxacin	*	*	NA	705	31%	24%

*not routinely tested
2016 data from hospitalised febrile patients at Queen Elizabeth central hospital (Blantyre, Malawi) as reported by the MLW Clinical research laboratory (unpublished).

6.1.3 Potential benefits of antibiotics

In areas with high HIV prevalence, empirical antibiotics during tuberculosis investigations could be life-saving: mortality immediately before and after tuberculosis diagnosis is high, [7,26] and is often secondary to severe bacterial infections.[26-28] The leading aetiologies of infection and death on tuberculosis treatment as well as among outpatients with tuberculosis-like symptoms are *Streptococcus pneumoniae* and non-typhoidal salmonellae (NTS): both can present with cough (primary cause) or as co-morbidities (super-infections) in patients presenting with active *Mycobacterium tuberculosis* (*M.tb*) disease.[26-28] If effective treatment of this type of life-threatening primary/super-infections reduces mortality during the diagnostic work-up of suspected TB in people living with HIV (PLHIV), then empirical use of broad-spectrum antibiotics would be indicated for this purpose alone, irrespective of any diagnostic contribution to TB treatment decisions. In this context, azithromycin may be the most effective arm, as Salmonella infections are highly sensitive to azithromycin, but not to amoxicillin.[29]

6.1.3.1 Measures of clinical benefit of trial-of-antibiotics

In this study, we will investigate the overall clinical benefit of trial-of-antibiotics by comparing the risk of any of death, hospitalisation, or missed TB diagnosis by Day 29. Although all these events are potential consequences of trial-of-antibiotics, grouping them as a single composite endpoint may

Approved by
College of Medicine
13-Feb-2020
(COMREC)
Research and Ethics Committee

only appropriately represent the effect of the intervention 1) there are similarities in the importance patients would attach to each of its components and 2) the components occur with similar frequencies in the patient population.[30]

The impact of antibiotics on hospitalisation and mortality causing illnesses is as described above. Both these outcomes are important with their similarity hinged on the fact that hospitalisation event predicts mortality. In patients with chronic cough, frequencies of mortality and that of hospitalization over a two months period are similar, ranging from 2 to 6%.[31]

TB misdiagnosis becomes a concern because of the potential for misclassification in either direction –false positive or false negative. False positive diagnosis in the context of trial-of-antibiotics would occur when the underlying pathology for the respiratory symptoms is not responding to the antibiotic, which can be secondary to either AMR or the illness not being of bacterial origin. On the other hand, patients would be prone to a false negative result had both TB and a susceptible bacterial infection. If the symptoms were largely driven by the susceptible bacterial infection, their symptoms will improve and would be declared TB negative. TB is a life-threatening illness, missing its diagnosis can therefore lead to death which is more important to an individual patient than taking TB chemotherapy with a false positive TB diagnosis. We will therefore include only missed TB diagnoses in the composite clinical outcome. Unpublished data from Blantyre shows that the frequency of missed TB diagnosis under routine care settings is approximately 5% which is similar to that of death and hospitalisation.

6.1.4 Important subgroups

Response to trial-of-antibiotic- in patients with bacteriologically confirmed tuberculosis (i.e. false-negatives/low sensitivity from the perspective of TB diagnosis) may relate to multiple super-infections and so this phenomenon may vary by HIV status, since multiple concurrent infections are a hallmark of advanced HIV immunosuppression, and commonly identified in patients with suspected TB in the pre-ART era.[8,27] More recently, in Malawi, 45% of adults who presented to primary care with prolonged cough (≥2 weeks) were HIV-positive, of whom only ~20% started TB treatment on the basis of positive mycobacteriology.[30] As such, the benefits and consequences of trial-of-antibiotics may vary by HIV status and by subsequent TB treatment decisions. We will, therefore, include a pre-specified sub-analysis of trial outcomes stratified by HIV and ART status.

6.2 Choice of study interventions

Our trial will compare azithromycin and amoxicillin to standard of care. We propose 2 different antibiotic arms for the following reasons: -

a) Macrolides, including azithromycin, are rarely used in Malawi because of their higher manufacturing costs. However, they do provide a more effective treatment of community-acquired pneumonia than the standard antibiotic by Ministry of Health for trial-of-antibiotic (amoxicillin), because of low levels of acquired macrolide-resistance in bacterial isolates in Malawi,[29] reflecting

Approved by
College of Medicine
13-Feb-2020
(COMREC)
Research and Ethics Committee

low rates of past exposure to this class of drugs, and also better intrinsic coverage of "bacterial cause of pneumonia including "atypical" intracellular organisms such as *mycoplasma* species.

Although viral pneumonias, *Pneumocystis jiroveci* (PCP) and non-infectious causes of cough will still not be expected to respond to azithromycin, this arm should then provide the highest possible diagnostic discrimination for bacterial vs mycobacterial causes of cough. The starting point of low pre-existing (acquired) resistance will also facilitate investigation of AMR acquired during trial-of-antibiotics. However, the trial will have limited national relevance in Malawi without comparison to an antibiotic in programmatic use.

b) Amoxicillin is low cost option that is still a recommended treatment for community-acquired pneumonia in most settings, including UK, despite potential treatment failure from bacterial pneumonia due to organisms with intrinsic ("atypicals") or acquired (common in gram-negative organisms, and *Staphylococcus aureus*) penicillin resistance.[29] This arm reflects the true standard of care (SOC) currently in widespread use in Malawi and many other low-income countries, and so provides data of immediate programmatic relevance and also a starting point to investigate exacerbation of pre-existing AMR pressure. If there is a marked difference between the azithromycin and amoxicillin arms, then there will also be important health economic considerations of relevance to many national TB programmes beyond Malawi.

Azithromycin provides effective treatment for community-acquired pneumonia[31-33] and has negligible activity against *M.tb.*[34,35] As discussed above, macrolides are not commonly used in Malawi. Azithromycin has an excellent safety profile and is used for mass drug administration (MDA) in communities prone to trachoma. Azithromycin used for MDA in Ethiopia reduced inter-current infections[21,36] and death in children,[37,38] supporting the safety of using this drug for our trial.[7]

Amoxicillin is the first line treatment for outpatient management of pneumonia in Malawi and is commonly used for trial-of-antibiotics. We anticipate higher specificity for azithromycin than amoxicillin, due to broader coverage of "atypical pneumonia" organisms, and salmonella species, but with the 2 antibiotics arms having "equipoise" due to lack of previous head-to-head comparison.[27]

6.3 Nasopharyngeal pneumococcus for AMR

Streptococcus pneumonia is a major cause of morbidity and mortality in children and adults.[20,29,39,40] Asymptomatic nasopharyngeal carriage of *S. pneumoniae* is common and a prerequisite for the occurrence and transmission of invasive pneumococcal disease.[41,42] Since carriage is more common than the invasive *S. pneumoniae* disease it forms a basis for establishing circulating serotypes, resistance patterns, and evaluation of vaccine effectiveness.

The other key advantage is the existence of globally accepted laboratory procedures for assessing and interpreting pneumococcal resistance. Our laboratory (in Malawi-Liverpool Wellcome Trust) has carried out pneumococcal work for decades with outstanding quality assurance reputation.

Approved by
College of Medicine
13-Feb-2020
(COMREC)
Research and Ethics Committee

Approved by
College of Medicine
13-Feb-2020
(COMREC)
Research and Ethics Committee

7 Objectives and outcomes

7.1 Broad objective of the study

To determine the benefits and consequences (antimicrobial resistance) of using trial-of-antibiotics in TB diagnostic algorithms in low and middle income countries.

7.2 Specific objective of the study

In Table 2 below, we present study objectives together with corresponding outcomes. We have clarified the outcomes with detailed definitions and planned analyses under "statistical approach" section.

Table 2: study objectives and outcomes

Objective	Outcome
**Primary**	
1. To establish the diagnostic value of trial-of-antibiotics for excluding pulmonary tuberculosis (PTB) in adults with cough (and have a valid sputum test result) at primary care level in Malawi.	Proportion of participants correctly classified as PTB negative based on report of improvement of baseline symptoms on study Day-8 (i.e. after a trial-of-antibiotics if in azithromycin or amoxicillin arms, or without antibiotics if in standard of care arm) against a mycobacteriology reference standard, among participants with a valid result from at least one sputum specimen
2. To determine the overall clinical benefit of giving empirical antibiotic treatment in primary care participants with cough.	Proportion of participants experiencing at least one of the following adverse outcomes by Day 29:<br>1) death<br>2) hospitalisation<br>3) missed TB diagnosis
**Secondary**	
3. To evaluate using nasopharyngeal Streptococcus pneumonia, the effect of a trial-of-antibiotics on selection for antimicrobial resistance.	Proportion of Day 29 acquiring nasopharyngeal *Streptococcus pneumoniae* isolates resistant to any of the commonly used groups of antimicrobials by Day-29.
4. To establish the diagnostic value of trial-of-antibiotics for excluding pulmonary tuberculosis (PTB) in primary care presenting Malawian adults with including those without a	Proportion of participants correctly classified as PTB negative based on report of improvement of baseline symptoms on study Day-8 (i.e. after a trial-of-antibiotics if in azithromycin or amoxicillin arms, or without antibiotics if in standard of care arm) against a

Approved by
College of Medicine
13-Feb-2020
(COMREC)
Research and Ethics Committee

successful sputum test (unable to submit sputum and those with invalid sputum results).	mycobacteriology reference standard, among all randomised participants, with those who could not provide sputum classified as mycobacteriologically negative.
5. To estimate the incremental cost-effectiveness of trial-of-antibiotics using azithromycin and trial-of-antibiotics using amoxicillin in comparison to standard of care, and to each other.	• Incremental cost per quality adjusted life year gained<br>• Total direct medical costs per participant over 56 days<br>• Eq-5D utility score
**Exploratory**	
Our exploratory analyses will be comparisons between the azithromycin and amoxicillin arms for all our primary and secondary outcomes.	

Approved by
College of Medicine
13-Feb-2020
(COMREC)
Research and Ethics Committee

8 Study design, participants, and statistical approach

8.1 Study design

This is a three arm (625 per arm) individually randomised (1:1:1), open-label controlled clinical trial investigating accuracy and broader clinical, and antimicrobial resistance impact of using trial-of-antibiotics to “rule out” tuberculosis among adults presenting with cough at primary care centres in Malawi.

8.2 Study setting

We will screen adults aged at least 18 presenting to primary care centres in Blantyre, Malawi. Blantyre has an estimated adult HIV prevalence of 12.7% (95% CI: 11.9 to 13.6) and an estimated tuberculosis prevalence of 1,014 per 100,000 (95% CI: 486 to 1,542).[43]

8.3 Standard of care

The standard of care in national guidelines from the NTP for primary care patients presenting with cough and are otherwise well (no danger signs) is to take sputum x 2 for smear microscopy or Xpert and ask them to return for results, typically 3 days - 1 week later (Figure 3 and 5). The Malawi tuberculosis diagnostic algorithm recommends use of broad-spectrum antibiotics as trial-of-antibiotics after negative sputum tests are provided to the patient, if they remain symptomatic.

However, more commonly this algorithm is adapted in the outpatient setting to combine prescription of antibiotics (usually amoxicillin) with sputum collection at the first visit, to save the patient from making separate visits: thus, our amoxicillin arm is the most common standard-of-care in Malawi, while the no-antibiotic arm is the NTP recommended standard-of-care.

Approved by
College of Medicine
13-Feb-2020
(COMREC)
Research and Ethics Committee

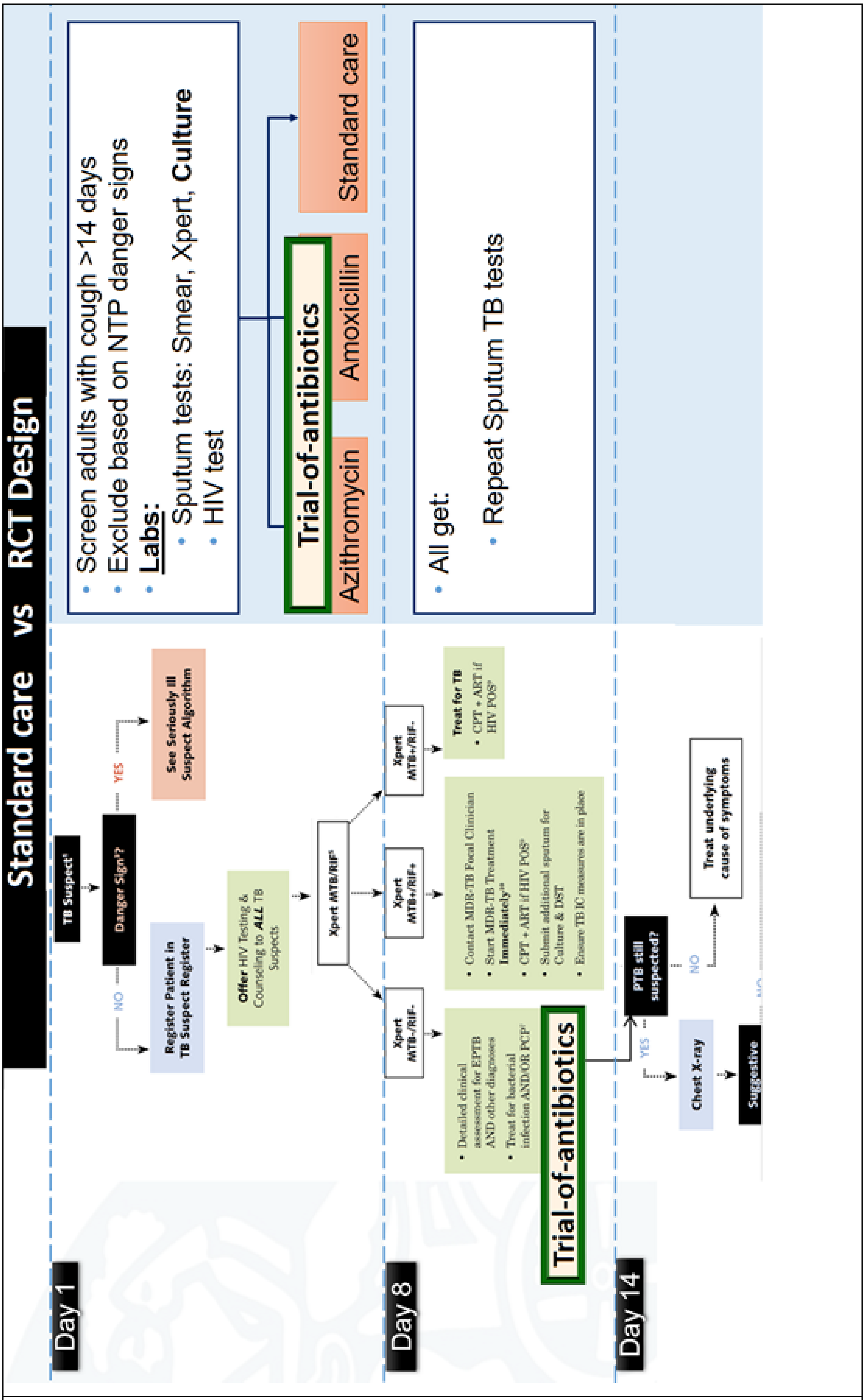

Figure 5: A comparison of standard of care (Malawi NTP guidelines) and the RCT design with respect to timing of trial-of-antibiotics and study procedures

8.4 Eligibility criteria

We will offer enrolment to patients who satisfy the following inclusion and exclusion criteria.

Approved by College of Medicine 13-Feb-2020 (COMREC) Research and Ethics Committee

8.4.1 Inclusion Criteria

- Ambulatory clinic attendees presenting with cough
- Should have been ill for at least 14 days
- Aged at least 18 years
- Reside in Blantyre and willing to return to the same clinic for follow up visits over the entire study period.

8.4.2 Exclusion Criteria

- Self-reported allergy to study medications
- Danger signs (WHO/Malawi NTP): respiratory rate > 30/min, temperature >39°C, Heart rate >120/minute, confused/agitated, respiratory distress, systolic blood pressure <90 mmHg, inability to walk unassisted
- Treated with antibiotics other than co-trimoxazole prophylaxis within the past 14 days
- TB treatment or Isoniazid preventive therapy within the last 6 months

8.5 Interventions

We will have two active study arms receiving trial-of-antibiotics at enrolment (azithromycin and amoxicillin) and a standard of care arm of no trial-of-antibiotics. In this study, the goal is to investigate the role of these antibiotics as they are used in TB diagnostic algorithms, as "trial-of-antibiotics," to exclude TB in symptomatic patients. The study is likely to be underpowered to detect differences between the 2 antibiotic arms will only be compared for exploratory outcomes.

8.5.1 Name and description of intervention arms

The study will have three arms as follows:

- Arm 1: Immediate trial-of-antibiotics with Azithromycin 500mg once daily for 3 days.
- Arm 2: Immediate trial-of-antibiotics with Amoxicillin 500 mg 3 times daily for 5 days.
- Arm 3: Standard of care

8.5.2 Legal status of drugs used in intervention arms

Both azithromycin and amoxicillin are registered for use in Malawi and United Kingdom, with both Arms 1 and 2 regimens being UK-recommended community-acquired pneumonia treatment.

8.5.3 Summary of Product Characteristics

Appendix 3 includes current versions of package insets for azithromycin and amoxicillin. We will review and update (when applicable) the package inserts annually with each ethics continuing review.

8.5.4 Drug Storage and Supply

Approved by
College of Medicine
13-Feb-2020
(COMREC)
Research and Ethics Committee

We will procure study products from Durbin PLC (DURBIN PLC 180 Northolt Road South Harrow Middlesex HA2 0LT). Azithromycin will be manufactured by Sandoz limited or other pharmaceutical companies recognised in United Kingdom where Durbin is based. Amoxicillin will be manufactured by Medopharm private limited or other pharmaceutical companies recognised in United Kingdom where Durbin is based. Both azithromycin and amoxicillin are stable at room temperature. We will therefore ship and store in ambient conditions.

8.5.5 Preparation and labelling of study drugs

Study products will be stored at Malawi Liverpool Wellcome Trust Pharmacy. The pharmacy team will be responsible for packing and labelling.

8.5.6 Known drug reactions (adverse events)

Azithromycin and amoxicillin are already widely used in Malawi and are well tolerated. Rare side effects for azithromycin include nervousness, dermatologic reactions including Stevens–Johnson syndrome, anaphylaxis and prolonged QT interval. Rare side-effects for amoxicillin are mental state changes, light-headedness, photosensitivity and severe allergic reactions.

8.5.7 Concomitant medication and interaction with other therapies

We do not have any restrictions with respect to concomitant medications apart from those listed in the exclusion criteria. We expect some participants to be on HIV antiretroviral drugs and some may subsequently start tuberculosis therapy. Important interactions therefore would be those with HIV antiretroviral drugs and tuberculosis therapy. There is no moderate or major interaction between either azithromycin or amoxicillin with the classes of HIV antiretroviral drugs, tuberculosis therapy, and antimalarial drugs used in Malawi.

8.5.8 Trial restrictions

We do not require participants to have any dietary restrictions. We will also accept co-administration with contraception. Our trial interventions can safely be used in pregnancy, so we will include pregnant women should they be eligible.

8.5.9 Assessment of compliance

On Day-8, we will document self-reported compliance adherence of study products.

8.5.10 Withdraw of interventions

The investigator may also terminate a participant from study product if indicated by an adverse reaction. If a participant stops taking study product either voluntarily or by investigator decision, they will be encouraged to remain in follow up and their data will form part of intention to treat analyses.

8.6 Statistical approach

We will summarise the processes of recruitment including non-eligibility and reasons of exclusion in a CONSORT flow chart. We will describe the study participants by their baseline characteristics

Approved by
College of Medicine
13-Feb-2020
(COMREC)
Research and Ethics Committee

which we will report for each arm. We will perform analyses of all our outcomes based on an intention to treat analysis (using the arm patient was randomised to), adjusting for centre. We will make the following comparisons:

i) azithromycin or amoxicillin versus standard of care

ii) azithromycin versus standard of care

iii) amoxicillin versus standard of care

We will perform data cleaning and analysis using Stata release 15 (Stata Corp, College station, Texas, USA).

The following are descriptions of each outcome and corresponding statistical approach. The statistical approach will be expanded in a detailed statistical analysis plan, separate to the protocol, which will be finalised before unblinding the study data.

8.6.1 Primary outcome

The clinical trial has two separately powered, and distinctly assessed primary outcomes, one for diagnostic evaluation (Primary outcome 1: Day 8) and the other for clinical impact (Primary outcome 2: Day 29) of the intervention.

8.6.1.1 Primary outcome 1: Specificity of day 8 symptom change versus mycobacteriology

Investigational test The investigational test is change in symptoms at Day 8 categorised as: improved or not improved (no change plus worsened) in response to the following question: *on day 1, you reported that you were unwell; compared to that day, has your illness worsened, remained the same, or improved?*

To minimise ascertainment bias in ascertaining this endpoint, the evaluation of improvement of baseline symptoms will be captured using a self-interview platform: Audio Computer Assisted Self-Interview (ACASI). After orientation, the participant will be left alone in the room to interact with the computer. ACASI on Day-8 will precede all other interaction with research staff and clinical assessment/decision making. We will report ACASI interview outcome as:

- **ACASI-test-negative** if the participant reports improvement
- **ACASI-test-positive** if the participant reports no change or worsening.

Reference test

Mycobacteriology reference standard will be defined in participants with at least one specimen with a valid result on days 1 and 8 as:

- **Sputum-test -POSITIVE**: if at least one positive smear microscopy, Xpert/MTB/RIF, or MTB culture on sputum samples taken.

Approved by College of Medicine 13-Feb-2020 (COMREC) Research and Ethics Committee

- **Sputum-test-NEGATIVE**: none of the day 1 and day 8 sputum samples are positive on smear microscopy, GeneXpert MTB/RIF, or MTB culture.

To minimise bias, the mycobacteriology will be performed by a high-quality research laboratory in the University of Malawi College of Medicine by staff with no access to participant treatment allocation information or ACASI results.

The diagnostic assessment outcome

Proportion of participants correctly classified as PTB negative based on report of improvement of baseline symptoms on study Day-8 ACASI against a mycobacteriology reference standard (b+d in Figure 6). Using the investigational test and reference test described above, this can be rewritten as: proportion of sputum-test-negative participants who are ACASI-test-negative.

		**Reference Result:** *any positive smear microscopy, Xpert/MTB/RIF, or MTB Culture from sputum samples collected on Day 1 and Day 8 visit defines tuberculosis-test-positive*	
		Sputum-test-positive	Sputum-test-negative
**ACASI* Response on Day 8** *ACASI test is defined by response to the following question asked using ACASI on Day 8: on day 1, you reported that you were unwell; compared to that day, has your illness worsened, remained the same, or improved?*	ACASI-test-positive (worse or no change)	**a**	**b**
	ACASI-test negative (Improved)	**c**	**d**

Primary outcome: specificity, calculated by **d / (b+d)**

*Audio Computer Assisted Self-Interview (ACASI) in which the participant, after a how-to-use test session, responds to the prescribed question on a database-linked android tablet, without any human interaction, and in private.

Figure 6: Ascertainment of diagnostic value of trial-of-antibiotics

Estimation of measures of effect

We will use a generalised linear model (GLM) with identity link to estimate risks differences and the GLM with log link to estimate risk ratios for the three comparisons, adjusting for center. For each comparison, we will report 95% Confidence Intervals and Chi-square p-values. In pre-specified subgroup analysis, we will estimate the treatment effects stratifying by baseline HIV status. If the GLM model does not converge, we will use logistic regression to estimate the treatment effect using an odds ratio.

Approved by
College of Medicine
13-Feb-2020
(COMREC)
Research and Ethics Committee

Participants without valid sputum mycobacteriology result

Primary analyses will be limited to participants who have at least one valid sputum sample result from all samples collected on visits Day-1 and Day-8. However, in real-life, ~15% fail to produce sputum, we will as a secondary outcome, perform all the analyses described for primary outcome with these participants defined as mycobacteriology negative. Further sensitivity analyses with urine lipoarabamannan antigen (LAM) results will include them in mycobacteriology definition.

8.6.1.2 Primary outcome 2: Clinical benefit of trial-of-antibiotics

Outcome definition

Proportion of participants experiencing at least one of the following adverse outcomes: death, hospitalisation, and missed TB diagnosis. The definitions of the components of this composite clinical outcome are defined in the table below:

Outcome component	Definition
death	Proportion of deaths by Day 29
hospitalisation	Proportion hospitalised for any cause by Day 29
missed TB diagnosis	Day 29 proportion of participants meeting standard **mycobacteriological and radiological** TB definitions but incorrectly classified as TB negative and not yet on TB treatment by Day 29.

Estimation of measures of effect

We will use a generalised linear model (GLM) with identity link to estimate risks differences and the GLM with log link to estimate risk ratios for the three comparisons, adjusting for primary care center. For each comparison, we will report 95% Confidence Intervals and Chi-square p-values. If the outcome is rare or if GLM does not converge, we will use logistic regression to model odds and report odds ratios for the following comparisons and their associated report 95% CIs and p-values.

8.6.2 Secondary outcomes

Outcome definitions

1) Proportion of day 29 nasopharyngeal *Streptococcus pneumoniae* isolates resistant to any of the commonly used antimicrobials.

Approved by
College of Medicine
13-Feb-2020
(COMREC)
Research and Ethics Committee

We will define **AMR positive** as having nasopharyngeal isolates of Streptococcus pneumoniae that are resistant to any of the following commonly used antibiotics: ceftriaxone, amoxycillin, cefoxitin, azithromycin, and erythromycin as determined using disc diffusion technique; and **AMR negative** as either (1) not isolating any Streptococcus pneumoniae or (2) isolating any Streptococcus pneumoniae that is not resistant to any of the assessed antibiotics. For each arm, and at both baseline and day 29, we will report proportion of AMR positive participants. The study outcome will be the proportion of AMR positive participants at day 29.

2) Proportion of participants correctly classified as PTB negative based on report of improvement of baseline symptoms on study Day-8 (i.e. after a trial-of-antibiotics if in azithromycin or amoxicillin arms, or without antibiotics if in standard of care arm) against a mycobacteriology reference standard, among all randomised participants, with those who do not have a valid sputum test result classified as mycobacteriologically negative.

Estimation of measures of effect

Our secondary outcomes are anticipated to be rare, we will therefore use logistic regression to model odds and report odds ratios for the following comparisons and their associated report 95% CIs and p-values.

8.6.3 Exploratory outcome

Our exploratory analyses will be comparisons between the **azithromycin** and **amoxicillin** arms for all our primary and secondary outcomes.

8.6.4 Planned subgroup analyses

We will perform subgroup analysis for the primary outcome. The important subgroups based on rationale detailed under section 2.4.4, include HIV status, ART status, and PTB treatment. HIV and ART status will be as documented on Day-1 while PTB treatment will be either as:

- TB treatment commenced based on positive baseline (Day-1 and Day-8) mycobacteriology, *or*
- TB treatment commenced within 29 days of enrolment in patients with negative Day1 and Day-8 bacteriology.

The 29 days cut off for clinical decision to treat is to ensure that we only capture TB disease that was present at baseline. 29 days is a reasonable because: TB is a slowly progressing disease which if positive at Day-29, must have been incident on Day-1; and in routine care setting it can take over a month from presentation to diagnosis of TB.[8]

8.7 Sample size and power

8.7.1 Primary outcome1: specificity of day 8 symptom change versus mycobacteriology

Approved by
College of Medicine
13-Feb-2020
(COMREC)
Research and Ethics Committee

We assume that trial-of-antibiotics (in azithromycin arm or in amoxicillin arm) will correctly classify 60% of mycobacteriology negative participants.[14] We have determined that 400 mycobacteriologically negative (true negatives) participants per arm will provide 80% power to detect a 10% difference in proportion of participants correctly classified as negative by amoxicillin arm or by azithromycin arm (60%) versus standard of care arm (50%). See table 3. We assume that 80% of participants randomised will have negative mycobacteriology,[30] requiring 500 participants to yield the 400 per arm. Assuming that 15% will not be able to produce sputum, and that 5% will not return for Day-8 visit, the sample size is increased to 625 per arm or 1,875 for the whole study.

For a 2:1 comparison (combining the two antibiotic arms versus the standard of care arm), 305 sputum-test-negative participants per arm will be needed to achieve 80% discriminatory power to detect a 10% difference in specificity. Accounting for TB prevalence, ability to produce and submit sputum, and loss-to-follow up increases the sample size requirement to 472 per arm or 1,416 for the whole study.

Table 3: Power and sample size estimation for primary outcome

**True negatives** (mycobacteriology tests negative participants) **b+d**	[1]**p (negatives correctly classified) d/(b+d)**	[2]**effect size**	**power (X^2 difference between independent proportions)**
320	0.60	0.10	69%
**400**	**0.60**	**0.10**	**80%**
480	0.60	0.10	86%

[1] specificity with either azithromycin or amoxicillin trial-of-antibiotics arms

[2]risk difference (azithromycin arm- standard of care arm)

8.7.2 Primary outcome 2: Incidence of adverse clinical outcome at Day-29

We will use a pilot study to determine the standard of care risk of at least one of death, hospitalisation, and missed TB diagnosis. The pilot study is described in section 5.0.

For now, we will assume that there is a 10% risk of experiencing this composite adverse outcome in the standard of care arm, and that loss to follow up by Day-29 will be 10%. With the sample size of 625 participants per arm (based on the primary outcome 1 sample size calculation), and alpha of 0.05, we will be able to detect the difference between intervention and standard of care with 80% power, if the risk in intervention arm is 6% or lower (Table 4). This estimate is applicable to the 2:1 comparison of the study arms.

Table 4: Sample size estimation for clinical benefit outcome

**Participants per arm based on primary**	**625**

Approved by
College of Medicine
13-Feb-2020
(COMREC)
Research and Ethics Committee

10% loss to follow up by Day-29	562
Outcome risk in standard of care arm	0.10
Desired power	0.80
Alpha	0.05
**Required intervention arm risk**	**0.06**

8.7.3 Secondary outcome

1) **Incidence of resistant *S. pneumonia* on Day-29**

Study arms will be compared based proportion of participants with resistant *Streptococcus pneumoniae* on day 29. We assume 10% loss to follow up by Day-29, and the rate of *S. pneumonia* isolation from nasopharyngeal swabs in this population is expected to be ~45% at Day-29. The sample size based on the primary outcome (625 per arm) will provides ~253 *S. pneumonia* isolates/arm. In the standard of care arm, with 10% risk of resistant isolates, this translates into 25 cases. For the intention to treat population (the randomised 625 participants/arm) in the standard of care arm the 21 cases of resistant isolates translate into 4% (25/625) risk. To detect a twofold change in odds of day 29 AMR risk with at least 80% power, alpha of 0.05, and using Pearson's Chi-squared test, we will need at least 431 and 553 participants per arm for the 2:1 and pairwise comparisons respectively.

8.7.4 Exploratory outcomes

We anticipate that our sample size will be enough for hypothesis generation around our exploratory objectives but may not be enough to provide discriminatory power for comparison of outcomes between arms.

Approved by College of Medicine 13-Feb-2020 (COMREC) Research and Ethics Committee

9 Pilot study

This area of research has limited evidence to guide the precise determination of sample size and the practical aspects of the clinical trial making a pilot study an invaluable tool. We have identified the following as key knowledge gaps which require exploration using a pilot study:

1) Among the adult patients presenting to primary care centres with cough for at least 2 weeks what proportion gets antibiotics:
   - **a.** before clinic presentation?
   - **b.** on first clinic visit?
   - **c.** on follow up clinic visit after mycobacteriology results?
2) Following antibiotic treatment, how do patients report their clinical response? What are the best questions to ask patients post-antibiotic treatment to determine if they have improved or not? How best can we deliver these questions via Audio Computer Assisted Self-Interview (ACASI)? How well do these responses correlate with mycobacteriology and radiology?
3) What is the best timing for nasopharyngeal swabs for evaluating AMR in patients who receive a course of antibiotics during TB investigations?
4) In the standard of care setting, what proportion of adult patients presenting to primary care centres with cough for at least 2 weeks experience the following adverse outcomes (as defined under the clinical benefit composite endpoint)?
   - **a.** death
   - **b.** hospitalisation
   - **c.** missed TB diagnosis
   - **d.** HIV care loss to follow up
   - **e.** TB care loss to follow up

9.1 Specific objectives of the pilot study

1) To determine the proportion of adults with prolonged cough who
   - a. present to primary care having already had antibiotics for the index clinical complaints.
   - b. receive antibiotics before sputum mycobacteriology results at first presentation
   - c. receive antibiotics after negative mycobacteriology
2) To establish an objective way of documenting response to antibiotic treatment using Audio Computer Assisted Self Interview (ACASI). Assessing ACASI responses against clinical signs, outcomes of TB mycobacteriology and chest radiography.
3) To determine:

Approved by
College of Medicine
13-Feb-2020
(COMREC)
Research and Ethics Committee

a. the prevalence of *Streptococcus pneumonia*;
b. the prevalence of resistant *Streptococcus pneumonia* isolates;
c. the optimal specimen collection timing for evaluating impact of antibiotic use on prevalence of *Streptococcus pneumonia* isolates resistant to common antibiotics

4) To establish standard of care rates of the following adverse clinical outcomes:

a. death
b. hospitalisation
c. missed TB diagnosis
d. HIV care loss to follow up
e. TB care loss to follow up

9.2 Population for the pilot study

This exploratory study will include up to 400 adult (≥18 years old) patients presenting to primary care centres with cough for at least 14 days. We will exclude patients not meeting the eligibility criteria of the clinical trial.

9.3 Pilot study procedures

The pilot study procedures are outlined in the flow chart below. Following pilot study informed consent, we will use a baseline assessment questionnaire to collect clinical history, and antibiotic use for the index illness prior to the clinic visit. Throughout follow up, we will record all antibiotic use from any source. We will collect sputum samples for mycobacteriology from all participants on Day1 and Day 8.

We will establish HIV and TB diagnosis throughout the study, link participants to care services, and follow their adherence to follow up. For TB we will use a combination of Xpert, smear and culture on Day 1, 8 and whenever symptomatic suggestions of TB arise. We will also perform a chest x-ray on Day 8 and a follow up film on Day 29.

We will collect nasopharyngeal swab samples, for antimicrobial resistance assessment using *Streptococcus pneumonia* culture and sensitivity, on Day 1, Day 8, and Day 29. We will assess change in symptoms and well-being from Day 1 to Day 8, by using various combinations of questions and answers delivered via Audio Computer Assisted Self Interview (ACASI) on Day 8. We will ask participants which sets of questions they found easy to understand. We will also collect clinical information on all study visits including illness events, hospitalisations and vital status.

Approved by
College of Medicine
13-Feb-2020
(COMREC)
Research and Ethics Committee

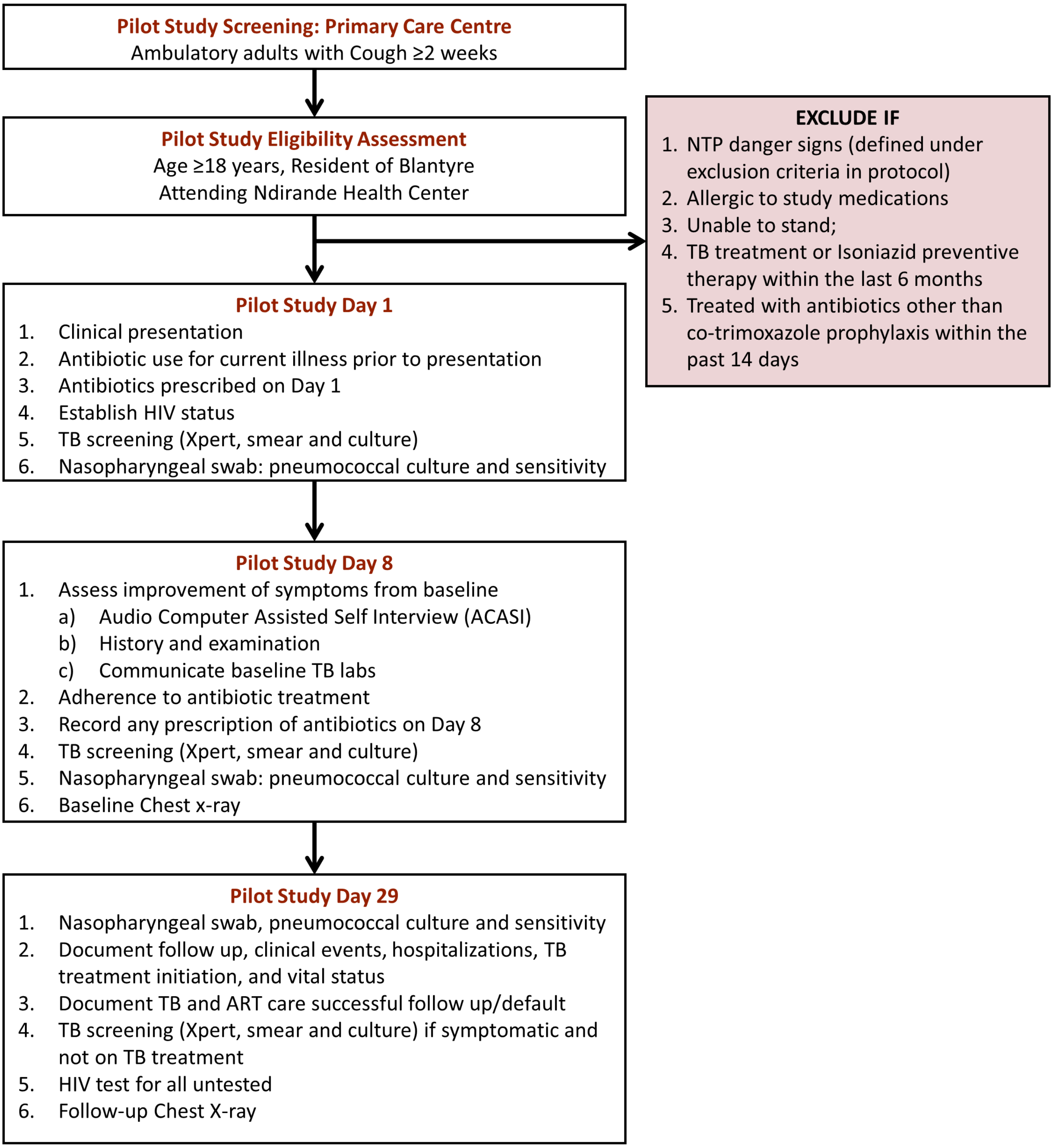

Pilot study flow diagram, summarizing the study procedures at each visit.

9.4 Data analysis

We will report the proportions of participants who used any antibiotics prior to primary care and during work-up for Tuberculosis. We will determine the best ACASI question and response combinations by participant reported ease of use, and by assessing correlation with clinical findings, mycobacteriology and radiological outcomes. The optimal time for assessing AMR will be determined by comparing incidence of resistant *Streptococcus pneumonia* isolates at days 8 and 29.

We will comparing participants exposed to antibiotics to those not exposed to antibiotics by estimating and reporting relative risk and 95% confidence intervals for:

Approved by College of Medicine
13-Feb-2020
(COMREC)
Research and Ethics Committee

1) Day 8 and Day 29 of resistant Streptococcus pneumonia
2) Composite adverse outcome of experiencing any of: death, hospitalisation, missed TB diagnosis, HIV care loss to follow up, and TB care loss to follow up

Approved by
College of Medicine
13-Feb-2020
(COMREC)
Research and Ethics Committee

10 Study procedures

10.1 Screening

At the designated primary health care centres, study staff will approach patients with symptoms of pulmonary tuberculosis (including cough of any duration, fever, weight loss, and night sweats) with information about the study. Those willing to be screened for eligibility will be assessed against the study inclusion and exclusion criteria.

10.2 Informed consent

We will seek written informed consent (Appendix 1) from all patients who meet eligibility criteria before any trial-specific procedures. Screening for tuberculosis symptoms will not be considered as part of the study procedures, as it is already a fundamental component of the routine clinical assessment and history taking. A member of the study team will hand an informed consent form to a potential participant in their preferred language (Chichewa or English) detailing background, procedures, risks, benefits and participant expectations should they choose to join the study. The consent form will also state that the participant is free to withdraw from the trial at any time for any reason without prejudice to future care, and no obligation to give reason for the withdrawal.

If they choose to join as a study participant, we will then request them to sign two copies of informed consent form. If a potential participant does not know how to read or write, we will perform the informed consent process in the presence of a witness. In such cases, if they agree to participate in the study, we will ask them to sign using a thumb-print in the presence of their witness and a study team member. We will keep one copy of the signed informed consent forms and hand the participant the other copy.

10.3 Baseline procedures

After consenting, we will on the same visit request participants to provide 2 on the spot sputum samples for smear microscopy, Xpert and culture collected at least one hour apart. Those unable to spontaneously produce sputum will be instructed in the physiotherapy manoeuvre of “huffing” (forced expiration technique) for inducing mucus clearance from the airways.

Patients still unable to provide at least one mucoid sputum sample of >1 ml will initially will be given a sputum container and asked to return it the next day. If they do not manage to produce sputum at home, their mycobacteriology results will be treated as missing. We expect ~15% of participants to fall in this category[30] and have accounted for them in the sample size estimation. For participants who produce less than the needed quantity of sputum, we will process them for the planned tests in the following priority order: 1) Xpert MTB/RIF, 2) MTB culture, 3) smear microscopy. The Xpert MTB/RIF is the single most important guide for immediate clinical diagnosis and the MTB culture is the single most accurate reference diagnostic.

We will also collect a urine sample which we will store for subsequent lipoarabamannan antigen detection (LAM); and a nasopharyngeal swab for pneumococcal culture and sensitivity testing to

Approved by
College of Medicine
13-Feb-2020
(COMREC)
Research and Ethics Committee

estimate prevalent antimicrobial resistance. We will also perform HIV tests according to the national algorithm, and if positive we will do HIV viral load. After completing all Day-1 visit procedures, we will link the newly tested positive participants to routine HIV care and will document when they start ART (Malawi National Program provides same-Day-ART initiation to all newly-diagnosed or untreated HIV-positive patients). After the sample collections, we will collect the following information:

- Demographic data, including precise geographic locator information using ePAL geolocation software (to aid follow-up). The locator information will also include phone numbers for the participant and for up to 3 family, or friends they nominate as alternative contacts.
- Clinical history including information on tuberculosis symptoms and health care seeking for HIV and tuberculosis care services including ART, cotrimoxazole, isoniazid preventive therapy, and past TB treatment.
- Vital signs including height and weight

After completing all these baseline procedures, we will randomise the participants to the three study arms.

10.4 Assignment of interventions

Step 1: An independent statistician based at LSHTM and without contact with participants or the study staff that see participants, will use the ralloc command in Stata (StataCorp LLC, College Station, Texas USA. Release 15.0) to prepare a random allocation sequence in advance of study recruitment efforts. Randomisation will be 1:1:1 to the three arms of the trial, block-randomised with variable block sizes, and stratified by primary care centre.

Step 2: Each treatment allocation will be printed alongside a randomisation number onto a pdf document.

Step 3: The statistician will email the pdf document to an independent designee within the University of Malawi who will print and place the randomisation assignments in envelopes labelled with randomisation numbers. The independent designee will hand the envelopes directly to the study pharmacist who will also receive a shipment of study medications. The pharmacist will store the envelopes in a secure location within the pharmacy.

Step 5: The pharmacist will pre-pack 625 each of protocol doses of azithromycin and amoxycillin without any reference to the allocation sequence. There is no need to refer to the allocation sequence for this step because the dosage for both treatments is the same and the total number of allocation for each treatment is known.

Step 6: At the beginning of each working week and upon request from study site staff, the study pharmacist will hand to site coordinators of each primary care center, a recruitment-rate-driven working stock of 1) the sequentially numbered sealed opaque envelopes containing randomization numbers and corresponding treatment allocations, and 2) study drugs.

Approved by
College of Medicine
13-Feb-2020
(COMREC)
Research and Ethics Committee

Step 7: Study staff from each site will conduct patient eligibility assessments. Patients meeting the quick criteria of age and cough for ≥14 days, will be assigned screening IDs before being taken through the full eligibility criteria and consenting process. Participants will be considered eligible and ready for randomisation after they meet all criteria and sign consent.

Step 8: Upon signing consent, the participant will be taken to the site-coordinators (nurse or clinical officer) who will assign them the next available study ID number and document it on their paper and electronic eligibility checklist and enrolment CRF. The study ID number will be the number on the treatment allocation envelope plus a site-specific code. They will then open the envelope, document the treatment assignment, to the participant's enrolment paper and electronic case report forms as well as on a study card that will be pasted in the participant personal health profile book.

Step 9: The coordinator will double-check to ensure that the enrolment number and the treatment assignment are recorded correctly. They will then record screening date, screening ID, randomisation date, study ID, and randomisation arm on an enrolment log. They will then administer the allocated treatment. Administering study medications will not be considered as prescribing considering that prescription to all eligible participants will have already been done by the study protocol.

Step 10: When the stock of either envelopes or study drugs runs out, the nurse-coordinators or designee will reorder a from the study pharmacist.

Additional details

All steps of receipt and utilization of the allocations and study drugs are elaborated in a detailed SOP. The SOP guides implementation of the above plan as far as possible and in line with site conditions.

The study drugs will be pre-packed blindly without any reference to treatment allocations ensuring that neither the pharmacist nor the nurse-coordinator know the treatment allocations until just before assigning to a participant.

10.5 Blinding

We will mask the treatments as far as possible. The study pharmacist will remain blinded as they will use the randomly- allocated label numbers to prepare and pack the correctly dosed study medications in opaque packaging. Study outcome assessment will occur without reference to study treatment allocation. All laboratory forms for mycobacteriology and nasopharyngeal pneumococcal work will have no reference to participant treatment allocation. On Day-8, assessment of improvement from baseline symptoms will utilize audio computer-assisted self-interview (ACASI) to minimise potential for social-mediated reporting and ascertainment biases (see Procedures Section of the protocol). All clinical endpoints assessment case report forms will bear no reference to treatment arm. However, we will keep participants, research coordinators, and routine care staff

Approved by
College of Medicine
13-Feb-2020
(COMREC)
Research and Ethics Committee

unmasked to ensure safety of the participants and allow appropriate patient management decision-making which may be related to the trial interventions.

10.6 Participant follow up

Following enrolment and completion of baseline procedures we will ask participants to return for follow up visits on days 8, and 29. They will be given 1 sputum collection bottle when leaving the clinic on Day-1 to bring with them sputum for mycobacteriology planned for Day-8 ("morning" specimen) followed by collection of one further "spot" sputum on Day-8, 2 sputum samples in total). We will also collect a second urine sample for storage for subsequent LAM antigen testing. Patients unable to produce at least one mucoid sputum sample of >1ml on Day-8 will be assumed for purposes of analysis to be mycobacterially negative for the Day-8 sputum samples. We are performing two sets of sputum examinations (Day-1 and Day-8) for each participant to strengthen the accuracy of the reference standard. Considering that TB progresses very slowly, making a diagnosis on Day-8 is not different from that made on Day-1.

We will advise participants that their sputum TB test results will start becoming available from 48 working hours after collection, but with the last test (MTB culture) taking up to 4 weeks. Patients will be advised that they will not be routinely contacted if positive TB test results become available before their Day-8 appointment (as is standard for outpatient management without danger signs in Malawi), and so will be advised to report promptly back to the clinic (with refund of transportation given) if they experience any clinical deterioration during Days 2 to 7. In the circumstances where TB treatment is commenced before completion of antibiotics prescribed for trial-of-antibiotics (amoxicillin and azithromycin), we will ask them to carry on with their allocated intervention together with the TB treatment.

10.6.1 Day-8 activities

On Day-8, the first activity before the participants undergo all other evaluations will be documentation of self-reported improvement of baseline (Day 1) TB symptoms using a pilot-validated set of questions and answer options delivered via Audio Computer Assisted Self Interview (ACASI). We will use ACASI with the goal of eliminating inter-observer variability and patient/interviewer reporting or ascertainment biases. After a "how to use" orientation and testing session, the participant will be left alone in the room to interact with the computer. A pre-recorded interviewer will ask the participant questions related to how their symptoms have changed on that day compared to how they were on Day-1 and will offer categorised voice-recorded responses with touch screen response buttons. The ACASI questionnaire will also include questions about adherence to study arm drugs and any other medical care (including traditional medicine) sought during the previous week.

Other activities for all participants on Day-8 include:

- collection of a second sputum sample for mycobacteriology tests.

Approved by
College of Medicine
13-Feb-2020
(COMREC)
Research and Ethics Committee

- providing participants with Day 1 smear and Xpert results linking those with positive tests, ongoing symptoms and other illnesses with routine care for appropriate management.
- clinical history detailing clinical events since enrolment.
- documentation of any medications including antimicrobials and traditional medicine outside the study
- providing a study Day-29 appointment card

For participants with negative Day-1 mycobacteriology results we will perform clinical evaluation after ACASI and will inform the patient that any positive Day-8 sputum mycobacteriology results will be reported actively (via telephone or house visit) as soon as quality-assured results become available (within 48 working hours for microscopy and Xpert). Patients who have not had complete resolution of symptoms will be referred with all available results to routine primary care management.

10.6.2 Day-29 activities

Day-29 will be the final study visit. We will on this visit, collect data on clinical impact of antibiotic treatment and risk of AMR. In line with the second primary endpoint (composite clinical impact), we will document:

1) vital status
2) hospitalisations
3) identify missed TB diagnosis by using repeat mycobacteriology and routine care radiology for the symptomatic
4) perform HIV tests for those with unknown status and eligible for routine HIV test

We will collect information on clinical events prior to and at the visit and communicate all available sputum culture results. After collecting the clinical information, we will collect nasopharyngeal swab sample for assessing antimicrobial resistance. To collect the sample, a trained study staff will swab the participants' nasopharynx and place the swab in a tube containing skim milk tryptone glucose glycerol (STGG).

10.7 Laboratory methods

10.7.1 Tuberculosis mycobacteriology

We will process mycobacteriology tests at the Malawi College of Medicine TB laboratory, a reference laboratory located in Blantyre. For sputum samples collected on Day-1, we will perform smear microscopy, Xpert MTB/RIF and MTB culture. For sputum samples collected on Day-8, we will perform smear microscopy and MTB culture. We will use Mycobacteria Growth Indicator Tube (MGIT) and Lowenstein-Jensen (LJ) culture methods for TB culture. Once isolated, we will perform speciation as *Mycobacteria tuberculosis* (MTB) or non-tuberculous mycobacteria (NTM) using MBP84 antigen testing, microscopic cording and, if necessary, morphology and growth

Approved by
College of Medicine
13-Feb-2020
(COMREC)
Research and Ethics Committee

characteristics at different temperatures and on solid (LJ) media containing p-nitrobenzoic acid (PNB).

10.7.2 Urine antigen testing for lipoarabamannan and other MTB antigens

Urine will be collected and stored as two 1 ml aliquots at -20°C from each participant on both Day-1 and Day-8 for subsequent mycobacterial antigen testing. No appropriate product is available for immediate use, but we anticipate that a commercial product with sufficiently high analytic accuracy for use in ambulant outpatients (sensitivity and specificity) may become available during the course of, or soon after, the study. If ongoing evaluations of the FIND-sponsored FujiFilm product[44] meet or exceed pre-specified requirements for clinical utility in the outpatient context, then point-of-care LAM testing at Day-1 and Day-8 will be added to the mycobacteriological definition of TB and patient management as soon as kits have been obtained and evaluated in Malawi.

10.7.3 Antimicrobial resistance testing

We will store swabs in STGG at minus 80°C. At a later stage we will thaw them in batches, and plate them onto selective media and culture colonies consistent with *S. pneumoniae*. We will determine Minimal inhibitory concentrations (MICs) using E-test strips (azithromycin and amoxicillin), and Kirby Bauer Disc diffusion testing (azithromycin, rifampicin, tetracycline, ceftriaxone, chloramphenicol, co-trimoxazole, erythromycin and penicillin) and define resistance by EUCAST breakpoints.

We will store isolates and remaining STGG at minus 80°C to allow genotypic characterization, isolation and susceptibility testing of other key respiratory pathogens, FTD 33 respiratory pathogen diagnostic panel, metagenomics analysis, and microarrays to detect multiple carriage and macrolide resistance genes in a broader range of pathogens at a later stage.

10.8 Loss to follow-up

To minimise loss to follow up, we will at enrolment record geolocation information of participants' place of residence using ePAL android app, a high-resolution mapping system validated in Blantyre. We will also record up to 3 contact phone numbers of the participant and their nominated friends and relatives. Should a participant miss a study visit, we will contact them by phone or by visiting them at home to encourage them to attend the study visit before expiry of prescribed visit window.

We anticipate a loss to follow-up of 5% by Day-8, and 10% by Day-29. We have accounted for these assumptions in the sample size calculation. We will not replace participants who discontinue study participation or study treatment regardless of reason for withdrawal or discontinuation or the time either of these occurs.

10.9 Trial closure

We will consider the trial closed after completing follow up of the last enrolled participant, and upon recording all mycobacteriology laboratory reports. Antimicrobial resistance lab work will continue beyond trial closure. The trial may be terminated early by the trial steering committee upon

Approved by
College of Medicine
13-Feb-2020
(COMREC)
Research and Ethics Committee

recommendation of the DSMB. The halting rule for a trial arm is an unacceptable high level of deaths assessed using an alpha determined at the first DSMB meeting.

10.10 Summary schedule for study procedures

In Table 5 below, we have summarised all key study procedures over the study period.

Approved by
College of Medicine
13-Feb-2020
(COMREC)
Research and Ethics Committee

Table 5: key study procedures over the study period

	STUDY PERIOD			
	Enrolment	Follow up		
TIMEPOINT**	Day-1	Day-8		Day-29
**ENROLMENT:**				
Eligibility screen	X			
Informed consent	x			
Allocation	x			
**INTERVENTIONS:**				
Azithromycin	x			
Amoxicillin	x			
Standard of care	x			
**ASSESSMENTS:**				
Demographics	x			
History of antibiotic use	x	x		x
**History & examination*	x	x		
***Sputum collection*	x	x		
Urine for TB LAM test	x	x		
Nasopharyngeal swab	x			x
HIV test and CD4 count	x			x
Linking to routine care	x	x		x
[1]*ACASI*		x		
****Clinical events*		x		x
Update contact & address		x		x

*For symptomatic participants, Day-8 sputum mycobacteriology should be fast-tracked to inform care before they leave the clinic.

**Give sputum bottles at end of Day-1 visit for submission on Day-8. Also collect sputum and perform mycobacteriology at any time of the study when clinically indicated

***Illnesses, clinic visits, radiological outcomes, new HIV diagnosis, new TB diagnosis, death, hospitalisation, missed TB diagnosis, HIV care loss to follow up, and TB care loss to follow up
[1]Audio Computer Assisted Self-Interview for documenting change of symptoms on Day- 8 versus Day-1

Approved by
College of Medicine
13-Feb-2020
(COMREC)
Research and Ethics Committee

11 Safety reporting

11.1 Definitions

Term	Definition
Adverse Event (AE)	Any untoward medical occurrence in a participant to whom a medicinal product has been administered, including occurrences which are not necessarily caused by or related to that product.<br><br>An AE can therefore be any unfavourable and unintended sign (including an abnormal laboratory finding), symptom, or disease temporally associated with the use of an investigational medicinal product (IMP), whether or not considered related to the IMP.
Adverse Reaction (AR)	Any untoward and unintended response in a participant to an investigational medicinal product which is related to any dose administered to that participant.<br><br>The phrase "response to an investigational medicinal product" means that a causal relationship between a trial medication and an AE is at least a reasonable possibility, i.e. the relationship cannot be ruled out.<br><br>All cases judged by either the reporting medically qualified professional or the Sponsor as having a reasonable suspected causal relationship to the trial medication qualify as adverse reactions.
Serious Adverse Event (SAE)	A serious adverse event is any untoward medical occurrence that:<br>• Results in death<br>• Is life-threatening<br>• Requires inpatient hospitalisation or prolongation of existing hospitalisation<br>• Results in persistent or significant disability/incapacity<br>• Consists of a congenital anomaly or birth defect<br><br>Other 'important medical events' may also be considered serious if they jeopardise the participant or require an intervention to prevent one of the above consequences.
Serious Adverse Reaction (SAR)	An adverse event that is both serious and, in the opinion of the reporting investigator, believed with reasonable probability to be due to one of the trial treatments, based on the information provided.
Suspected Unexpected Serious Adverse Reaction (SUSAR)	A serious adverse reaction, the nature and severity of which is not consistent with the information about the medicinal product in question set out:<br><br>• In the case of a product with a marketing authorisation, in the summary of product characteristics (SmPC) for that product.

Approved by
College of Medicine
13-Feb-2020
(COMREC)
Research and Ethics Committee

11.2 DMID grading for AEs

We will adopt the events grading criteria prepared by the Division of Microbiology and Infectious Diseases (DMID) of the USA National Institutes of Health as shown in the table below.

1 MILD	2 MODERATE	3 SEVERE	4 LIFE-THREATENING
Transient or mild discomfort (< 48 hours); no medical intervention required	Mild to moderate limitation in activity - some assistance may be needed; no or minimal medical intervention required	Marked limitation in activity, some assistance usually required; medical intervention required, hospitalizations possible	Extreme limitation in activity, significant assistance required; significant medical intervention required, hospitalization probable

11.3 Grading for expected events

The following table provides guidance for grading known important or frequent side effects of azithromycin (based on the AE grading criteria provided in the BREATHE Trial Protocol, also investigating azithromycin) and amoxicillin graded on the DMID scale. All events not mentioned here or in Appendix 2, will be graded using the DMID grading for AEs table presented above.

	1 MILD	2 MODERATE	3 SEVERE	4 LIFE-THREATENING
**Side-effects**	**1**	**2**	**3**	**4**
**Acute Allergic Reaction**	Localized urticaria (wheals) with no medical intervention indicated	Localized urticaria with intervention indicated OR Mild angioedema with no intervention indicated	Generalized urticaria OR Angioedema with intervention indicated OR Symptoms of mild bronchospasm	Acute anaphylaxis OR Life-threatening bronchospasm OR Laryngeal oedema
**Rash**<br>***Specify type, if applicable***	Localized rash	Diffuse rash OR Target lesions	Diffuse rash AND Vesicles or limited number of bullae or superficial ulcerations of mucous membrane limited to one site	Extensive or generalized bullous lesions OR Ulceration of mucous membrane involving two or more distinct mucosal sites OR Stevens-Johnson syndrome OR Toxic epidermal necrolysis
**Mental state changes**	mild anxiety or depression	moderate anxiety or depression; therapy required; change in normal routine	severe mood changes requiring therapy; or suicidal ideation; or aggressive ideation	acute psychosis requiring hospitalization; or suicidal gesture/attempt or hallucinations
**Photosensitivity**	Painless erythema covering <10%	Tender Erythema covering 10 -	Erythema covering >30% body surface area and	Life-threatening consequences; urgent intervention indicated

Approved by College of Medicine
13-Feb-2020
(COMREC)
Research and Ethics Committee

	1 MILD	2 MODERATE	3 SEVERE	4 LIFE-THREATENING
	body surface area	30% body surface area	erythema with blistering, requiring intervention	
**Arrhythmia (by ECG or physical examination)** ***Specify type, if applicable***	No symptoms AND No intervention indicated	No symptoms AND Non-urgent intervention indicated	Non-life-threatening symptoms AND Non-urgent intervention indicated	Life-threatening arrhythmia OR Urgent intervention indicated
**Prolonged QTc Interval**	0.45 to 0.47 seconds	> 0.47 to 0.50 seconds	> 0.50 seconds OR ≥ 0.06 seconds above baseline	Life-threatening consequences (e.g., Torsade de pointes, other associated serious ventricular dysrhythmia)
**Diarrhea ≥ *1 year of age***	Transient or intermittent episodes of unformed stools OR Increase of ≤ 3 stools over baseline per 24-hour period	Persistent episodes of unformed to watery stools OR Increase of 4 to 6 stools over baseline per 24-hour period	Increase of ≥ 7 stools per 24-hour period OR IV fluid replacement indicated	Life-threatening consequences (e.g., hypotensive shock)
**Tinnitus**	Symptoms causing no or minimal interference with usual social & functional activities with intervention not indicated	Symptoms causing greater than minimal interference with usual social & functional activities with intervention indicated	Symptoms causing inability to perform usual social & functional activities	NA
**Nausea**	Transient (< 24 hours) or intermittent AND No or minimal interference with oral intake	Persistent nausea resulting in decreased oral intake for 24 to 48 hours	Persistent nausea resulting in minimal oral intake for > 48 hours OR Rehydration indicated (e.g., IV fluids)	Life-threatening consequences (e.g., hypotensive shock)
**Vomiting**	Transient or intermittent AND No or minimal interference with oral intake	Frequent episodes with no or mild dehydration	Persistent vomiting resulting in orthostatic hypotension OR Aggressive rehydration	Life-threatening consequences (e.g., hypotensive shock)

Approved by College of Medicine
13-Feb-2020
(COMREC) Research and Ethics Committee

	1 MILD	2 MODERATE	3 SEVERE	4 LIFE-THREATENING
			indicated (e.g., IV fluids)	
**Laboratory**	**1**	**2**	**3**	**4**
**ALT or SGPT, High**<br>***Report only one***	1.25 to < 2.5 x ULN	2.5 to < 5.0 x ULN	5.0 to < 10.0 x ULN	≥ 10.0 x ULN
**Creatinine Clearance or eGFR, Low**<br>***Report only one***	NA	< 90 to 60 ml/min or ml/min/1.73 m2 OR 10 to < 30% decrease from baseline	< 60 to 30 ml/min or ml/min/1.73 m2 OR ≥ 30 to < 50% decrease from baseline	< 30 ml/min or ml/min/1.73 m2 OR ≥ 50% decrease from baseline or dialysis needed

11.4 Causality

When reporting on serious adverse events, the trial investigator will state whether they believe that the event is causally associated with any of the trial treatments and the strength of the causal relationship. They will also state whether the adverse event was expected and what if any action was taken.

Relationship	Description
Unrelated	There is no evidence of any causal relationship
Unlikely	There is little evidence to suggest there is a causal relationship (e.g. the event did not occur within a reasonable time after administration of the trial medication). There is another reasonable explanation for the event (e.g. the participant's clinical condition, other concomitant treatment).
Possible	There is some evidence to suggest a causal relationship (e.g. because the event occurs within a reasonable time after administration of the trial medication). However, the influence of other factors may have contributed to the event (e.g. the participant's clinical condition, other concomitant treatments).
Probable	There is evidence to suggest a causal relationship and the influence of other factors is unlikely.
Definitely	There is clear evidence to suggest a causal relationship and other possible contributing factors can be ruled out.
Not assessable	There is insufficient or incomplete evidence to make a clinical judgement of the causal relationship.

11.5 Reporting Procedures

11.5.1 Non-serious Adverse Events (AEs)

Approved by
College of Medicine
13-Feb-2020
(COMREC)
Research and Ethics Committee

Adverse events will be ascertained from patient follow-up visits or reports from relatives or guardian if patient cannot be contacted for follow-up. Study clinicians will be responsible for recording of details of the event including a description of the event, date of onset, severity, assessment of relatedness to trial interventions. Adverse events will be recorded in case report forms and uploaded into the study database.

11.5.2 Serious Adverse Events (SAEs*)*

All serious adverse events (SAEs) will be recorded on the relevant study CRFs and reported immediately to the Principal Investigator who will ensure that they are compiled in aggregate form and reported to COMREC and the DSMB once every 6 months. The DSMB will review SAE reports at their 6 monthly meetings and issue recommendations which will be shared with ethics committees. Events relating to a pre-existing condition or any planned hospitalisations for elective treatment of a pre-existing condition will not be reported as SAEs.

Approved by
College of Medicine
13-Feb-2020
(COMREC)
Research and Ethics Committee

12 Economic evaluation

12.1 Objective

The objective of the economic evaluation is to undertake a cost-utility analysis to estimate the incremental cost-effectiveness of trial-of-antibiotics using azithromycin and trial-of-antibiotics using amoxicillin in comparison to standard of care, and to each other. We will systematically compare costs and consequences associated with the interventions.

12.2 Outcomes

We will perform a within trial comparison of the three treatment arms to estimate the incremental cost per quality-adjusted life year (QALY) gained for the azithromycin or amoxicillin arm in comparison to standard of care. Costs will be estimated from the Malawian Ministry of Health perspective. Health outcomes will be quantified in QALYs, estimated from participants' responses to the Chichewa version of the EQ-5D-3L, a Health quality of life (HRQoL) measure.[45,46] We will adopt a time horizon matching the length of participant follow-up to achieve the within trial evaluation.

12.3 Data collection

The health economic data collection will be undertaken alongside planned clinical data collections. We will administer the Chichewa version of the EQ-5D-3L to all trial participants at baseline (Day1), Day 8 and Day 29. The Chichewa EQ-5D-3L was prepared in accordance with international and EuroQoL guidelines. The EQ-5D uses a descriptive system and a visual analogue scale (VAS). HRQoL on the day of response is defined using the descriptive system in terms of the following dimensions: 1) mobility, 2)self care, 3)usual activities, 4)pain/discomfort, and 5) anxiety or depression. The responses are then split into the following ordinal levels: 1) no problems; 2) some or moderate problems; and 3) severe or extreme problems.

The EQ-5D has 243 health states to which each response is allocated and converted to an EQ-5D utility score using a tariff. Tariff sets are derived from national surveys and currently no Malawian EQ-5D tariff exists. Zimbabwe, a setting similar to Malawi, has EQ-5D tariff set. In this study, we will use the Zimbabwean set to derive EQ-5D utility scores[47] an acceptable practice considering the similarities in how the two populations value health.[48] The EQ-5D utility scores in the Zimbabwean tariff, range from 1.0 (which means no problems in the five dimensions) to -0·29 (defined as severe problems in all five dimension).

We will capture all healthcare resources used by trial participants from recruitment into the trial till Day 29. This will be undertaken on Day 1, Day 8 and Day 29. Healthcare resources will be translated into direct medical costs using previously estimated costs[46,49,50] and the wider literature. Drug prices will be based on International market prices.[51] The health resource use questionnaire will at a minimum capture:

- Outpatient clinic visits

Approved by
College of Medicine
13-Feb-2020
(COMREC)
Research and Ethics Committee

- Days of inpatient hospital care
- Medications
- Investigations and procedures

12.4 Data analysis

Our primary analysis will focus on direct intervention and the broader healthcare costs. We will define direct intervention costs as the costs associated with the application of the interventions. We will plot health state values measured by the EQ-5D-3L against time assuming that the health states reported at each time point are linearly connected. We will estimate QALYs associated with participant health profile by area under the plotted curve as calculated using the trapezium rule.

We will use a range of analytical methods depending on whether baseline covariates (EQ-5D utility values) are balanced between the trial arms or not. If they are balanced, we can obtain unbiased cost-effectiveness estimates by using non-parametric bootstrap approaches; if imbalance exists regression methods will be the approach of choice.

We will explore a range of estimators and undertake model diagnostics to determine the optimal model because the distributions of costs and QALYs are commonly skewed, often bimodal, or truncated. We will estimate mean costs and outcomes for each intervention together with respective mean incremental cost-effectiveness ratio. We will for each estimate report respective measures of uncertainty (standard errors and confidence intervals). We will also estimate the net monetary benefits (NMBs) for a range of different willingness to pay (WTP) thresholds. To identify the optimal intervention at different WTP thresholds, we will construct cost-effectiveness acceptability curves (CEACs) based on the NMB framework.

12.5 Missing data

For each participant we will collect complete data as far as possible but in cases of missing values, a common occurrence in trials, we will perform additional analyses to explore the impact of and account for the missingness.

Approved by
College of Medicine
13-Feb-2020
(COMREC)
Research and Ethics Committee

13 Data management

13.1 Source Data

We will consider a document as source if it is where data were first recorded, and from which we obtained participants' case report forms (CRF) data. These will include hospital records, health center records, participant health passport, laboratory and pharmacy records, diaries, radiographs, and correspondence. We will consider CRF entries as source data if the CRF is the site of the original recording.

We will on all study-specific documents, other than study ID code list, the signed consent forms household locator form and, refer to the participant by their trial participant identification number, not by name. We will keep study ID code list, consent and locator forms separate from the rest of the participant file to avoid linkage between participant name and the study ID.

13.2 Data collection methods

We will collect data using standardised, pre-tested CRFs in two forms:

- programmed into android tablets using Open Data Kit (ODK) platform (opendatakit.org) with paper back-ups.
- optical mark recognition readable forms which will be read and extracted using TELEFORM system (Cardiff Software, Inc., Vista, CA), an optical-character-recognition software.

13.3 Data management

Any participants' identifiable data collected by the Study Coordination Centre will be stored securely and their confidentiality protected in accordance with the Data Protection Act 1998.

To ensure data security and maintenance of participant confidentiality, we will take several strict measures. All the study data collection tablets and computers will be encrypted, password protected and stored in a fireproof lockable cabinet inside a locked room. The principal investigator, study coordinator and data manager will be responsible for the maintenance of the tablets as well as all other computers, and their security from viruses and theft. All users will check in with the study coordinator and sign for data entry tablets every time they are taken out to for data entry and upon return. Whenever not in use, the devices will be kept in their locked cabinet.

We will keep all paper records in a locked space only be accessible to the principal investigator, co-investigators and delegated study staff. Study databases will be encrypted, password protected and will be stored on dedicated servers within the University of Malawi College of Medicine. We will keep all electronic and paper records securely for up to 10 years after the end of the trial in accordance with LSHTM Records Retention & Disposal Schedule guidelines.

Approved by
College of Medicine
13-Feb-2020
(COMREC)
Research and Ethics Committee

13.4 Quality control and quality assurance

We will apply quality control at each stage of data handling in accordance with GCP requirements to ensure that all data are reliable and have been processed correctly. We will manual review all paper CRFs for completeness, accuracy and legibility before scanning. Our ODK data entry system will include automatic pre-programmed real-time data validation. The TELEFORM system will also have pre-programmed automatic data validation capabilities. We will perform data quality assurance (QA) on a random 10% of all participant files. The QA process will involve examining database entries and for paper source documents, verification of database entries and source.

13.5 Access to data

We will upon request, provide direct access to authorised representatives from the Sponsor, host institution and the regulatory authorities to allow smooth running of trial-related monitoring, audits and inspections.

Approved by
College of Medicine
13-Feb-2020
(COMREC)
Research and Ethics Committee

14 Data monitoring and quality assurance

14.1 Data monitoring

Site monitoring for safety will be conducted to ensure human subject protection. The study will be monitored just before commencing enrolment, then once every 6 months by a monitoring team from the University of Malawi College of Medicine. The objective will be to ensure that study procedures, study products administration, and data collection processes are of high quality and meet ethical and regulatory guidelines. The regular monitoring will focus on the following areas: 1) protocol adherence, 2) informed consent documentation, 3) trial endpoints, 4) treatment discontinuation, 5) regulatory documents, 6) compare source documents and case report forms for accuracy, and 7) documentation practices in general.

14.2 Audits and Inspections

The study will be subject audit by the London School of Hygiene & Tropical Medicine under their remit as sponsor, the Study Coordination Centre and other regulatory bodies to ensure adherence to GCP.

14.3 Data Safety and Monitoring Board (DSMB)

We will set up a DSMB before commencing trial activities. The DSMB will provide independent review of the study conduct, progress and findings. It will comprise 3 members including a chairperson who will be responsible for collating and communicating the views of the DSMB. The DSMB will consist of an independent statistician and two clinicians, at least one of them a physician, with research experience and expertise in the management of tuberculosis and HIV in Africa. The proposed data safety monitoring plan will be discussed in a teleconference including the DSMB members and the key investigators prior to the study starting.

The proposed meeting schedule is 6 monthly. Two weeks before a 6 monthly DSMB meeting, the study team will prepare a report covering study progress, study approvals, any obstacles, and recruitment statistics, adverse events, withdrawals and trial outcome measures. The DSMB will, through its chairperson, provide written feedback to the principal investigator who will be responsible for passing it on to ethics committees.

14.4 Trial Management Group (TMG)

A Trial Management Group (TMG) will be appointed and will be responsible for overseeing the progress of the trial. The day-to-Day-management of the trial will be co-ordinated through the University of Malawi College of Medicine.

Approved by
College of Medicine
13-Feb-2020
(COMREC)
Research and Ethics Committee

15 Ethics and dissemination

We will ensure that this trial is conducted in accordance with the principles of the Declaration of Helsinki and in full conformity with relevant regulations and with the ICH Guidelines for Good Clinical Practice E6 (R2) of November 2016.

15.1 Risk assessment

This is a low risk study as it is using already licensed antibiotics with good safety profile in a population defined by national clinical guidelines as clinically stable and not requiring other intervention but TB investigations. Our work complements standard of care by bringing in detailed TB diagnostics. In our study, the standard of care equivalent of the antibiotics we will prescribed on Day-1 to those randomised to either azithromycin or amoxicillin arms, are in standard of care prescribed on Day-8 only to mycobacteriology negative symptomatic patients (similar to the no antibiotic or standard of care arm of our trial). So, participants randomised to no antibiotic at Day-1 will not be receiving inadequate care but the recommended standard management of withholding antibiotics until after the TB results are available (Figure 1). To maintain participant safety and continuity of their care while on study interventions, we will not blind routine care clinical team and they will be free to manage the participants on their clinical judgement and national guidelines.

15.2 Research ethics approval

We will seek ethical approval for the trial protocol, informed consent forms, participant information sheet, any advertising material, and amendments to any of these documents, from the University of Malawi College of Medicine Research and Ethics Committee (COMREC), the LSHTM Research Ethics Committee, and Regional Committee for Health and Research Ethics, NTNU-Midt, Norway (on behalf of the funder). We will seek regulatory approval from the Malawi Pharmacy, Medicines, and Poisons Board (PMPB). Every year when the trial is active, we will seek continuous ethical review and approval before expiry of previous year’s approval. In the event of an amendment, the changes will only be implemented upon ethical and regulatory approval.

15.3 Indemnity

London School of Hygiene & Tropical Medicine holds Public Liability ("negligent harm") and Clinical Trial ("non-negligent harm") insurance policies which apply to this trial.

15.4 Sponsor

London School of Hygiene & Tropical Medicine will act as the main sponsor for this study. Delegated responsibilities will be assigned locally.

15.5 Declaration of interests

The study team declares that they have no conflict of interest in conducting this clinical trial.

Approved by
College of Medicine
13-Feb-2020
(COMREC)
Research and Ethics Committee

15.6 Cost of participation, ancillary and post-trial care

During the study, participant will benefit from frequent interaction with clinical study staff and associated optimised management of illnesses. There are minimal risks including discomfort associated with collection of nasopharygeal samples, and side-effects of study interventions. We will reimburse participant transport for attending study visits.

15.7 Dissemination policy

This work will form part of a PhD thesis for Titus Divala, which he will submit to the London School of Hygiene & Tropical Medicine (LSHTM). This work will form part of a PhD thesis for Titus Divala, which he will submit to the London School of Hygiene & Tropical Medicine (LSHTM). We will share the results of this work with COMREC, LSHTM REC and Regional Committee for Health and Research Ethics at NTNU, Norway. The Malawi National TB Control Program are already aware of the study through our long standing collaborations. Apart from NTP, we will share our results with Blantyre District Health Office, the wider Ministry of Health, and the University of Malawi College of Medicine via the annual research dissemination conference. We will also prepare manuscripts for peer reviewed publications.

All publications and presentations relating to the study will be authorised by the Trial Management Group. The first publication of the trial results will be in the name of the Trial Management Group, if this does not conflict with the journal's policy. If there are named authors, these will include at least the trial's Chief Investigator, Statistician and Trial Coordinator.

Members of the TMG and the DSMB will be listed and contributors will be cited by name if published in a journal where this does not conflict with the journal's policy

Approved by
College of Medicine
13-Feb-2020
(COMREC)
Research and Ethics Committee

16 Study requirements, budget and justification

		CURRENCY	
**Study staff**	**Salary/month**	**GBP**	**MWK**
Clinical officers: 1 for 15 months	484	7,260	7,623,000
Nurses: 3 for 15 months	484	21,780	22,869,000
Clinic assistants: 4 for 12 months	300	18,000	18,900,000
Laboratory Technicians: 1 for 12 months	482	7,230	7,591,500
Data Clerks: 1 for 12 months	482	7,230	7,591,500
**Subtotal study staff**		**61,500**	**64,575,000**
**Materials and consumables**			
Trial participant costs: Stationary, printing, photocopying		1,000	1,050,000
Internet costs		1,500	1,575,000
Telephone airtime for for RCT staff/participant follow-up		1,500	1,575,000
International shipping (30% of UK consumable costs)		625	656,250
Transportation of field worker, airport transfers etc		3,000	3,150,000
Participant transport reimbursement		11,250	11,812,500
Antimicrobial resistance testing at MLW (paid to LSTM via LSHTM)		0	0
TB Xpert/MTB/RIF (Paid through LSHTM)		0	0
TB smr+culture @ £9.00 (subsidised by PhD supervisor)		6,300	6,615,000
Chest X-ray @ £5.00 (All participants Day 8, 15% on D56)		8,625	9,056,250
**Subtotal of materials and consumables**		**33,800**	**35,490,000**
**Misclaneous costs**			
MLW pharmacy fees (PhD student discount)		3,629	3,810,823
Data Safety & Monitoring Board & TSC		600	630,000
Medical indemnity @30/staff		420	441,000
Health and Safety including PEP		1,000	1,050,000
Translation costs		300	315,000
**Subtotal of Other**		**5,949**	**6,246,823**
**Other direct costs in COM**			
Infrastructure at CoM (3 lockable cabinets)		750	787,500
COMREC submission fees & amendments		300	315,000
Recruitment of clinical and non-clinical support staff		500	525,000
Protocol Training (for study staff)		1,000	1,050,000
GCP Training Facility		1,000	1,050,000
Clinical Trial Monitoring by COM RSC		1,500	1,575,000
**Subtotal of COM Costs**		**5,050**	**5,302,500**
**Total before overheads**		**106,299**	**111,614,323**
**Indirect costs**			
**COM 10% Overheads**		**10,630**	**11,161,432**
Malawi Pharmacy, Medicines and Poisons Board Trial Registration		5,532	5,808,871
Fronting Insurance (required for Malawi Trial Participants)		1,073	1,126,210
**Subtotal indirect costs**		**17,235**	**18096512.9**
**Grand total**		**123,534**	**129,710,835**

16.1 Budget justification

16.1.1 Study staff

Approved by
College of Medicine
13-Feb-2020
(COMREC)
Research and Ethics Committee

The study will require the services of 2 clinical officers, 2 research nurses, 2 clinic assistants, 1 laboratory technician, and 1 data clerk. The chief investigator a PhD student, will not draw a salary from the study budget as he already has a stipend. None of the co-investigators will draw a salary from the study as they are already paid by their respective institutions.

16.1.2 Materials and consumables

We will need to cover costs for stationary, printing and photocopying of research tools for the project. These include paper-based questionnaires, information sheets and consent forms. We will need funding to cover communication needs (telephone and internet) throughout the study. This will allow maintainance of supervision of the study team, and facilitate participant follow up. We will need to cover local ground transportation for study staff and participant tracing. We will reimburse participants transport costs during the study period.

We will perform various laboratory tests. All TB tests will be done at the COM-MLW TB reference lab in Microbiology building. The costs of all TB tests have been subsidised by PhD supervisor. One of the TB tests, Xpert/MTB/RIF, will be paid from the LSHTM component of the funding. For antimicrobial resistance analysis, we will collect 1578 nasopharyngeal swabs on day 1 and day 29 and perform pneumococcal culture and drug susceptibility testing in MLW laboratory. Payment for this will be paid to Liverpool School of Tropical Medicine from LSHTM.

16.1.3 Miscellaneous costs

We will pay for use of the MLW pharmacy. We will cover costs for coordination of Data Safety & Monitoring Board and Trial steering committee meetings. We will conduct protocol, GCP and human subjects training for study staff before commencement of the study. We will have funds for Health and Safety (including PEP) related costs. We will utilise clinical trial monitoring from COM-RSC. We will need some COM infrastructure support.

Approved by
College of Medicine
13-Feb-2020
(COMREC)
Research and Ethics Committee

17 References

1. Holmes AH, Moore LS, Sundsfjord A, Steinbakk M, Regmi S, Karkey A, Guerin PJ, Piddock LJ. Understanding the mechanisms and drivers of antimicrobial resistance. *The Lancet* 2016; **387**(10014): 176-87.
2. World Health Organization. The evolving threat of antimicrobial resistance: options for action: Geneva: World Health Organization; 2012.
3. World Health Organization. Antimicrobial resistance: global report on surveillance: World Health Organization; 2014.
4. Laxminarayan R, Matsoso P, Pant S, Brower C, Røttingen J-A, Klugman K, Davies S. Access to effective antimicrobials: a worldwide challenge. *The Lancet* 2016; **387**(10014): 168-75.
5. Raviglione M, Sulis G. Tuberculosis 2015: burden, challenges and strategy for control and elimination. *Infectious disease reports* 2016; **8**(2): 6570.
6. World Health Organization. Global tuberculosis report 2016. 2016.
7. Nliwasa M, MacPherson P, Mukaka M, Mdolo A, Mwapasa M, Kaswaswa K, Msefula C, Chipungu G, Mwandumba H, Corbett E. High mortality and prevalence of HIV and tuberculosis in adults with chronic cough in Malawi: a cohort study. *The international journal of tuberculosis and lung disease* 2016; **20**(2): 202-10.
8. Getahun H, Harrington M, O'Brien R, Nunn P. Diagnosis of smear-negative pulmonary tuberculosis in people with HIV infection or AIDS in resource-constrained settings: informing urgent policy changes. *The Lancet* 2007; **369**(9578): 2042-9.
9. Corbett E, MacPherson P. Tuberculosis screening in high human immunodeficiency virus prevalence settings: turning promise into reality [State of the art series. Active case finding/screening. Number 5 in the series]. *The international journal of tuberculosis and lung disease* 2013; **17**(9): 1125-38.
10. Siddiqi K, Lambert M-L, Walley J. Clinical diagnosis of smear-negative pulmonary tuberculosis in low-income countries: the current evidence. *The Lancet infectious diseases* 2003; **3**(5): 288-96.
11. Ghana Health Service. Guidelines for the Clinical Management of TB and HIV Co-infection in Ghana. In: Department DCaP, editor. Accra; 2007.
12. Ministry of Health. Malawi National TB Programme Manual. . In: Programme NT, editor. 8 ed. Lilongwe; 2016.
13. National Department of Health. South Africa National Tuberculosis Management Guidelines 2014. In: Coordination TDS, editor. Pretoria; 2014.
14. Wilkinson D, Newman W, Reid A, Squire S, Sturm A, Gilks C. Trial-of-antibiotic algorithm for the diagnosis of tuberculosis in a district hospital in a developing country with high HIV prevalence. *The International Journal of Tuberculosis and Lung Disease* 2000; **4**(6): 513-8.
15. Whiting PF, Rutjes AW, Westwood ME, Mallett S, Deeks JJ, Reitsma JB, Leeflang MM, Sterne JA, Bossuyt PM. QUADAS-2: a revised tool for the quality assessment of diagnostic accuracy studies. *Annals of internal medicine* 2011; **155**(8): 529-36.
16. Rychetnik L, Frommer M, Hawe P, Shiell A. Criteria for evaluating evidence on public health interventions. *Journal of Epidemiology & Community Health* 2002; **56**(2): 119-27.
17. Schunemann HJ, Oxman AD, Brozek J, Glasziou P, Jaeschke R, Vist GE, Williams JW, Jr., Kunz R, Craig J, Montori VM, Bossuyt P, Guyatt GH, Group GW. Grading quality of evidence and strength of recommendations for diagnostic tests and strategies. *BMJ* 2008; **336**(7653): 1106-10.
18. Levy SB, Marshall B. Antibacterial resistance worldwide: causes, challenges and responses. *Nature medicine* 2004; **10**(12s): S122.
19. Goossens H, Ferech M, Vander Stichele R, Elseviers M, Group EP. Outpatient antibiotic use in Europe and association with resistance: a cross-national database study. *The Lancet* 2005; **365**(9459): 579-87.
20. Everett DB, Mukaka M, Denis B, Gordon SB, Carrol ED, van Oosterhout JJ, Molyneux EM, Molyneux M, French N, Heyderman RS. Ten years of surveillance for invasive Streptococcus pneumoniae during the era of antiretroviral scale-up and cotrimoxazole prophylaxis in Malawi. *PloS one* 2011; **6**(3): e17765.
21. Coles CL, Mabula K, Seidman JC, Levens J, Mkocha H, Munoz B, Mfinanga SG, West S. Mass distribution of azithromycin for trachoma control is associated with increased risk of

Approved by College of Medicine
13-Feb-2020
(COMREC) Research and Ethics Committee

azithromycin-resistant Streptococcus pneumoniae carriage in young children 6 months after treatment. *Clinical Infectious Diseases* 2013; **56**(11): 1519-26.
22. Mitjà O, Houinei W, Moses P, Kapa A, Paru R, Hays R, Lukehart S, Godornes C, Bieb SV, Grice T. Mass treatment with single-dose azithromycin for yaws. *New England Journal of Medicine* 2015; **372**(8): 703-10.
23. Maher MC, Alemayehu W, Lakew T, Gaynor BD, Haug S, Cevallos V, Keenan JD, Lietman TM, Porco TC. The fitness cost of antibiotic resistance in Streptococcus pneumoniae: insight from the field. *PLoS One* 2012; **7**(1): e29407.
24. Man WH, de Steenhuijsen Piters W, Bogaert D. The microbiota of the respiratory tract: gatekeeper to respiratory health. *Nat Rev Microbiol* 2017; **15**(5): 259-70.
25. Satzke C, Turner P, Virolainen-Julkunen A, Adrian PV, Antonio M, Hare KM, Henao-Restrepo AM, Leach AJ, Klugman KP, Porter BD. Standard method for detecting upper respiratory carriage of Streptococcus pneumoniae: updated recommendations from the World Health Organization Pneumococcal Carriage Working Group. *Vaccine* 2013; **32**(1): 165-79.
26. Cain KP, Anekthananon T, Burapat C, Akksilp S, Mankhatitham W, Srinak C, Nateniyom S, Sattayawuthipong W, Tasaneeyapan T, Varma JK. Causes of death in HIV-infected persons who have tuberculosis, Thailand. *Emerging infectious diseases* 2009; **15**(2): 258.
27. Bedell RA, Anderson ST, Van Lettow M, Åkesson A, Corbett EL, Kumwenda M, Chan AK, Heyderman RS, Zachariah R, Harries AD. High prevalence of tuberculosis and serious bloodstream infections in ambulatory individuals presenting for antiretroviral therapy in Malawi. *PLoS One* 2012; **7**(6): e39347.
28. Schleicher G, Feldman C. Dual infection with Streptococcus pneumoniae and Mycobacterium tuberculosis in HIV-seropositive patients with community acquired pneumonia. *The International Journal of Tuberculosis and Lung Disease* 2003; **7**(12): 1207-8.
29. Musicha P, Cornick JE, Bar-Zeev N, French N, Masesa C, Denis B, Kennedy N, Mallewa J, Gordon MA, Msefula CL, Heyderman RS, Everett DB, Feasey NA. Trends in antimicrobial resistance in bloodstream infection isolates at a large urban hospital in Malawi (1998–2016): a surveillance study. *The Lancet Infectious Diseases* 2017; **17**(10): 1042-52.
30. Nliwasa M, MacPherson P, Chisala P, Kamdolozi M, Khundi M, Kaswaswa K, Mwapasa M, Msefula C, Sohn H, Flach C. The sensitivity and specificity of Loop-Mediated Isothermal Amplification (LAMP) assay for Tuberculosis diagnosis in adults with chronic cough in Malawi. *PloS one* 2016; **11**(5): e0155101.
31. Lubell Y, Turner P, Ashley EA, White NJ. Susceptibility of bacterial isolates from community-acquired infections in sub-Saharan Africa and Asia to macrolide antibiotics. *Tropical Medicine & International Health* 2011; **16**(10): 1192-205.
32. Phua J, Dean NC, Guo Q, Kuan WS, Lim HF, Lim TK. Severe community-acquired pneumonia: timely management measures in the first 24 hours. *Critical Care* 2016; **20**(1): 237.
33. Lim W, Smith D, Wise M, Welham S. British Thoracic Society community acquired pneumonia guideline and the NICE pneumonia guideline: how they fit together. *Thorax* 2015: thoraxjnl-2015-206881.
34. van der Paardt A-F, Wilffert B, Akkerman OW, de Lange WC, van Soolingen D, Sinha B, van der Werf TS, Kosterink JG, Alffenaar J-WC. Evaluation of macrolides for possible use against multidrug-resistant Mycobacterium tuberculosis. *European Respiratory Journal* 2015; **46**(2): 444-55.
35. Falzari K, Zhu Z, Pan D, Liu H, Hongmanee P, Franzblau SG. In vitro and in vivo activities of macrolide derivatives against Mycobacterium tuberculosis. *Antimicrob Agents Chemother* 2005; **49**(4): 1447-54.
36. Fry A, Jha H, Lietman T, Chaudhary JP, Bhatta R, Elliott J, Hyde T, Schuchat A, Gaynor B, Dowell S. Adverse and beneficial secondary effects of mass treatment with azithromycin to eliminate blindness due to trachoma in Nepal. *Clinical infectious diseases* 2002; **35**(4): 395-402.
37. Porco TC, Gebre T, Ayele B, House J, Keenan J, Zhou Z, Hong KC, Stoller N, Ray KJ, Emerson P. Effect of mass distribution of azithromycin for trachoma control on overall mortality in Ethiopian children: a randomized trial. *Jama* 2009; **302**(9): 962-8.

Approved by
College of Medicine
13-Feb-2020
(COMREC)
Research and Ethics Committee

38. Keenan JD, Ayele B, Gebre T, Zerihun M, Zhou Z, House JI, Gaynor BD, Porco TC, Emerson PM, Lietman TM. Childhood mortality in a cohort treated with mass azithromycin for trachoma. *Clin Infect Dis* 2011; **52**(7): 883-8.
39. Musher DM, Thorner AR. Community-acquired pneumonia. *N Engl J Med* 2014; **371**(17): 1619-28.
40. Gordon SB, Chaponda M, Walsh AL, Whitty CJ, Gordon MA, Machili CE, Gilks CF, Boeree MJ, Kampondeni S, Read RC, Molyneux ME. Pneumococcal disease in HIV-infected Malawian adults: acute mortality and long-term survival. *AIDS* 2002; **16**(10): 1409-17.
41. Jochems SP, Weiser JN, Malley R, Ferreira DM. The immunological mechanisms that control pneumococcal carriage. *PLoS Pathog* 2017; **13**(12): e1006665.
42. Goldblatt D, Hussain M, Andrews N, Ashton L, Virta C, Melegaro A, Pebody R, George R, Soininen A, Edmunds J, Gay N, Kayhty H, Miller E. Antibody responses to nasopharyngeal carriage of Streptococcus pneumoniae in adults: a longitudinal household study. *J Infect Dis* 2005; **192**(3): 387-93.
43. National Tuberculosis Control programme. Malawi Nationwide Tuberculosis Prevalence Survey. 45th Union World Conference on Lung Health. Barcelona, Spain, 2014.
44. Global Health Innovative Technology Fund. Highly Sensitive POC TB-LAM Rapid Diagnostic Test. 2017. https://www.ghitfund.org/impact/portfolio/awpdetail/detail/78 (accessed 28 Feb 2018).
45. EuroQol G. EuroQol--a new facility for the measurement of health-related quality of life. *Health policy (Amsterdam, Netherlands)* 1990; **16**(3): 199.
46. Maheswaran H, Petrou S, MacPherson P, Choko AT, Kumwenda F, Lalloo DG, Clarke A, Corbett EL. Cost and quality of life analysis of HIV self-testing and facility-based HIV testing and counselling in Blantyre, Malawi. *BMC medicine* 2016; **14**(1): 34.
47. Jelsma J, Hansen K, De Weerdt W, De Cock P, Kind P. How do Zimbabweans value health states? *Population health metrics* 2003; **1**(1): 11.
48. Drummond MF, Sculpher MJ, Claxton K, Stoddart GL, Torrance GW. Methods for the economic evaluation of health care programmes: Oxford university press; 2015.
49. Maheswaran H, Petrou S, MacPherson P, Kumwenda F, Lalloo DG, Corbett EL, Clarke A. Economic costs and health-related quality of life outcomes of HIV treatment after self-and facility-based HIV testing in a cluster randomized trial. *Journal of acquired immune deficiency syndromes (1999)* 2017; **75**(3): 280.
50. Maheswaran H, Petrou S, Cohen D, MacPherson P, Kumwenda F, Lalloo DG, Corbett EL, Clarke A. Economic costs and health-related quality of life outcomes of hospitalised patients with high HIV prevalence: A prospective hospital cohort study in Malawi. *PLOS ONE* 2018; **13**(3): e0192991.
51. Frye JE. International Medical Products price indicator guide: Management Sciences for Health, 2015.

Approved by
College of Medicine
13-Feb-2020
(COMREC)
Research and Ethics Committee

18 Appendix 1: Informed consent

Included as a separate document on headed pages.

Approved by
College of Medicine
13-Feb-2020
(COMREC)
Research and Ethics Committee

19 Appendix 2: Division of Microbiology and Infectious Diseases (DMID) adult toxicity table

TABLE VERSION: November 2007

ABBREVIATIONS: Abbreviations utilized in the Table:

ULN = Upper Limit of Normal LLN = Lower Limit of Normal R_x = Therapy Req = Required

Mod = Moderate IV = Intravenous ADL = Activities of Daily Living Dec = Decreased

- **ESTIMATING SEVERITY GRADE**

For abnormalities NOT found elsewhere in the Toxicity Tables use the scale below to estimate grade of severity:

GRADE 1 **Mild** Transient or mild discomfort

(< 48 hours); no medical intervention/therapy required

GRADE 2 **Moderate** Mild to moderate limitation in activity - some assistance may be needed; no or minimal medical intervention/therapy required

GRADE 3 **Severe** Marked limitation in activity, some assistance usually required; medical intervention/therapy required, hospitalizations possible

GRADE 4 **Life-threatening** Extreme limitation in activity, significant assistance required; significant medical intervention/therapy required, hospitalization or hospice care probable

SERIOUS OR LIFE-THREATENING AEs

ANY clinical event deemed by the clinician to be serious or life-threatening should be considered a grade 4 event. Clinical events considered to be serious or life-threatening include, but are not limited to: seizures, coma, tetany, diabetic ketoacidosis, disseminated intravascular coagulation, diffuse petechiae, paralysis, acute psychosis, severe depression.

COMMENTS REGARDING THE USE OF THIS TABLE

- Standardized and commonly used toxicity tables (Division of AIDS, NCI's Common Toxicity Criteria (CTC), and World Health Organization (WHO)) have been adapted for use by the Division of Microbiology and Infectious Diseases (DMID) and modified to better meet the needs of participants in DMID trials.
- For parameters not included in the following Toxicity Tables, sites should refer to the "Guide for Estimating Severity Grade" located above.
- Criteria are generally grouped by body system.
- Some protocols may have additional protocol specific grading criteria, which will supersede the use of these tables for specified criteria.

Approved by
College of Medicine
13-Feb-2020
(COMREC)
Research and Ethics Committee

HEMATOLOGY				
	Grade 1	Grade 2	Grade 3	Grade 4
Hemoglobin	9.5 - 10.5 gm/d L	8.0 - 9.4gm/dL	6.5 - 7.9 gm/d L	< 6.5 gm/dL
Absolute Neutrophil Count	1000-1500/ mm^3	750-999/ mm^3	500-749/ mm^3	<500/ mm^3
Platelets	75,000-<br>99,999/ mm^3	50,000-<br>74,999/ mm^3	20,000-49,999/<br>mm^3	<20,000/ mm^3
WBCs	11,000-13,000/<br>mm^3	13,000-<br>15,000 / mm^3	15,000-<br>30,000/ mm^3	>30,000 or<br><1,000 / mm^3
% Polymorphonuclear<br>Leucocytes + Band Cells	> 80%	90 – 95%	>95%	----------
Abnormal Fibrinogen	Low:<br>100-200 mg/dL<br>High:<br>400-600 mg/dL	Low:<br><100 mg/dL<br>High:<br>>600 mg/dL	Low:<br>< 50 mg/dL<br>----------	Fibrinogen<br>associated with<br>gross bleeding<br>or with<br>disseminated<br>coagulation
Fibrin Split Product	20-40 mcg/ ml	41-50 mcg/ ml	51-60 mcg/ ml	> 60 mcg/ ml
Prothrombin Time (PT)	1.01 - 1.25 x ULN	1.26-1.5 x ULN	1.51 -3.0 x ULN	>3 x ULN
Activated Partial<br>Thromboplastin (APPT)	1.01 -1.66 x ULN	1.67 - 2.33 x<br>ULN	2.34 - 3 x ULN	> 3 x ULN
Methemoglobin	5.0 - 9.9 %	10.0 - 14.9 %	15.0 - 19.9%	> 20.0 %

Approved by
College of Medicine
13-Feb-2020
(COMREC)
Research and Ethics Committee

CHEMISTRIES				
	Grade 1	Grade 2	Grade 3	Grade 4
Hyponatremia	130-135 mEq/ L	123-129 mEq/ L	116-122 mEq/ L	< 116 mEq/ L or abnormal sodium *with* mental status changes or seizures
Hypernatremia	146-150 mEq/ L	151-157 mEq/ L	158-165 mEq/ L	> 165 mEq/ L or abnormal sodium *with* mental status changes or seizures
Hypokalemia	3.0 - 3.4 mEq/ L	2.5 - 2.9 mEq/ L	2.0 - 2.4 mEq/ L or intensive replacement therapy or hospitalization required	< 2.0 mEq/ L or abnormal potassium *with* paresis, ileus or life-threatening arrhythmia
Hyperkalemia	5.6 - 6.0 mEq/ L	6.1 - 6.5 mEq/ L	6.6 - 7.0 mEq/l	> 7.0 mEq/ L or abnormal potassium *with* life-threatening arrhythmia
Hypoglycemia	55-64 mg/dL	40-54 mg/dL	30-39 mg/dL	<30 mg/d L or abnormal glucose *with* mental

Approved by
College of Medicine
Page 69 of 79
13-Feb-2020
(COMREC)
Research and Ethics Committee

				status changes or coma
Hyperglycemia (nonfasting and no prior diabetes)	116 - 160 mg/dL	161- 250 mg/d L	251 - 500 mg/dL	> 500 mg/d L or abnormal glucose *with* ketoacidosis or seizures
Hypocalcemia (corrected for albumin)	8.4 - 7.8 mg/dL	7.7 - 7.0 mg/dL	6.9 - 6.1 mg/dL	< 6.1 mg/dL or abnormal calcium *with* life threatening arrhythmia or tetany
Hypercalcemia (correct for albumin)	10.6 - 11.5 mg/d L	11.6 - 12.5 mg/d L	12.6 - 13.5 mg/d L	> 13.5 mg/dL or abnormal calcium *with* life threatening arrhythmia
Hypomagnesemia	1.4 - 1.2 mEq/ L	1.1 - 0.9 mEq/ L	0.8 - 0.6 mEq/ L	< 0.6 mEq/ L or abnormal magnesium *with* life-threatening arrhythmia
Hypophosphatemia	2.0 - 2.4 mg/dL	1.5 -1.9 mg/dL or replacement Rx required	1.0 -1.4 mg/dL intensive therapy or hospitalization required	< 1.0 mg/dL or abnormal phosphate *with* life-threatening arrhythmia
Hyperbilirubinemia (when accompanied by any	1.1 - <1.25 x ULN	1.25 - <1.5 x ULN	1.5 – 1.75 x ULN	> 1.75 x ULN

Approved by
College of Medicine
Page 70 of 79
13-Feb-2020
(COMREC)
Research and Ethics Committee

increase in other liver function test)				
Hyperbilirubinemia (when other liver function are in the normal range)	1.1 - <1.5 x ULN	1.5 - <2.0 x ULN	2.0 – 3.0 x ULN	> 3.0 x ULN
BUN	1.25 - 2.5 x ULN	2.6 - 5 x ULN	5.1 - 10 x ULN	> 10 x ULN
Hyperuricemia (uric acid)	7.5 – 10.0 mg/dL	10.1 – 12.0 mg/d L	12.1 – 15.0 mg/d L	>15.0 mg/d L
Creatinine	1.1 - 1.5 x ULN	1.6 - 3.0 x ULN	3.1 - 6 x ULN	> 6 x ULN or dialysis required
**EN ZYMES**				
	Grade 1	Grade 2	Grade 3	Grade 4
AST (SGOT)	1.1 - <2.0 x ULN	2.0 – <3.0 x ULN	3.0 – 8.0 x ULN	> 8 x ULN
ALT (SGPT)	1.1 - <2.0 x ULN	2.0 – <3.0 x ULN	3.0 – 8.0 x ULN	> 8 x ULN
GGT	1.1 - <2.0 x ULN	2.0 – <3.0 x ULN	3.0 – 8.0 x ULN	> 8 x ULN
Alkaline Phosphatase	1.1 - <2.0 x ULN	2.0 – <3.0 x ULN	3.0 – 8.0 x ULN	> 8 x ULN
Amylase	1.1 - 1.5 x ULN	1.6 - 2.0 x ULN	2.1 - 5.0 x ULN	> 5.1 x ULN
Lipase	1.1 - 1.5 x ULN	1.6 - 2.0 x ULN	2.1 - 5.0 x ULN	> 5.1 x ULN
**URINALYSIS**				
	Grade 1	Grade 2	Grade 3	Grade 4
Proteinuria	1+ or 200 mg - 1 gm loss/day	2-3+ or 1- 2 gm loss/day	4+ or 2-3.5 gm loss/day	nephrotic syndrome or > 3.5 gm loss/day

Approved by
College of Medicine
Page 71 of 79
13-Feb-2020
(COMREC)
Research and Ethics Committee

Hematuria	microscopic only<br><10 rbc/hpf	gross, no clots<br>>10 rbc/hpf	gross, with or without clots, OR red blood cell casts	obstructive or required<br>transfusion
**CARDIOVASCULAR**				
	Grade 1	Grade 2	Grade 3	Grade 4
Cardiac Rhythm		asymptomatic,<br>transient signs, no<br>Rx required	recurrent/persistent;<br>symptomatic Rx required	unstable<br>dysrythmia; hospitalization<br>and treatment<br>required
Hypertension	transient increase<br>> 20 mm/ Hg; no treatment	recurrent, chronic increase<br>> 20mm/ Hg.<br>/treatment required	acute treatment<br>required; outpatient treatment or<br>hospitalization<br>possible	end organ<br>damage or hospitalization<br>required
Hypotension	transient orthostatic<br>hypotension with heart rate increased by <20 beat/min or decreased by <10 mm Hg systolic BP, No treatment required	symptoms due to orthostatic hypotension or BP decreased by <20 mm Hg systolic; correctable with oral flu id treatment	requires IV fluids; no hospitalization required	mean arterial pressure<br><60mm/ Hg or end organ damage or shock; requires hospitalization and vasopressor treatment

Approved by
College of Medicine
Page 72 of 79
13-Feb-2020
(COMREC)
Research and Ethics Committee

Pericarditis	minimal effusion	mild/ moderate asymptomatic effusion, no treatment	symptomatic effusion; pain; EKG changes	tamponade; pericardiocentesis or surgery required
Hemorrhage, Blood Loss	microscopic/occult	mild, no transfusion	gross blood loss; 1-2 units transfused	massive blood loss; > 3 units transfused
**RESPIRATORY**				
	Grade 1	Grade 2	Grade 3	Grade 4
Cough	transient- no treatment	persistent cough; treatment responsive	Paroxysmal cough; uncontrolled with treatment	--------------------
Bronchospasm, Acute	transient; no treatment; 70% - 80% FEV_1 of peak flow	requires treatment; normalizes with bronchodilator; FEV_1 50% - 70% (of peak flow)	no normalization with bronchodilator; FEV_1 25% - 50% of peak flow; or retractions present	cyanosis: FEV_1 < 25% of peak flow or intubation necessary
Dyspnea	dyspnea on exertion	dyspnea with normal activity	dyspnea at rest	dyspnea requiring Oxygen therapy
**GASTROINTESTINAL**				
	Grade 1	Grade 2	Grade 3	Grade 4

Approved by College of Medicine
Page 73 of 79
13-Feb-2020
(COMREC)
Research and Ethics Committee

Nausea	mild or transient; maintains reasonable intake	moderate discomfort; intake decreased significantly; some activity limited	no significant intake; requires IV flu ids	hospitalization required;
Vomiting	1 episode in 24 hours	2-5 episodes in 24 hours	>6 episodes in 24 hours or needing IV fluids	physiologic consequences requiring hospitalization or requiring parenteral nutrition
Constipation	requiring stool softener or dietary modification	requiring laxatives	obstipation requiring manual evacuation or enema	obstruction or toxic megacolon
Diarrhea	mild or transient; 3-4 loose stools/Day-or mild diarrhea last < 1 week	moderate or persistent; 5-7 loose stools/Day-or diarrhea lasting >1 week	>7 loose stools/day or bloody diarrhea; or orthostatic hypotension or electrolyte imbalance or >2L IV fluids required	hypotensive shock or physiologic consequences requiring hospitalization
Oral Discomfort/Dysphagia	mild discomfort; no difficulty swallowing	some limits on eating/drinking	eating/talking very limited; unable to swallow solid foods	unable to drink flu ids; requires IV fluids
**NEUROLOGICAL**				

Approved by
College of Medicine
Page 74 of 79
13-Feb-2020
(COMREC)
Research and Ethics Committee

	Grade 1	Grade 2	Grade 3	Grade 4
Neuro-Cerebellar	slight incoordination dysdiadochokinesis	intention tremor, dysmetria, slurred speech; nystagmus	locomotor ataxia	incapacitated
Psychiatric	mild anxiety or depression	moderate anxiety or depression; therapy required; change in normal routine	severe mood changes requiring therapy; or suicidal ideation; or aggressive ideation	acute psychosis requiring hospitalization; or suicidal gesture/attempt or hallucinations
Muscle Strength	subjective weakness no objective symptoms/ signs	mild objective signs/symptoms no decrease in function	objective weakness function limited	paralysis
Paresthesia (burning, tingling, etc.)	mild discomfort; no treatment required	moderate discomfort; non-narcotic analgesia required	severe discomfort; or narcotic analgesia required with symptomatic improvement	incapacitating; or not responsive to narcotic analgesia
Neuro-sensory	mild impairment in sensation (decreased sensation, e.g., vibratory, pinprick, hot/cold in great toes) in	moderate impairment (mod decreased sensation, e.g., vibratory, pinprick, hot/cold to	severe impairment (decreased or loss of sensation to knees or wrists) or loss of sensation of at	sensory loss involves limbs and trunk; paralysis; or seizures

Approved by
College of Medicine
Page 75 of 79
13-Feb-2020
(COMREC)
Research and Ethics Committee

	focal area or symmetrical distribution; or change in taste, smell, vision and/or hearing	ankles) and/or joint position or mild impairment that is not symmetrical	least mod degree in multiple different body areas (i.e., upper and lower e xtremities)	
**MUSCULOSKELATEL**				
	Grade 1	Grade 2	Grade 3	Grade 4
Arthralgia (joint pain)	mild pain not interfering with function	moderate pain, analgesics and/or pain interfering with function but not with activities of daily living	severe pain; pain and/or analgesics interfering with activities of daily living	disabling pain
Arthritis	mild pain with inflammation, erythema or joint swelling – but not interfering with function	moderate pain with inflammation, erythema or joint swelling – interfering with function, but not with activities of daily living	severe pain with inflammation, erythema or joint swelling –and interfering with activities of daily living	permanent and/or disabling joint distruction
Myalgia	myalgia with no	muscle	severe muscle	frank

Approved by
College of Medicine
Page 76 of 79
13-Feb-2020
(COMREC)
Research and Ethics Committee

	limitation of activity	tenderness (at other than injection site) or with moderate impairment of activity	tenderness with marked impairment of activity	myonecrosis
**SKIN**				
	Grade 1	Grade 2	Grade 3	Grade 4
Mucocutaneous	erythema; pruritus	diffuse, maculo-papular rash, dry desquamation	vesiculation or moist desquamation or ulceration	exfoliative dermatitis, mucous membrane involvement or erythema, multiforme or suspected Stevens-Johnson or necrosis requiring surgery
Induration	< 15mm	15-30 mm	>30mm	
Erythema	< 15mm	15-30 mm	>30mm	
Edema	< 15mm	15-30 mm	>30mm	

Approved by College of Medicine
Page 77 of 79
13-Feb-2020
(COMREC)
Research and Ethics Committee

Rash at Injection Site	< 15mm	15-30 mm	>30mm	
Pruritus	slight itching at injection site	moderate itching at injection extremity	itching over entire body	
**SYSTEMIC**				
	Grade 1	Grade 2	Grade 3	Grade 4
Allergic Reaction	pruritus without rash	localized urticaria	generalized urticaria; angioedema	anaphylaxis
Headache	mild, no treatment required	transient, moderate; treatment required	severe; responds to initial narcotic therapy	intractable; requires repeated narcotic therapy
Fever: oral	37.7 - 38.5 C or 100.0 - 101.5 F	38.6 - 39.5 C or 101.6 - 102.9 F	39.6 - 40.5 C or 103 - 105 F	> 40 C or > 105 F
Fatigue	normal activity reduced < 48 hours	normal activity decreased 25-50% > 48 hours	normal activity decreased > 50% can't work	unable to care for self

Approved by
College of Medicine
Page 78 of 79
13-Feb-2020
(COMREC)
Research and Ethics Committee

20 Appendix 3: Package insert for Azithromycin and amoxicillin

To be included during ethics submission.

Approved by
College of Medicine
Page 79 of 79
13-Feb-2020
(COMREC)
Research and Ethics Committee

Trial Statistical Analysis Plan

Accuracy and Consequences of using Trial-of-antibiotics for TB diagnosis (ACT-TB Study)

Statistical Analysis Plan

<table>
<tr><td>Full Title</td><td colspan="3">Randomised controlled clinical trial investigating benefits of using response to broad spectrum antibiotics as an exclusion diagnostic for tuberculosis (TB) in primary care adult patients versus risk of antimicrobial resistance (AMR)</td></tr>
<tr><td>Acronym</td><td colspan="3">Accuracy and Consequences of using Trial-of-antibiotics for TB diagnosis (ACT-TB Study)</td></tr>
<tr><td rowspan="6">Document History</td><td>Version No.</td><td>Version Date</td><td>Description of Change</td></tr>
<tr><td>1.0</td><td>20 May 2020</td><td>Initial release</td></tr>
<tr><td></td><td></td><td></td></tr>
<tr><td></td><td></td><td></td></tr>
<tr><td></td><td></td><td></td></tr>
<tr><td></td><td></td><td></td></tr>
<tr><td>Protocol</td><td colspan="3">Version 4.0, 27 Jan 2020</td></tr>
<tr><td>Trial Registration</td><td colspan="3">ClinicalTrials.gov (NCT03545373)</td></tr>
<tr><td>Principal Investigators</td><td colspan="3">Titus Divala</td></tr>
<tr><td>SAP Authors</td><td colspan="3">Titus Divala, Elizabeth Corbett and Katherine Fielding</td></tr>
</table>

Table of Contents

1 Introduction 4

2 Study design summary 4

3 General approach for outcome analysis 7

4 Sample size justification 7

4.1 Diagnostic impact outcome 7

4.1.1 Sample size for a combination of 2 antibiotic arms against standard of care arm 7

4.1.2 Sample size for one antibiotic arm against standard of care arm 8

4.2 Power for clinical impact outcome 9

4.3 Power for AMR outcome 9

5 General definitions for analysis 10

5.1 Study period and visit definitions 10

5.2 Study populations 11

5.3 Subgroup definitions 11

5.4 Treatment assignment and treatment arms 11

6 Study patients 11

6.1 Patient flow 11

6.2 Inclusion and exclusion criteria 12

6.2.1 Inclusion criteria: 12

6.2.2 Exclusion criteria 12

6.3 Incomplete follow up 12

7 Demographics and baseline characteristics 12

8 Diagnostic impact of trial of antibiotics 13

8.1 Definitions for investigational test 13

8.2 Definitions for reference standard test 13

8.2.1 Reference standard definition for Primary outcome 1 13

8.2.2 Reference standard definition for Secondary Outcome 2 13

8.3 Diagnostic assessment outcome 13

8.4 Analysis for assessing diagnostic impact of trial-of-antibiotics 14

9 Clinical impact of trial-of-antibiotics 14

9.1 Outcome definitions 15

9.2 Analysis for assessing clinical impact of trial-of-antibiotics 15

10 Impact of trial-of-antibiotics on antimicrobial resistance (AMR) 15

10.1 Outcome definitions 15

10.2 Analysis for assessing clinical impact of trial-of-antibiotics 16

11 APPENDIX 1: Study Consort Diagram 17

12 APPENDIX 2: Participant characteristics at baseline 18

13 APPENDIX 3: Results for primary outcomes 19

14 APPENDIX 4: Results for secondary outcomes 21

15 APPENDIX 5: Diagnostic performance of ACASI-reported change in symptoms against a sputum TB diagnostic reference 22

1 Introduction

The present document comprises the Statistical Analysis Plan (SAP) for **A**ccuracy and **C**onsequences of using **T**rial-of-antibiotics for **TB** diagnosis (ACT-TB Study), a trial investigating benefits and risks of using response to broad spectrum antibiotics as an exclusion diagnostic for tuberculosis (TB) in primary care adult patients.

Scoping statement

The SAP contains:

- details of the planned statistical analyses associated with a clinical study so that the analyses are planned with the desired work product(s) in mind and can be conducted in a consistent, repeatable manner.
- detailed requirements and parameters for the reporting database, statistical programs/output reports, and any tests of the robustness and sensitivity of the analysis.
- example tables, figures and listings.

2 Study design summary

**Title**	Randomised controlled clinical trial investigating benefits of using response to broad spectrum antibiotics as an exclusion diagnostic for tuberculosis (TB) in primary care adult patients versus risk of antimicrobial resistance (AMR)
**Design**	Three arm (625 per arm) individually randomised (1:1:1), open-label controlled clinical trial investigating standard care diagnostic approach for tuberculosis. The trial will not use any unlicensed products.

**Objective**	**Outcomes**
**Primary**	
1. To establish the diagnostic value of trial-of-antibiotics for excluding pulmonary tuberculosis (PTB) in adults with cough (and have a valid sputum test result) at primary care level in Malawi.	Proportion of participants correctly classified as PTB negative based on report of improvement of baseline symptoms on study Day-8 (i.e. after a trial-of-antibiotics if in azithromycin or amoxicillin arms, or without antibiotics if in standard of care arm) against a mycobacteriology reference standard, among participants with a valid result from at least one sputum TB test
2. To determine the overall clinical benefit of giving empirical antibiotic treatment in primary care participants with chronic cough.	Proportion of participants experiencing at least one of the following adverse outcomes by Day 29:<br>1) death<br>2) hospitalisation

	3) missed TB diagnosis
**Secondary**	
3 To evaluate using nasopharyngeal Streptococcus pneumonia, the effect of a trial-of-antibiotics on selection for antimicrobial resistance.	Proportion of day 29 nasopharyngeal *Streptococcus pneumoniae* isolates resistant to commonly used antimicrobials.
4. To establish the diagnostic value of trial-of-antibiotics for excluding pulmonary tuberculosis (PTB) in primary care presenting Malawian adults with cough including those unable to produce sputum.	Proportion of participants correctly classified as PTB negative based on report of improvement of baseline symptoms on study Day-8 (i.e. after a trial-of-antibiotics if in azithromycin or amoxicillin arms, or without antibiotics if in standard of care arm) against a mycobacteriology reference standard, among all randomised participants, with those who could not provide sputum classified as mycobacteriologically negative.
5. To estimate the incremental cost-effectiveness of trial-of-antibiotics using azithromycin and trial-of-antibiotics using amoxicillin in comparison to standard of care, and to each other.<br><br>**The SAP does not cover outcomes under this objective.**	• Incremental cost per quality adjusted life year gained<br>• Total direct medical costs per participant over 56 days<br>• Eq-5D utility score
**Exploratory**	
Our exploratory analyses will be comparisons between the azithromycin and amoxicillin arms for all our primary and secondary outcomes.	

**Population**	Adults presenting to primary care centres in Malawi reporting cough.
	Inclusion criteria:<br>• Ambulatory clinic attendees presenting with cough<br>• Should have been ill for ≥ 14 days<br>• Aged at least 18 years<br>• Reside in Blantyre and willing to return to the same clinic for follow up visits over the entire study period.<br><br>Exclusion criteria:<br>• Self-reported allergy to study medications<br>• Acute danger signs defined in national TB program treatment guidelines<br>• Tuberculosis treatment or isoniazid preventive therapy in the last 6 months<br>• Treated with antibiotics, other than co-trimoxazole prophylaxis, for the current illness or within the past 14 days
**Treatment**	**Arm 1**: Azithromycin 500mg once daily for 3 days commencing on randomization day.

	**Arm 2**: Amoxicillin 1 g 3 times daily for 5 days commencing on randomization day.<br><br>**Arm 3**: Standard of care in current national guidelines for patients presenting with cough and without danger signs (No treatment until re-evaluation with sputum TB test results)
**Duration**	Antibiotics will be prescribed on the randomisation day (Day 1) and follow up activities performed on days 8 and 29.

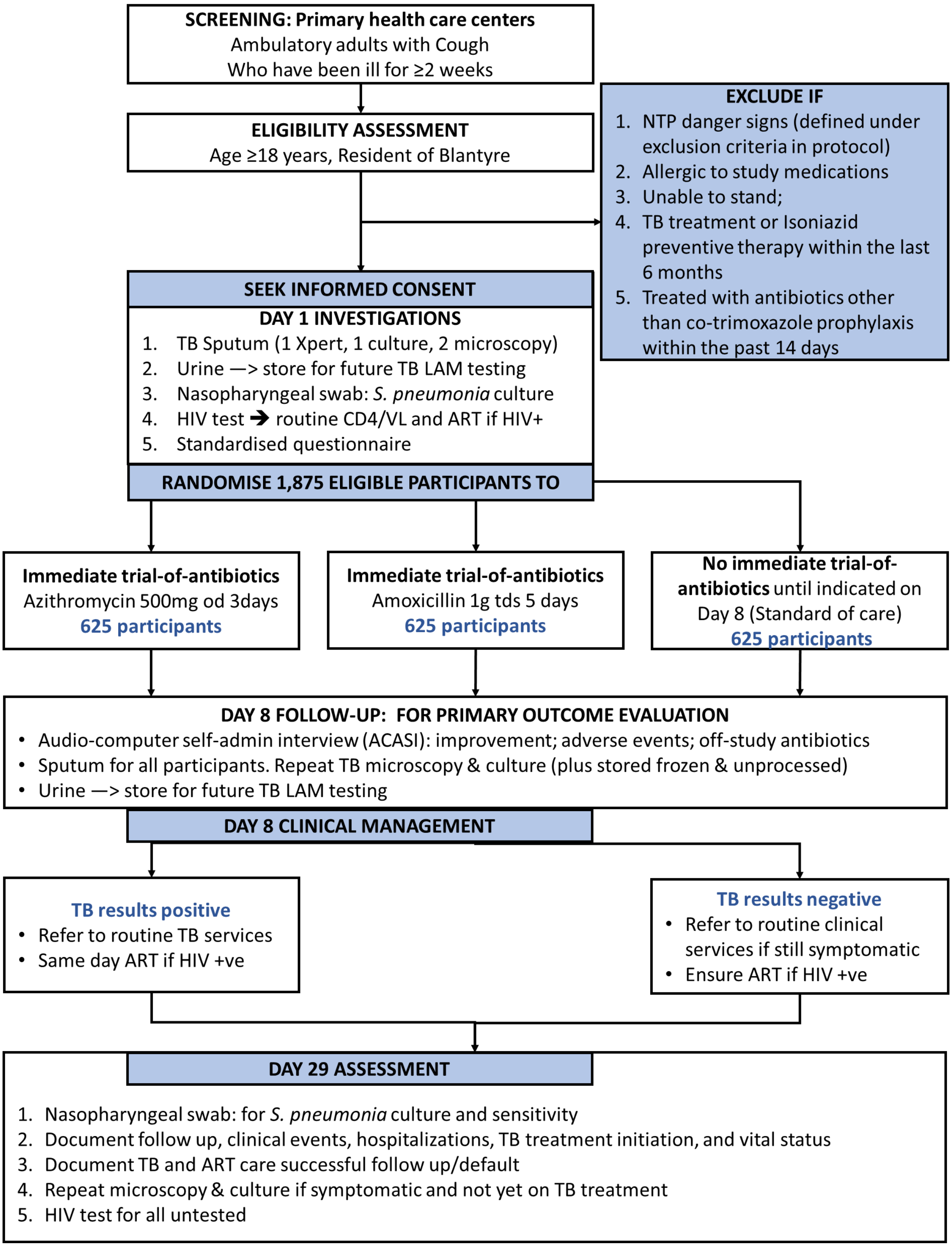

Figure 1: Flow diagram for the planned clinical trial in Blantyre, Malawi

3 General approach for outcome analysis

Study outcome definitions are expanded in sections 8, 9 and 10. For all outcomes, we will perform analyses and report measures of effect from the following comparisons:

a. azithromycin or amoxicillin (combined) versus standard of care

b. azithromycin versus standard of care

c. amoxicillin versus standard of care

d. Exploratory analysis: azithromycin vs amoxicillin

All analyses will be adjusted for randomisation strata of primary care centre (two strata). The main study results will be based on comparison a. Comparison d is exploratory. We will perform analyses b and c without accounting for multiple comparisons. Imbalances at baseline are unlikely, though will assessed without the use of hypothesis testing. Any major imbalances will be adjusted for.

4 Sample size justification

We performed power and sample size estimations for the diagnostic impact, clinical impact, and AMR impact outcomes as follows:

4.1 Diagnostic impact outcome

We assume that at Day 8, change in well-being from baseline state in trial-of-antibiotics (azithromycin or amoxicillin) arms will correctly classify 60% of all mycobacteriology negative participants (i.e 60%specificity in trial-of-antibiotics arms). [12] We wanted to estimate a sample size that would provide a discriminatory power of 80% at a two-sided significance level of 5%, to detect at least 10% difference in specificity (i.e ≤50% specificity in standard of care arm). The sample sizes will differ by the number of arms being compared, we have therefore provided two separate estimates in line with type of comparisons specified under section 7.

4.1.1 Sample size for a combination of 2 antibiotic arms against standard of care arm

The sample size estimates along with assumptions for this comparison are shown in the Table 1A. To achieve the desired 80% discriminatory power, we will need to recruit at least 305 sputum-test-negative participants per arm. Accounting for TB prevalence, ability to

produce and submit sputum, and loss-to-follow up increases the sample to 472 per arm or 1,416 for the whole study.

Table 1A: Sample size estimation for the *diagnostic impact outcome* comparing a combination of two antibiotic arms to standard of care arm

**POWER** (X2 difference between independent proportions**)**	**Effect size (**50% SoC vs 60% amoxycillin or azithromycin**)**	**Effective sample per arm (**Sputum negative**)**	**Include sputum positive** (20%**)**	**Include inability to submit sputum** (15%**) and LTFUP** (5%**)**	**Total sample size (**all three arms**)**
0.60	0.10	262	328	409	1,228
0.65	0.10	292	365	456	1,369
0.70	0.10	325	406	508	1,523
0.75	0.10	363	454	567	1,702
0.80	0.10	400	500	625	1,875
0.85	0.10	463	579	723	2,170
0.90	0.10	538	673	841	2,522
0.95	0.10	661	826	1,033	3,098

Target power and respective sample size estimates based on knowledge of TB risk, ability to produce and submit sputum, and loss-to-follow up.

4.1.2 Sample size for one antibiotic arm against standard of care arm

The sample size estimates along with assumptions for this comparison are shown in the Table 1B. To achieve the desired 80% discriminatory power, we will need to recruit at least 400 sputum-test-negative participants per arm. Accounting for TB prevalence, ability to produce and submit sputum, and loss-to-follow up increases the sample to 625 per arm or 1,875 for the whole study.

Table 1B: Sample size estimation for the *diagnostic impact outcome* one antibiotic arm to standard of care arm

**POWER** (X2 difference between independent proportions**)**	**Effect size (**50% SoC vs 60% amoxycillin or azithromycin**)**	**Effective sample per arm (**Sputum negative**)**	**Include sputum positive** (20%**)**	**Include inability to submit sputum** (15%**) and LTFUP** (5%**)**	**Total sample size (**all three arms**)**

0.60	0.10	196	245	306	919
0.65	0.10	218	273	341	1,022
0.70	0.10	243	304	380	1,139
0.75	0.10	271	339	423	1,270
0.80	0.10	305	381	477	1,430
0.85	0.10	347	434	542	1,627
0.90	0.10	403	504	630	1,889
0.95	0.10	495	619	773	2,320

Target power and respective sample size estimates based on knowledge of TB risk, ability to produce and submit sputum, and loss-to-follow up.

4.2 Power for clinical impact outcome

For the clinical impact of trial-of-antibiotics outcome, we assume a 4% baseline risk of composite outcome, and a loss to follow up of 10% by Day 29. Using the sample size of 625 participants per arm (obtained in Table 2B), and a type I alpha of 5%, we will be able to detect the difference between arms with 80% power, if the risk in the intervention arm is twice that of the standard of care arm. This estimate is applicable to all comparisons a, b and c shown in section 3.

4.3 Power for AMR outcome

Study arms will be compared based proportion of participants with resistant *Streptococcus pneumoniae* on day 29. We assume that 45% of Day-29 nasopharyngeal swabs will successfully grow *Streptococcus pneumoniae,* and that 10% of the isolates will meet the definition of resistance (described earlier under outcomes), and that 10% will be lost to follow up by Day 29. Therefore, on day 29, the standard of care arm (of 625 participants) will have 253 *Streptococcus pneumoniae* isolates, 25 of which would meet the definition of resistance. This translates into a 4% (25/625) risk of AMR positive cases in the standard of care arm. To detect a twofold change in odds of day 29 AMR risk with at least 80% power, using Pearson's Chi-squared test, at 0.05 alpha, we will need at least 431 and 553 participants per arm for the 2:1 and pairwise comparisons respectively.

5 General definitions for analysis

5.1 Study period and visit definitions

Recruitment of patients started on 25 March 2019 and will complete in or after March 2020. Following randomisation (day 1), each participant is expected to attend follow up visits at day 8 (+/-3 days) and day 29 (+3 weeks). Table 1 shows study activities planned for each study visit.

Table 1: Activities over the study period

	STUDY PERIOD			
	Enrolment	Follow up		
TIMEPOINT	Day 1	Day 8		Day 29
ENROLMENT:				
Eligibility screen	x			
Informed consent	x			
Randomisation	x			
INTERVENTIONS:				
Azithromycin	x			
Amoxicillin	x			
Standard of care	x			
ASSESSMENTS:				
Demographics	x			
History of antibiotic use	x	x		x
History & examination	x	x		x
TB symptoms	x	x		x
Sputum collection[1]	x	x		[x]
Urine for TB LAM test[2]	x	x		
Nasopharyngeal swab for AMR[3]	x			x
HIV test	x			
Linking to routine care	x	x		x
ACASI[4]		x		
Clinical events[5]				x

Update contact & address		x		x

1. On Day 29 collect sputum and perform mycobacteriology if the participant is symptomatic
2. Urine sample collected for mycobacterial lipoarabinomannan when Fujifilm SILVAMP TB LAMtest becomes available
3. Nasopharyngeal swab for Streptococcus pneumoniae culture and sensitivity as a way of determining risk of antimicrobial resistance (AMR)
4. Audio Computer Assisted Self-Interview (ACASI) for documenting change of symptoms on Day 8 versus Day 1
5. Illnesses, clinic visits, radiological outcomes, new HIV diagnosis, new tuberculosis diagnosis, death, hospitalisation, missed tuberculosis diagnosis

5.2 Study populations

Patients are considered randomized when they are assigned a randomization number. All analyses will follow the intention to treat principle. Each participant will be analysed based on the treatment arm they were allocated to regardless of adherence to study protocol. Analysis for the primary outcome 1 (proportion of sputum-test-negative participants who are ACASI-test-negative) will be restricted to participants who have at least one valid sputum-test-result.

5.3 Subgroup definitions

We will perform sub-group analyses for diagnostic accuracy and clinical benefit outcomes for the following variables; i) HIV status (positive, negative or unknown), ii) antiretroviral treatment (started/not started) as documented on enrolment day.

5.4 Treatment assignment and treatment arms

Randomization lists, stratified by study site (primary care centre), with variable block sizes and indicating a randomization number and which treatment is to be given, were produced prior to the start of the trial by a London based statistician not affiliated with the study. The code for each individual was provided in separate sealed envelopes and assigned to individuals in the order in which they are being enrolled in the study. Management of patients is not blinded due to the cost of arranging placebo. However, study team masking will be maintained with all study outcome assessment occurring without reference to randomisation arm.

6 Study patients

6.1 Patient flow

A clear accounting of all patients who entered the study, using figures and/or tables will be presented. Numbers of patients screened, randomized, and completed each phase of the study as well as reasons for withdrawals presented by treatment arm using a Consolidated Standards of Reporting Trials (CONSORT) flow chart (appendix 1). This is a generic flow chart and will be repeated for all scenarios for the primary outcome.

6.2 Inclusion and exclusion criteria

Inclusion and exclusion criteria are as follows:

6.2.1 Inclusion criteria:

To be eligible for inclusion in this trial, patients have to fulfil all of the following criteria:

- Ambulatory clinic attendees presenting with cough
- Unwell for at least 14 days
- Aged at least 18 years

Reside in Blantyre and willing to return to the same clinic for follow up visits over the entire study period.

6.2.2 Exclusion criteria

Patients who meet any of the following criteria will not be eligible for the study.

- Self-reported allergy to study medications
- WHO/Malawi National tuberculosis Program (NTP) danger signs: respiratory rate > 30/min, temperature >39°C, Heart rate >120/minute, confused/agitated, respiratory distress, systolic blood pressure <90 mmHg, inability to walk unassisted
- Treated with antibiotics other than co-trimoxazole prophylaxis within the past 14 days
- Tuberculosis treatment or isoniazid preventive therapy within the last 6 months

6.3 Incomplete follow up

Frequency of withdrawals and loss to follow up will be summarised by treatment arm along with respective reasons.

7 Demographics and baseline characteristics

Demographic and baseline clinical characteristics of the randomized patients will be summarised by treatment arm (see appendix 2 for table shells). The distribution of categorical variables will be summarised by percentages. Quantitative variables will be summarised using the mean and standard deviation (SD) or median and inter-quartile range (IQR), where appropriate, and the minimum and maximum and sample size of non-missing data. We will present demographic and baseline clinical characteristics for intention-to-treat and diagnostic assessment populations.

8 Diagnostic impact of trial of antibiotics

Primary and secondary diagnostic impact analyses will be based on the investigational and reference tests as defined in the following sections.

8.1 Definitions for investigational test

The investigational test is change in symptoms at Day 8 categorised as: improved or not improved (no change or worsened) in response to the following question delivered to patients using Audio Computer Assisted Self-Interview (ACASI): *on day 1, you reported that you were unwell; compared to that day, has your illness worsened, remained the same, or improved?*

We will term ACASI interview outcome as

- **ACASI-test-negative,** if the participant reports improvement (i.e TB ruled out)
- **ACASI-test-positive,** if the participant reports no change or worsening (i.e TB likely)

8.2 Definitions for reference standard test

The reference standard test will be defined based on sputum mycobacteriology test result on days 1 and 8. The interpretation of the reference standard will be as follows for each diagnostic impact outcome:

8.2.1 Reference standard definition for Primary outcome 1

- **Sputum-test-positive,** if there is at least one positive of smear microscopy, Xpert/MTB/RIF, or MTB culture;
- **Sputum-test-negative,** if none of the tests are positive AND at least one test is known to be negative.

8.2.2 Reference standard definition for Secondary Outcome 2

- **Sputum-test-positive,** if there is at least one positive of smear microscopy, Xpert/MTB/RIF, or MTB culture;
- **Sputum-test-negative,** if none of the tests are positive AND
  - at least one test is known to be negative
  - patient was unable to produce a sputum sample

8.3 Diagnostic assessment outcome

The diagnostic assessment outcome will be defined for each study arm as the proportion of sputum-test-negative who are ACASI-test-negative as illustrated in Figure 2.

Reference Result: *any positive smear microscopy, Xpert/MTB/RIF, or MTB Culture from sputum samples collected on Day 1 and Day 8 visit defines tuberculosis-test-positive*

**ACASI* Response on Day 8** *ACASI test is defined by response to the following question asked using ACASI on Day 8: on day 1, you reported that you were unwell; compared to that day, has your illness worsened, remained the same, or improved?*		Sputum-test-positive	Sputum-test-negative
	ACASI-test-positive (worse or no change)	a	b
	ACASI-test negative (Improved)	c	d

Primary outcome: specificity, calculated by **d / (b+d)**

*Audio Computer Assisted Self-Interview (ACASI) in which the participant, after a how-to-use test session, responds to the prescribed question on a database-linked android tablet, without any human interaction, and in private.

-*+**Figure 2**: Assessing the diagnostic value of a change in symptoms from baseline to day 8

8.4 Analysis for assessing diagnostic impact of trial-of-antibiotics

We will perform comparisons (specified under section 7) of the proportion of sputum-test-negative participants who are also ACASI-test-negative (d/[b+d] in Figure 2) in trial-of-antibiotics (azithromycin and/or amoxicillin) arms to standard of care arm (Figure 2) (i.e a comparison of specificities with versus without trial-of-antibiotics using a generalised linear model (GLM) with binominal family, and (i) identity link to estimate risks differences and (ii) the GLM with log link to estimate risk ratios. If the outcome is rare or if GLM model with log link does not converge, we will use logistic regression to model odds and report odds ratios. All analyses will adjust for primary care centre based on where the participant was enrolled from (2 strata). For each comparison, we will report the point estimate, 95% CIs and p-values. Table shells for this analysis are shown in Appendix 3 for primary outcome, Appendix 4 for secondary outcome, and Appendix 5 for detailed diagnostic performance parameters.

9 Clinical impact of trial-of-antibiotics

Clinical impact of trial-of-antibiotics will be determined by comparing the proportion of participants experiencing at least one of the following adverse outcomes between arms: death, hospitalisation, TB misdiagnosis, HIV care loss to follow up, TB care loss to follow up.

9.1 Outcome definitions

The following are definitions of the components of this composite clinical outcome documented on day 29 (visit window: up to day 29+3 weeks):

Outcome component	Definition
death	death before or on Day 29
hospitalisation	being hospitalised for any reason at any point before or on Day 29
missed TB diagnosis	Participants not identified as TB positive at day 1 or day 8 but started on TB treatment based on either a day 29 sputum sample or routine care clinical decision made following study team referral on Day 29 visit

9.2 Analysis for assessing clinical impact of trial-of-antibiotics

For each component of the composite outcome, we will report cumulative incidence by study arm. We will perform comparisons (specified under section 7) of the proportion of participants experiencing the composite clinical outcome using a GLM with binomial family and, (i) identity link to estimate risks differences and (ii) log link to estimate risk ratios. If the outcome is rare or if GLM model with log link does not converge, we will use logistic regression to model odds and report odds ratios. All analyses will adjust for primary care centre based on where the participant was enrolled from (2 strata). For each comparison, we will report the point estimate, 95% CIs and p-values. Table shells for this analysis are shown in Appendix 3 for primary outcome, and Appendix 4 for secondary outcome.

10 Impact of trial-of-antibiotics on antimicrobial resistance (AMR)

Impact of trial-of-antibiotics on AMR will be determined by comparing, between arms, the proportion of participants meeting AMR positive definition at day 29.

10.1 Outcome definitions

We will define **AMR positive** as having nasopharyngeal isolates of *Streptococcus pneumoniae* that are resistant to any of the following commonly used antibiotics: ceftriaxone, amoxycillin, cefoxitin, azithromycin, and erythromycin as determined using disc diffusion

technique; and **AMR negative** as either (1) not isolating any *Streptococcus pneumoniae* or (2) isolating any *Streptococcus pneumoniae* that is not resistant to any of the assessed antibiotics. For each arm, and at both baseline and day 29, we will report proportion of AMR positive participants. The study outcome will be the proportion of AMR positive participants at day 29.

10.2 Analysis for assessing clinical impact of trial-of-antibiotics

To determine impact of trial-of-antibiotics on AMR, we will perform comparisons (specified under section 7) of the Day 29 proportion of AMR positive participants using a GLM with binomial family and, (i) identity link to estimate risks differences and (ii) log link to estimate risk ratios. If the outcome is rare or if GLM model with log link does not converge, we will use logistic regression to model odds and report odds ratios. All analyses will adjust for primary care centre based on where the participant was enrolled from (2 strata). For each comparison, we will report the point estimate, 95% CIs and p-values. Table shells for this analysis are shown in Appendix 4.

11 APPENDIX 1: Study Consort Diagram

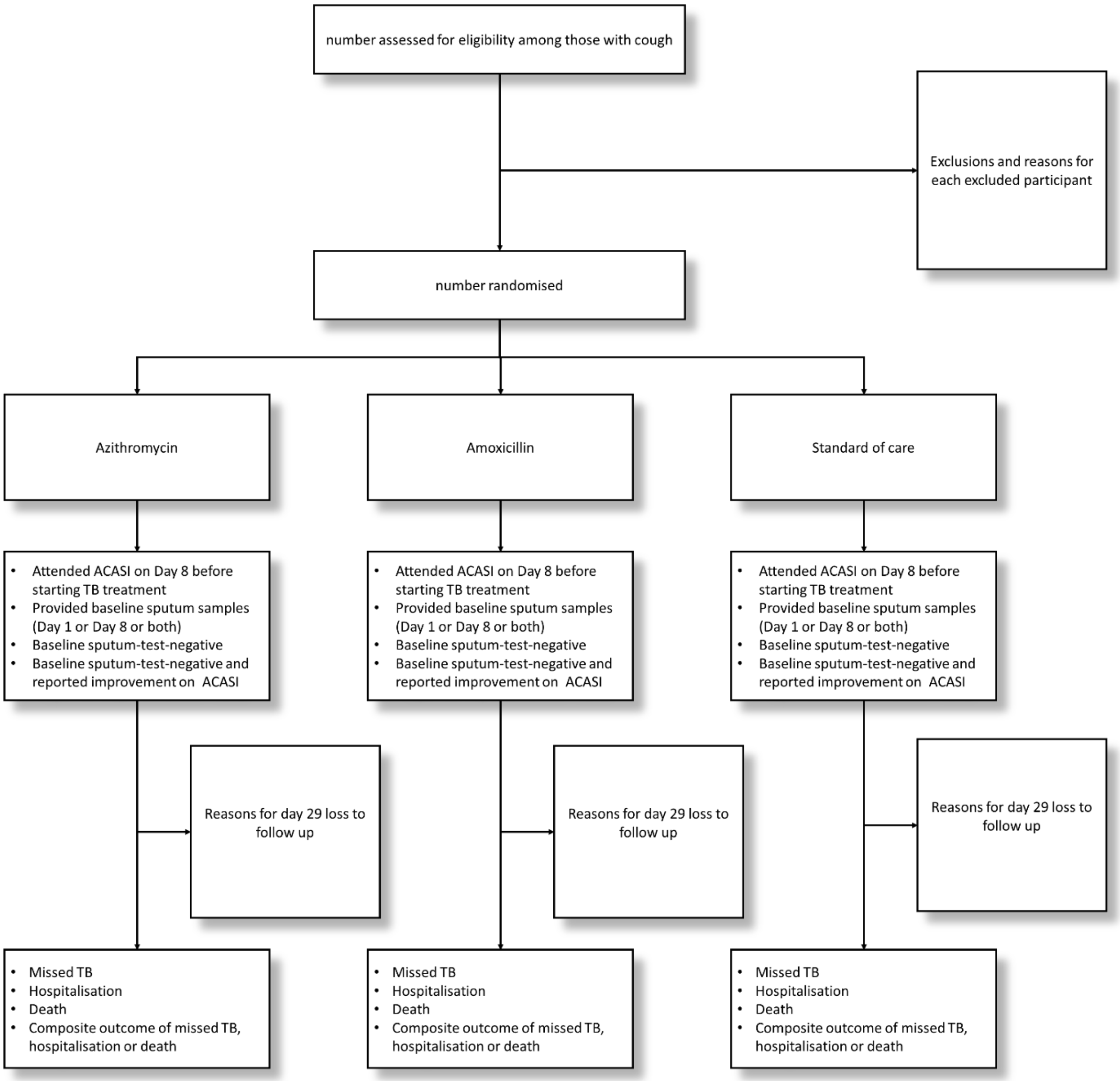

12 APPENDIX 2: Participant characteristics at baseline

		Amoxicillin and azithromycin			Azithromycin			Amoxicillin			Standard of care	
randomised (n, %)												
Site												
Limbe (n, %)												
Ndirande (n, %)												
Age in years (mean, SD)												
Gender												
Female (n, %)												
Male (n, %)												
Pregnant *(n, %)*												
Signs and symptoms												
Fever (n, %)												
Night sweats (n, %)												
Chest pain (n, %)												
Blood in sputum (n, %)												
Self-reported weight loss (n, %)												
BMI (mean, SD)												
Low BMI (defined as weight/height^2 of <19) (n, %)												
Previous TB *(n, %)*												
Months since last dose of TB treatment *(mean, sd)*												
HIV status												
HIV positive *(n, %)*												
HIV negative *(n, %)*												
HIV unknown *(n, %)*												
ART Status if HIV positive												
On ART *(n, %)*												
Not on ART *(n, %)*												
Years in education *(n, %)*												
AMR positive swab *(n, %)*												

13 APPENDIX 3: Results for primary outcomes

	Amoxicillin and azithromycin	Azithromycin	Amoxicillin	Standard of care
**Day 8 Primary outcome:** *proportion of sputum-TB-negative participants who report symptom improvement on Day 8 ACASI*				
Sputum-TB-positive (n, N)				
Sputum-TB-negative (n, N)				
Reported improvement on Day 8 ACASI (n, N)				
Reported improvement on Day 8 ACASI and were also Sputum-TB-negative				
n, N				
RD (95%CI), p-value*				*Reference*
RR (95%CI), p-value*				*Reference*
**Day 29 Primary outcome:** Proportion of participants experiencing missed TB, hospitalisation or death				
Missed TB (n, N)				
Hospitalisation (n, N)				
Death (n, N)				
Composite endpoint of missed TB, Hospitalisation and Death				
n, N				
RD (95%CI), p-value*				*Reference*
RR (95%CI), p-value*				*Reference*

RD risk difference RR risk ratio, CI confidence interval, ACASI Audio Computer Assisted Self-Interview

* adjusted for study site

Subgroup analyses for specificity

	Amoxicillin and azithromycin	Azithromycin	Amoxicillin	Standard of care
**Day 8 Primary outcome:** *proportion of sputum-TB-negative participants who report symptom improvement on Day 8 ACASI*				
OVERALL				
n, N				
RD (95%CI), p-value*				*Reference*
RR (95%CI), p-value*				*Reference*

	Amoxicillin and azithromycin	Azithromycin	Amoxicillin	Standard of care
**Day 8 Primary outcome:** *proportion of sputum-TB-negative participants who report symptom improvement on Day 8 ACASI*				
HIV POSITIVE (include previously diagnosed and newly diagnosed)				
n, N				
RD (95%CI), p-value*				*Reference*
RR (95%CI), p-value*				*Reference*
HIV NEGATIVE				
n, N				
RD (95%CI), p-value*				*Reference*
RR (95%CI), p-value*				*Reference*
HIV UNKNOWN				
n, N				
RD (95%CI), p-value*				*Reference*
RR (95%CI), p-value*				*Reference*
HIV POSITIVE ON ART				
n, N				
RD (95%CI), p-value*				*Reference*
RR (95%CI), p-value*				*Reference*
HIV POSITIVE NOT ON ART				
n, N				
RD (95%CI), p-value*				*Reference*
RR (95%CI), p-value*				*Reference*

14 APPENDIX 4: Results for secondary outcomes

	Amoxicillin and azithromycin	Azithromycin	Amoxicillin	Standard of care
**Day 8 Secondary outcome**				
Either sputum-TB-negative or no valid sputum result available (n, N)				
Reported improvement on Day 8 ACASI and were also either sputum-TB-negative or could not produce sputum				
n, N				
RD (95%CI), p-value*				*Reference*
RR (95%CI), p-value*				*Reference*
**Day 29 Secondary outcome**				
Antimicrobial resistance positive at Day 29 (n, %)				
n, N				
RD (95%CI), p-value*				*Reference*
RR (95%CI), p-value*				*Reference*

RD risk difference RR risk ratio, CI confidence interval, ACASI Audio Computer Assisted Self-Interview

* adjusted for study site

15 APPENDIX 5: Diagnostic performance of ACASI-reported change in symptoms against a sputum TB diagnostic reference

	Amoxicillin and azithromycin	Azithromycin	Amoxicillin	Standard of care
**Part A:** Participants with sputum test results				
Number of participants				
True positive (n, %)				
False negative (n, %)				
False positive (n, %)				
True negative (n, %)				
Prevalence (95% CI)				
Positive predictive value (95% CI)				
Negative predictive value (95% CI)				
Sensitivity (95% CI)				
Specificity (95% CI)				
ROC area (95% CI)				
**Part B:** All study participants included, treating those unable to produce sputum as reference test negative				
Number of participants				
True positive (n, %)				
False negative (n, %)				
False positive (n, %)				
True negative (n, %)				
Prevalence (95% CI)				
Positive predictive value (95% CI)				
Negative predictive value (95% CI)				
Sensitivity (95% CI)				
Specificity (95% CI)				
ROC area (95% CI)				

Ethics, Regulatory and Institutional review of the randomised trial

Letters of support for the randomised trial

1. Letter of support from the National Tuberculosis Program

The project was reviewed by the Malawi National Tuberculosis program (NTP) to ensure that it is addressing an important evidence gap. The NTP reviewed, provided feedback, and the following letter of support.

In reply please quote No: NTP/003/.....

Programme Manager
National TB Control Programme
Lilongwe
Telephone:
(+265) 1 756828
(+265) 1 750089

Fax: (+265) 1 757782

Communications should be addressed to:
The Programme Manager
National TB Control Programme

UNITY AND FREEDOM

Ministry of Health
National TB Control Programme
Community Health Sciences Unit
P/Bag 65
Lilongwe

30th January 2017

To: TO WHOM IT MAY CONCERN

RE: LETTER OF SUPPORT FROM THE NTP FOR TITUS DIVALA TO APPLY FOR A NEW RESEARCH PROJECT TO CONDUCT A STUDY ON A "TRIAL-OF-(NON-TB) ANTIBIOTICS" TO EXCLUDE TB AS A CAUSE OF PROLONGED COUGH IN PRIMARY CARE ATTENDEES:

The broad objective of the project is to investigate the effectiveness, safety, and broader impact of a trial-of-antibiotics to "rule out" tuberculosis (TB) among symptomatic adults greater than 16 years.

This approach is International Policy, a ubiquitous component of National TB Programme (NTP) diagnostic algorithms including Malawi NTP, and widely practiced at global level. However, there is minimal evidence-base on accuracy, and no previous research on broader clinical outcomes and antimicrobialresistance (AMR).

The purpose of this letter is to confirm that the National TB Control Programme highly supports Titus Divala in his project research application.

We look forward to getting updates on the progress of this important research project application.

Dr. James Mpunga

NTP PROGRAMME MANAGER

2. Letter of support from the Blantyre District Health office

The project was presented to the Blantyre District Health Office under whose authority the research sites belong to. The District Health Management team provided feedback (which was incorporated into the protocol) and issued the following letter of support.

Telephone: Blantyre 0 1875332 / 01 877 401
Fax: 01 875 430 / 01 872 551/01878 539

Communication should be addressed to:
The Director of Health and Social Services
0882002533 :gkawalazira@yahoo.co.uk

UNITY AND FREEDOM

In reply please quote No. ....

DISTRICT HEALTH OFFICE
P/BAG 66
BLANTYRE
MALAWI

Ref No: BT DHO/MED/9

5th April, 2018

The Chairman,
COMREC,
Private Bag 360,
BLANTYRE

Dear Sir,

RE: LETTER OF SUPPORT FOR A STUDY TITLED: "RANDOMIZED CONTROLLED TRIAL INVESTIGATING IF BENEFITS OF USING RESPONSE TO BROAD SPECTRUM ANTIBIOTICS AS AN EXCLUSION DIAGNOSTIC FOR TUBERCULOSIS IN PRIMARY CARE ADULT PATIENTS OUTWEIGH THE RISK OF ANTIMICROBIAL RESISTANCE".

Blantyre District Health Office renders its support to the above study which is to be conducted by Dr. Titus Divala as part of his PhD studies.

We look forward to the findings from this study and hope the findings will help in policy direction.

Yours Sincerely ,

Dr. Gift Kawalazira
DIRECTOR OF HEALTH AND SOCIAL SERVICES

District Health Officer
APR 2018
Private Bag 66
Blantyre

3. Ethical approvals for the randomised trial

The trial was reviewed and approved by the University of Malawi College of Medicine Research and Ethics Committee, the LSHTM Research Ethics Committee, and Regional Committee for Health and Research Ethics, NTNU-Midt, Norway. The following are certificates of approval.

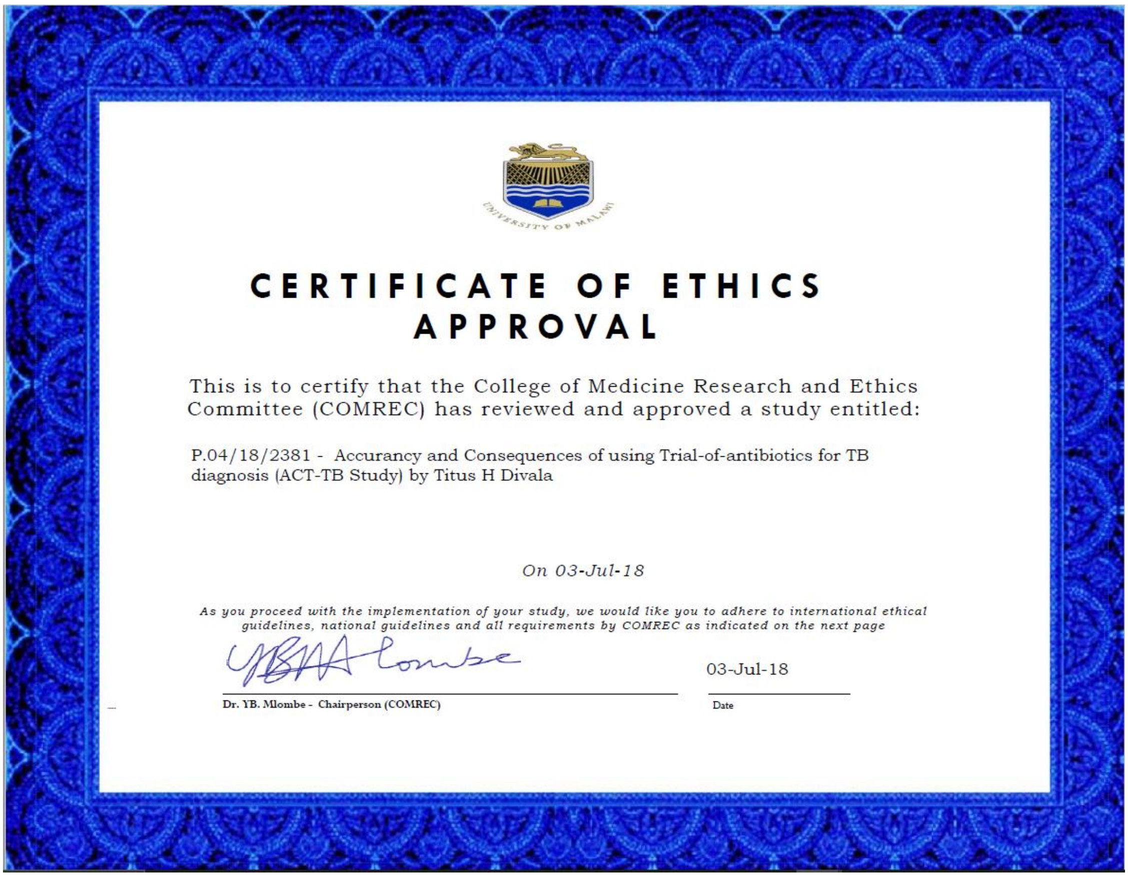

UNIVERSITY OF MALAWI

CERTIFICATE OF ETHICS APPROVAL

This is to certify that the College of Medicine Research and Ethics Committee (COMREC) has reviewed and approved a study entitled:

P.04/18/2381 - Accurancy and Consequences of using Trial-of-antibiotics for TB diagnosis (ACT-TB Study) by Titus H Divala

On 03-Jul-18

As you proceed with the implementation of your study, we would like you to adhere to international ethical guidelines, national guidelines and all requirements by COMREC as indicated on the next page

03-Jul-18

Dr. YB. Mlombe - Chairperson (COMREC)

Date

London School of Hygiene & Tropical Medicine
Keppel Street, London WC1E 7HT
United Kingdom
Switchboard: +44 (0)20 7636 8636

www.lshtm.ac.uk

LONDON SCHOOL of HYGIENE &TROPICAL MEDICINE

Observational / Interventions Research Ethics Committee

Dr Titus Divala
LSHTM

9 May 2018

Dear Titus,

Study Title: RCT investigating if benefits of using response to broad spectrum antibiotics as an exclusion diagnostic for tuberculosis in primary care adult patients outweigh the risk of antimicrobial resistance

LSHTM ethics ref: 15232

Thank you for your application for the above research, which has now been considered by the Interventions Committee.

Confirmation of ethical opinion

On behalf of the Committee, I am pleased to confirm a favourable ethical opinion for the above research on the basis described in the application form, protocol and supporting documentation, subject to the conditions specified below.

Conditions of the favourable opinion

Approval is dependent on local ethical approval having been received, where relevant.

Approved documents

The final list of documents reviewed and approved by the Committee is as follows:

Document Type	File Name	Date	Version
Safety Information	ACT_PackageInsertAzithromycin	14/03/2013	JUNE 2013
Safety Information	ACT_PackageInsertAmoxicillin	21/12/2015	DEC 2015
Sponsor Letter	2018-KEP-077_Sponsor Confirmation_13.03.18	13/03/2018	1
Other	GCP Cert_LSHTM_TDivala_21.03.18	21/03/2018	1
Investigator CV	ACT-CV1_TitusDivala	30/03/2018	1
Investigator CV	ACT-CV2_KatherineFielding	30/03/2018	1
Investigator CV	ACT-CV3_LizCorbett	30/03/2018	1
Information Sheet	ACT-20180330InformedConsentEnglish	30/03/2018	1.0
Information Sheet	ACT-20180330InformedConsentChichewa	30/03/2018	1.0
Protocol / Proposal	ACT-20180330Protocol	30/03/2018	1.0

After ethical review

The Chief Investigator (CI) or delegate is responsible for informing the ethics committee of any subsequent changes to the application. These must be submitted to the Committee for review using an Amendment form. Amendments must not be initiated before receipt of written favourable opinion from the committee.

The CI or delegate is also required to notify the ethics committee of any protocol violations and/or Suspected Unexpected Serious Adverse Reactions (SUSARs) which occur during the project by submitting a Serious Adverse Event form.

An annual report should be submitted to the committee using an Annual Report form on the anniversary of the approval of the study during the lifetime of the study.

At the end of the study, the CI or delegate must notify the committee using an End of Study form.

All aforementioned forms are available on the ethics online applications website and can only be submitted to the committee via the website at: http://leo.lshtm.ac.uk

Additional information is available at: www.lshtm.ac.uk/ethics

Yours sincerely,

Professor John DH Porter
Chair

ethics@lshtm.ac.uk
http://www.lshtm.ac.uk/ethics/

REK
REGIONALE KOMITEER FOR MEDISINSK OG HELSEFAGLIG FORSKNINGSETIKK

Region:	Saksbehandler:	Telefon:	Vår dato:	Vår referanse:
REK nord			06.11.2018	2018/1964/REK nord
			Deres dato:	Deres referanse:
			25.09.2018	

Vår referanse må oppgis ved alle henvendelser

Jon Øyvind Odland
Institutt for samfunnsmedisin

2018/1964 En kontrollert klinisk studie for å undersøke responsen til bredspekret antibiotika som en eksklusjonsdiagnose i allmenpraksis mot risiko for antibiotika resistens

Forskningsansvarlig institusjon: UiT Norges arktiske universitet
Prosjektleder: Jon Øyvind Odland

Vi viser til søknad om forhåndsgodkjenning av ovennevnte forskningsprosjekt. Søknaden ble behandlet av Regional komité for medisinsk og helsefaglig forskningsetikk (REK nord) i møtet 25.10.2018. Vurderingen er gjort med hjemmel i helseforskningsloven (hforsknl) § 10.

Prosjektleders prosjektomtale
Det skal undersøkes, ved hjelp av en randomisert, kontrollert studie om respons til bredspekret antibiotika kan brukes som eksklusjonsdiagnose for tuberkulose. Dette skal vurderes opp mot risiko for resistensutvikling knyttet til behandingen. Tre grupper (625 per gruppe) individuelt randomisert (1:1:1), med åpen informasjon undersøkes med standard diagnostisk tilnærming. Kun voksne deltakere, med full brukermedvirkning og informert samtykke. Studien kan forenkle dignostikk og klinikk for en utsatt gruppe mennesker. Studien skal i sin helhet foregå i Malawi. Alle etiske godkjenninger fra Malawi og London er vedlagt.

Komiteen vurderte at Hanne Husom Haukland var inhabil og hun fratrådte møtet da saken ble behandlet, jf. fvl. § 6.

Om prosjektet
Prosjektet er en del av en PhD. Studenten skal avlegge sin PhD i London.

Dette er et samarbeidsprosjekt mellom Universitetet i Malawi, London School of Hygiene and Tropical Medicine, og Universitetet i Tromsø.

Prosjektet skal gjennomføres i Malawi og er et «Tuberkulose initiativ» fra Helse Nord.

Det foreligger godkjenning fra London School of Hygiene and Tropical Medicine, og University of Malawi.

Data
Det skal hentes data fra pasientjournal og fra lokale poliklinikker. Data er standard medisinske opplysninger knyttet til infeksjoner og sykehistorie.

Nye helseopplysninger er respons på behandling av infeksjoner.

Besøksadresse:
MH-bygget UiT Norges arktiske universitet 9037 Tromsø

Telefon: 77646140
E-post: rek-nord@asp.uit.no
Web: http://helseforskning.etikkom.no/

All post og e-post som inngår i saksbehandlingen, bes adressert til REK nord og ikke til enkelte personer

Kindly address all mail and e-mails to the Regional Ethics Committee, REK nord, not to individual staff

Data avidentifiseres med koblingsnøkkel. Database oppbevares i 5 år i Malawi.

Deltakere/rekruttering
Voksne deltakere fordeles i tre grupper voksne deltakere (625 per gruppe), individuelt randomisert. Deltakere rekrutteres gjennom behandling på poliklinikkene.

REK har ingen innvendinger til prosjektet

Vedtak
REK har gjort en helhetlig forskningsetisk vurdering av alle prosjektets sider og godkjenner det med hjemmel i helseforskningsloven § 10. REK forutsetter at prosjektet også har godkjenning fra Malawi.

Sluttmelding og søknad om prosjektendring
Prosjektleder skal sende sluttmelding til REK nord på eget skjema senest 21.03.2022, jf. hfl. § 12. Prosjektleder skal sende søknad om prosjektendring til REK nord dersom det skal gjøres vesentlige endringer i forhold til de opplysninger som er gitt i søknaden, jf. hfl. § 11.

Klageadgang
Du kan klage på komiteens vedtak, jf. forvaltningsloven § 28 flg. Klagen sendes til REK nord. Klagefristen er tre uker fra du mottar dette brevet. Dersom vedtaket opprettholdes av REK nord, sendes klagen videre til Den nasjonale forskningsetiske komité for medisin og helsefag for endelig vurdering.

Med vennlig hilsen

May Britt Rossvoll
sekretariatsleder

Kopi til:jon.oyvind.odland@uit.no

4. Regulatory approval for the randomised trial

The Malawi Pharmacy and Medicines Regulatory Authority (formerly Pharmacy, Medicines, and Poisons Board) reviewed and issued a no-objection letter paving way for the implementation of the trial.

PHARMACY, MEDICINES & POISONS BOARD

Mission: To promote and improve the health of the population of Malawi through the regulation of Pharmacy Personnel, Pharmacy Businesses, Medicines and Allied Substances

ALL CORRESPONDENCE SHOULD BE ADDRESSED TO THE REGISTRAR

Head Office:
Off Paul Kagame/
Chilambula Road
P.O.Box 30241
Capital City
LILONGWE 3, MALAWI

Phone: (+265) 01 755 165
Fax : (+265) 01 755 204
Email: info@pmpb.mw
Web: www.pmpb.mw

PMPB/CTRC/III/14062018102
DATE: 4th July, 2018

Department of Infectious Disease Epidemiology
London School of Hygiene and Tropical Medicine
Keppel St
London

Attn.: Dr. Titus Divala

RE: ACCURACY AND CONSEQUENCES OF USING TRIAL-OF-ANTIBIOTICS FOR TB DIAGNOSIS (ACT-TB STUDY).

Refer to your application to register the above mentioned clinical trial with the Pharmacy, Medicines and Poisons Board (PMPB).

The Clinical Trial Review Committee (CTRC), at its meeting held on 22nd June, 2018, issued a **No Objection** to the implementation of the trial after members agreed that the nature of the trial was seen to be outside the scope of trials that should be regulated by PMPB through the CTRC.

Please contact the undersigned if there are any issues that need further clarification.

Yours faithfully,

M. Kawaye
ACTING REGISTRAR

PHARMACY MEDICINES & POISONS BOARD
REGISTRAR
04 JUL 2018
P.O. BOX 30241 LILONGWE 3
